# The apple GARP family gene MdHHO3 regulates the nitrate response and leaf senescence

**DOI:** 10.3389/fpls.2022.932767

**Published:** 2022-08-09

**Authors:** Binbin Wen, Xingyao Gong, Wenpeng Deng, Xiude Chen, Dongmei Li, Xiling Fu, Ling Li, Qiuping Tan

**Affiliations:** ^1^College of Horticulture Science and Engineering, Shandong Agricultural University, Tai’an, China; ^2^State Key Laboratory of Crop Biology, Shandong Agricultural University, Tai’an, China; ^3^Shandong Collaborative Innovation Center for Fruit and Vegetable Production With High Quality and Efficiency, Shandong Agricultural University, Tai’an, China

**Keywords:** apple, genome-wide, MdHHO3, nitrate response, nitrate deficiency, senescence

## Abstract

The regulation of plant gene expression by nitrate is a complex regulatory process. Here, we identified 90 GARP family genes in apples by genome-wide analysis. As a member of the GARP gene family, the expression of *MdHHO3* (*Malus domestica* H*YPERSENSITIVITY TO LOW PHOSPHATE-ELICITED PRIMARY ROOT SHORTENING1 HOMOLOG 3*) is upregulated under N (nitrogen) supply. The results of DNA-binding site analysis and electrophoretic mobility shift assays (EMSA) showed that MdHHO3 binds to the motif-containing GAATC. Furthermore, MdHHO3 binds to its promoter sequence and inhibits its activity. In addition, the overexpression of *MdHHO3* in apple calli resulted in less accumulation of nitrate in 35S:MdHHO3-GFP calli and downregulated the expression of the nitrate transport-related genes but upregulated the expression of the nitrate assimilation-related genes. Similarly, the expression of the nitrate transport-related genes was downregulated and the expression of the nitrate assimilation-related genes was upregulated in *MdHHO3* overexpression *Arabidopsis* and tobacco plants. Interaction experiments showed that MdHHO3 could bind to the promoter *MdNRT2.1* (*NITRATE TRANSPORTER 2.1*) and negatively regulate its expression. Moreover, the exposure of MdHHO3-overexpressing *Arabidopsis* and tobacco to nitrate deficiency resulted in an early senescence phenotype as compared to the WT plants. These results show that MdHHO3 can not only negatively regulate nitrate accumulation in response to nitrate but also promote early leaf senescence under nitrate deficiency. This information may be useful to further reveal the mechanism of the nitrate response and demonstrates that nitrate deficiency induces leaf senescence in apples.

## Introduction

The growth and reproduction of plants are closely related to changes in the environment. Therefore, plants can only grow and reproduce normally once they adapt to changes in the external environment. The soil nutrient status is one of the key environmental factors affecting plant growth and reproduction (Gallardo et al., 2006). In natural environments, plants often face N starvation conditions (Jackson and Caldwell, 1993; Miller and Cramer, 2004).

N is often a factor limiting plant growth and development. The regulation of plant gene expression by N is very complex, and some genes involved in the adaptive regulation of N starvation have been identified. Nitrate is the main N source absorbed by plants. When plants face a nitrate-deficient environment, the two families of nitrate transporters (NRT1 and NRT2) play important roles in high-affinity nitrate transport uptake (Nacry et al., 2013; Krapp et al., 2014). In the presence of a low external nitrate concentration, the dual-affinity transporter NRT1.1 is phosphorylated into a high-affinity transporter to participate in the absorption of a low concentration of nitrate (Miller et al., 2007; Ho et al., 2009). The NRT2 gene family members, *NRT2.1*, *NRT2.2*, *NRT2.4*, and *NRT2.5*, are involved in the absorption of nitrate at a low concentration, and among these, only *NRT2.1* showed a significant correlation between transcript abundance and high-affinity nitrate influx after nitrate supply to nitrate-deficient plants (Lezhneva et al., 2014; Kiba and Krapp, 2016). In addition, NRT2.1 plays a leading role in root nitrate absorption, and the activity of *Arabidopsis* high-affinity transport system (HATs) was decreased by 63–75% when *NRT2.1* was mutated (Li et al., 2007). However, overexpression of *NRT2.1* resulted in a significant increase in plant biomass and a 38% increase in yield (Orsel et al., 2019). The role of *NRT2.1* in the HATs supports its management of nitrate uptake in natural conditions, but the transcriptional repressors that regulate *NRT2* expression in a time- and nitrate concentration-dependent manner remain to be identified. Rice *NIGT1* (*NITRATE-INDUCIBLE GARP-TYPE TRANSCRIPTIONAL REPRESSOR1*) has been demonstrated to be involved in the nitrate response by encoding a high-affinity transporter gene (Sawaki et al., 2013). Further characterization of the *NIGT1* gene revealed that it encodes a transcriptional repressor of the regulatory network underlying the nitrate response (Sawaki et al., 2013). In addition, the binding region of *NIGT1* is conserved in the promoter region of *Arabidopsis NRT2.1* (Maeda et al., 2018), which suggests that *NIGT1* and its homologous genes may play similar roles in the N response.

N is an essential nutrient for plant growth and development and is the main component of amino acids, nucleotides, and chlorophyll, among other compounds. Therefore, N availability has a profound impact on plant productivity and growth. Nitrate in the soil is the main source of N that is absorbed by most plants, and its deficiency induces leaf senescence (Park et al., 2018). Plants adapt to N deficiency by regulating gene expression and metabolism to efficiently acquire and utilize the available N (Nacry et al., 2013). *NLA* (*NITROGEN LIMITATION ADAPTATION*) plays a key role in the process of leaf senescence induced by N deficiency (Kant et al., 2011). In addition, NLA protein can interact with ORE1 protein, the key gene for leaf senescence, to determine the leaf senescence process by regulating the stability of ORE1 proteins (Park et al., 2018). NRT1.5 plays an essential role in perceiving nitrate deficiency signals and inhibits leaf senescence induced by nitrate deficiency (Chen et al., 2021). NIGT1 hampers nitrate-induced positive regulation and reduces chlorophyll content in the *NIGT1* overexpressors (Sawaki et al., 2013). Since N is the main component of chlorophyll, *NIGT1* may be an important regulator to inhibit the absorption of nitrate and lead to the decline of chlorophyll. Chlorophyll degradation is the first important sign of leaf senescence. As a perennial deciduous fruit tree, the time of leaf senescence directly affects the yield and quality of the fruit (Woo et al., 2013; An et al., 2018b). Therefore, it is important to investigate the molecular mechanism of *NIGT1* in N starvation-induced leaf senescence for the maintenance of apple leaf function.

To reveal the complex molecular mechanism of gene regulation in the nitrate supply and nitrate starvation, we identified the function of the *MdHHO3* in this study. The supply of nitrate first rapidly induced the expression of *MdHHO3*, but longer exposure to nitrate limited the expression of this gene. Under nitrate-free conditions, the MdHHO3-overexpressing plants showed an early senescence phenotype. These results indicate that nitrate acts not only as a nutrient but also as a signal molecule and nitrate deficiency may generate signals to regulate the senescence of apple leaves. More significantly, the identification of the function of the *MdHHO3* gene clarifies the molecular mechanism of nitrate uptake and transport in apples and the adaptation of apples to a low-N environment, which provides a direction for the future breeding of this plant.

## Materials and methods

### Plant materials and growth conditions

The tissue culture plantlets of *Malus* × *domestica “*GL3” were grown on an MS medium containing 0.5 mg/L GA3, 0.5 mg/L 6-BA, and 0.1 mg/L NAA, and subcultured every 30 days. The plantlets subcultured for 30 days were selected to root in 1/2 MS medium containing 0.2 mg/L IAA. Then, the rooted apple plantlets were transferred to nursery pots for cultivation. In the hydroponic experiment, seedlings (*n* = 100) with the same growth parameters were planted on foam plates with 20 holes in each plate. Single *Malus* × *domestica “*GL3” plantlet was planted in each well and cultured with 1/2 Hoagland solution (Hoagland and Arnon, 1950) nutrient solution for 7 days. The nutrient solution was then replaced every 2 days with Hoagland nutrient solution. Once the plantlet grew 10 true leaves and to a height of approximately 12 cm, they were treated with distilled water for 7 days. The apple plantlets were grown at 25°C/23°C under long-day conditions (14 h of light:10 h of dark) with a relative humidity of 75% and a light intensity of 2,000 lx and were treated with various compounds. The apple calli (*Malus domestica*, Orin) were grown at 24°C in the dark and subcultured every 15 days. The *Arabidopsis thaliana* used in the study was Col-0, and the tobacco was *Nicotiana benthamiana*; both of these plants were grown at 25°C/23°C under long-day conditions (14 h of light:10 h of dark).

### Identification and phylogenetic analysis of the G2-like genes in apple

In this study, the *Arabidopsis* GARP-type protein sequences were used as a reference to construct the phylogenetic tree of apple GARP-type protein. The GARP-type gene family sequences from apple and *Arabidopsis* were downloaded from the Plant TF Database v. 4.0^1^ (Jin et al., 2017). All candidate GARP-type proteins were confirmed using the Conserved Domain Database^2^ and SMART.^3^ The confirmed GARP-type protein sequences from apple and *Arabidopsis* were used to construct a phylogenetic tree using MEGA-X software with the NJ method, a bootstrap value of 1,000, and other default parameters. We then used an online webserver named EvolView^4^ to clean up the phylogenetic tree. The protein sequences are listed in Supplementary Table 1. Multiple sequence alignments of the HHO3 protein of different species were performed using DNAMAN 6.0.3.99, and the HHO3 protein sequences of these different species are listed in Supplementary Table 2.

### RNA extraction and qRT-PCR analysis

Total RNA was extracted from apple, calli, *Arabidopsis*, and tobacco using an RNAprep Pure Kit (DP441, Tiangen, Beijing, China). First-strand cDNA was obtained using a cDNA reverse transcription kit (RR047A, Takara, Dalian, China). The primers used for qRT-PCR were designed with NCBI and synthesized by the Sangon Biotech Technology (Shanghai, China). The primers used are listed in Supplementary Table 3. SuperReal PreMix Plus (FP205, Tiangen, Beijing, China) was used for qRT-PCR. Three biological replicates and three technical replicates were analyzed using a CFX Connect™ Real-time System (Bio-Rad, Hercules, CA, United States). The qRT-PCR conditions were as follows: 95°C for 15 min and 40 cycles of 95°C for 10 s, 60°C for 20 s, and 72°C for 30 s. At the end of the reaction, a melting curve was obtained for each sample, and a transcription-level analysis was performed using the comparative Ct (2^–ΔΔ^ *^Ct^*) method. The primers used for qRT-PCR are shown in Supplementary Table 3.

### Measurement of the chlorophyll content

The chlorophyll content was determined as described above (Wen et al., 2019). In short, the leaves were beaten into small pieces with a punch and then submersed in 95% ethanol for 24 h until the leaves turned white. The absorbance was measured at wavelengths of 470, 649, and 665 nm using a spectrophotometer (UV-2600, Shimadzu, Shanghai, China).

### Subcellular localization analysis

The outer skin of the onion was removed, and the inner skin of the onion was peeled with a scalpel on a super clean workbench, cut into small 1-cm^2^ pieces, placed on MS solid medium, and cultured in the dark at 28°C for 24 h. The primers used for subcellular localization are shown in Supplementary Table 3. The complete sequence of *MdHHO3* without a stop codon was cloned and inserted into the pZP211 vector. The plasmids harboring 35S:MdHHO3-GFP and 35S:GFP were transformed into the *Agrobacterium tumefaciens* strain LBA4404 and infected onion epidermal cells. GFP fluorescence was observed under a Zeiss LSM880 microscope (Carl Zeiss, Oberkochen, Germany) and analyzed using the ZEN lite software.^5^ The subcellular localization analysis was repeated three times, and the results were similar.

### Generation of plant materials overexpressing *MdHHO3*

*MdHHO3* was cloned into the pRI101-AN vector containing the GFP tag sequence to form an overexpression plasmid. The primer is shown in Supplementary Table 3. The recombinant plasmid was transformed into *A. tumefaciens* GV3101 and LBA4404, and the recombinant vector was then transformed into calli, *Arabidopsis*, and tobacco using *A. tumefaciens* cells. Transgenic apple calli, *Arabidopsis*, and tobacco were obtained as described previously (Horsch et al., 1985; Clough and Bent, 1998; An et al., 2018a). Three transgenic lines were verified as MdHHO3-overexpressing plants.

### N deficiency assays

*Arabidopsis* and tobacco seeds were germinated on a 0.8% agar medium containing Murashige and Skoog salts and 30 g⋅L^–1^ sucrose. To induce leaf senescence in *Arabidopsis* seedlings in response to nitrate deficiency, 2-week-old *Arabidopsis* seedlings were grown on a nitrate-free medium (nitrate concentration: 0%) under long-day conditions (14 h of light:10 h of dark) at 24°C/22°C for 4 days. To induce leaf senescence in tobacco seedlings in response to nitrate deficiency, 4-week-old tobacco seedlings were grown in either nitrate-sufficient or a nitrate-free nutrient solution prepared with modified Hoagland’s solution (pH 5.8) for 3 weeks. After observing and recording the phenotype, the leaves of these plants were used to determine the chlorophyll content and the expression of senescence-related genes.

### Yeast one-hybrid assays

The *MdNRT2.1* promoter fragment (sequence of the fragment containing cis-acting elements) was inserted into the pAbAi vector to construct the reporter gene pBait-AbAi vector, and the full-length CDS of *MdHHO3* was cloned into the pGADT7 vector. The primers used in yeast one-hybrid assays are shown in Supplementary Table 3. To determine the minimum concentration of AbA (Aureobasidin A) that inhibits bait strain growth, the pAbAi plasmid was linearized by the restriction enzyme Bsp1191 (FD0124, Thermo Fisher Scientific™, Shanghai, China) and then transformed into the Y1H yeast strain, and the transformants were screened on SD/-Ura medium containing different concentrations of AbA. Single colonies were selected, and Matchmaker Insert Check PCR Mix 1 (Clontech) was used for colony PCR to verify the positive colonies. The minimum AbA concentration that inhibits the growth of the bait strain was then determined. The linearized pAbAi-MdNRT2.1 plasmid was co-transfected with the pGADT7-MdHHO3 plasmid into the Y1H yeast strain, and the yeast transformation product was then tested on medium (SD/-Ura/-Leu) containing an optimal concentration of AbA.

### Electrophoretic mobility shift assays

The full-length CDS of *MdHHO3* was cloned into the overexpression vector pGEX-4T-1 to obtain a recombinant plasmid. The MdHHO3 recombinant plasmid was expressed in *Escherichia coli* strain BL21 (Tiangen, Beijing, China) and purified using Glutathione-Sepharose Resin (CWbiotech, Beijing, China). The synthesis and labeling of the probe were performed by Sangon Biotech Co., Ltd. (Shanghai, China). The probes used for EMSA are shown in Supplementary Table 3. The purified fusion protein and biotin-labeled probes were incubated in a binding buffer at room temperature under dark conditions for 30 min. After incubation, a protein loading buffer was added, and the proteins were separated by polyacrylamide gel electrophoresis (Thermo Fisher Scientific, Shanghai, China).

### Dual luciferase assays

The promoter fragment of the *MdNRT2.1* gene (including the cis-acting site sequence) was cloned into the pGreenII 0800-LUC vector to generate a reporter vector. The full-length CDS of *MdHHO3* was inserted into the pGreenII 62-SK vector to construct the effector vector. The recombinant plasmid was transformed into the *Agrobacterium* strain GV3101. Tobacco leaves were injected with the *Agrobacterium* strain, cultivated in the dark for 12 h, and then placed in a light incubator. After 48 h, the injected tobacco leaves were collected and sprayed with D-Luciferin sodium salt (Sangon Biotech), and the fluorescence was then observed using an *in vivo* imaging system (Xenogen, Alameda, CA, United States).

### Statistical analyses

GraphPad Prism 6 software (GraphPad Software, La Jolla, CA, United States) was used for the drawing, and SPSS 19.0 software (SPSS, Chicago, IL, United States) was used for the statistical analyses. The significance of the differences was tested by *t*-tests (*P < 0.05, significant; ^**^P < 0.01, extremely significant).

## Results

### Identification and analysis of HHOs in apples

**FIGURE 1:**
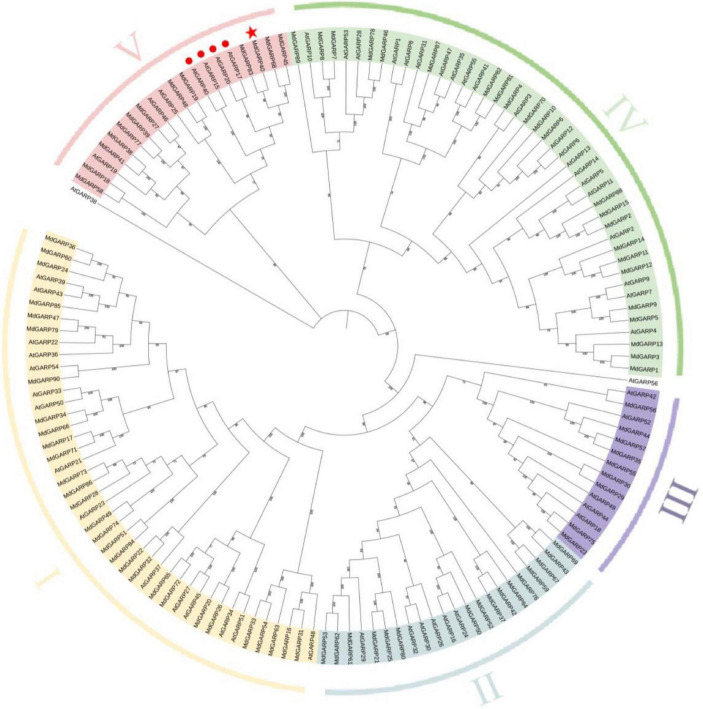
Phylogenetic tree of GARP-type proteins from apple and *Arabidopsis*. The phylogenetic tree was constructed using the NJ (neighbor-joining) algorithm. GARP proteins were clustered into five classes, and the five subfamilies are distinguished by different colors. The confirmed GARP-type protein sequences from apple and *Arabidopsis* were used to construct a phylogenetic tree using the MEGA software with the NJ method, a bootstrap value of 1,000, and other default parameters. The red star indicates MdHHO3(MdGARP40). The red circles indicate *Arabidopsis* four NIGT1s (NIGT1.1-1.4), among which AtGARP17, AtGARP20, AtGARP40, and AtGARP15 indicate AtHHO3/AtNIGT1.1, AtHHO2/AtNIGT1.2, AtHHO1/AtNIGT1.3, and AtHRS1/AtNIGT1.4, respectively. The protein sequences are listed in Supplementary Table 1.

To study the evolutionary relationship between the MdHHOs and AtHHOs genes, we constructed a rootless phylogenetic tree of apple GARP-type proteins using the *Arabidopsis* GARP-type protein sequences as a reference (Figure 1). A total of 90 GARP-type proteins were identified in the apple genome. The GARP-type gene family was classed into five groups (I–V) based on evolutionary relationships. Groups I–V contain 29, 16, 9, 23, and 13 genes, respectively. Among them, MdHHO3 was classified in the V group. In addition, the phylogenetic relationship between GARP-type proteins in apple and *Arabidopsis* suggests that apple MdHHO3 (MdGARP40) is the closest homolog of *Arabidopsis* AtHHO3/AtNIGT1.1 (AtGARP17) and AtHHO2/AtNIGT1.2 (AtGARP20).

### Identification of MdHHO3 in apples

**FIGURE 2:**
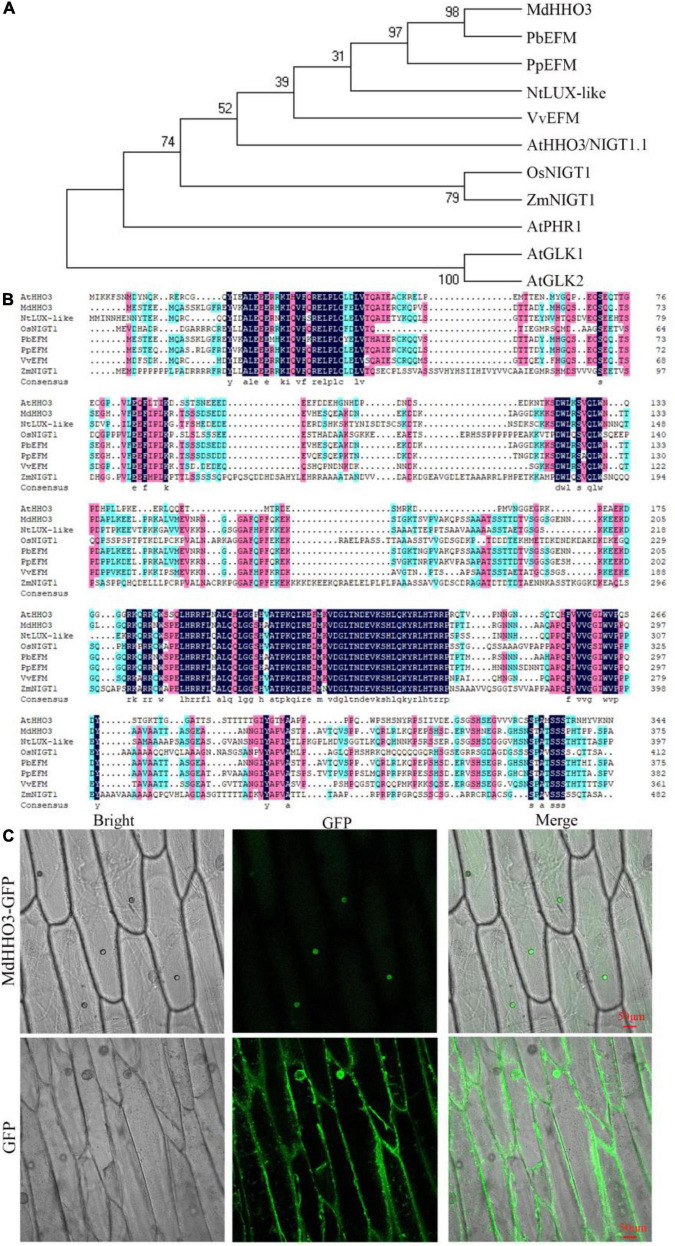
Phylogenetic analysis and subcellular localization of MdHHO3. **(A)** Phylogenetic tree analysis of HHOs of different species. The protein sequences of HHOs are listed in Supplementary Table 2. **(B)** Sequence alignment of 8 HHOs proteins. PbEFM (XP_009336613.1) in white pear (*Pyrus bretschneideri*), PpEFM (XP_007223224.2) in peach (*Prunus persica*), NtLUX-like (LUX ARRHYTHMO-like XP_016469710.1) in tobacco (*Nicotiana tabacum*), VvHHO3-like (AUZ62361.1) in grape (*Vitis vinifera*), AtHHO3/NIGT1.1 (NP_564236.1) in *Arabidopsis thaliana*, OsNIGT1 (XP_015623779.1) in rice (*Oryza sativa*), and ZmNIGT1 (PWZ44894.1) in maize (*Zea mays*) were used in the phylogenetic tree and sequence alignment analyses. **(C)** Subcellular localization of the 35S:MdHHO3-GFP fusion protein in onion; 35S: GFP was used as the control. GFP represents a green fluorescent protein.

Phylogenetic tree analyses showed that MdHHO3 was most closely related to PbEFM (Figure 2A). In addition, MdHHO3 is phylogenetically close to AtHHO3/NIGT1.1, OsNIGT1, and ZmNIGT1. Generally, phylogenetically closer orthologs may have similar functions. Sequence analysis showed that MdHHO3 contained 376 amino acids with a putative molecular weight of 41.44 kDa and a theoretical isoelectric point of 6.66. The multiple sequence alignment analyses showed that MdHHO3 had a myb-like DNA-binding domain and was similar to those of PbEFM (EARLY FLOWERING MYB PROTEIN) and PpEFM (Figure 2B). We constructed a 35S:MdHHO3-GFP vector and used the vector to transform onion epidermal cells. The subcellular localization of the 35S:MdHHO3-GFP fusion protein and the 35S:GFP protein was detected by fluorescence microscopy. The results showed that 35S:MdHHO3-GFP was located in the nucleus, whereas 35S:GFP was distributed throughout the whole cell (Figure 2C). These results suggest that MdHHO3, as a nuclear protein, plays a role in the response to nitrate.

### The expression of *MdHHO3* is induced by nitrate

**FIGURE 3:**
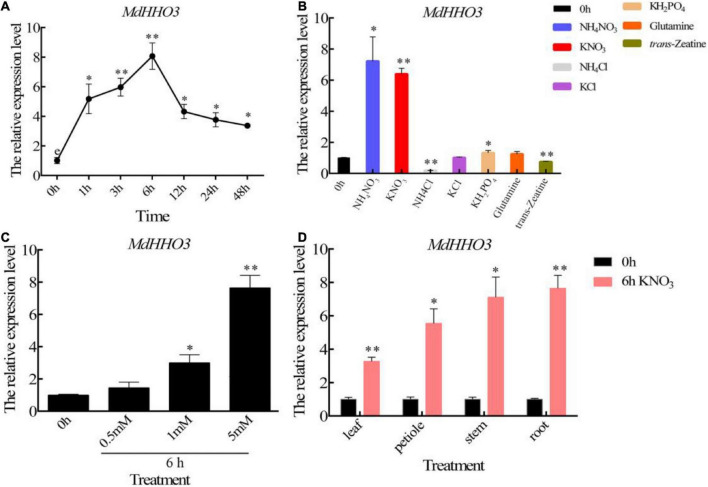
Nitrate specifically induces the expression of *MdHHO3*. **(A)** Time-dependent expression patterns of the *MdHHO3* gene after the supply of 5 mM KNO_3_. **(B)** Expression pattern of the *MdHHO3* gene after different N treatments. RNA was prepared from the roots of apple seedlings treated for 6 h with 5 mM NH_4_NO_3_, KNO_3_, NH_4_Cl, KCl, KH_2_PO_4_, and glutamine but not trans-zeatin (5 μM). **(C)** Expression of the *MdHHO3* gene induced by different nitrate concentrations. RNA was prepared from the roots of apple seedlings treated with 0, 0.5, 1, and 5 mM KNO_3_ for 6 h. **(D)** Nitrate induced the expression of *MdHHO3* in different plant tissues. Leaves, petioles, stems, and roots were collected from apple seedlings treated with 5 mM KNO_3_ for 6 h. *Malus* × *domestica* “GL3” seedlings grown for 1 week with distilled water were used as controls. Three biological replicates of each sample were included, and the data are expressed as the means ± SDs (*n* = 3). Significant differences were detected by a *t*-test: *P < 0.05 and ^**^P < 0.01.

To verify the temporal pattern of *MdHHO3* expression induced by nitrate supply, we supplied nitrate to apple seedlings treated with N starvation and performed the quantitative real-time polymerase chain reaction (qRT-PCR) analysis using RNA obtained from roots collected at different times. During nitrate treatment, the expression of *MdHHO3* was induced within 1 h, reached a peak approximately 6 h later, and then decreased, as shown in Figure 3A. To further verify that *MdHHO3* expression specifically responds to nitrate, we supplied ammonium, phosphorus, glutamate, and trans-zeatin and found that none of these compounds could induce the expression of *MdHHO3* (Figure 3B). We also found that the expression of *MdHHO3* could be induced by a low concentration of nitrate (Figure 3C), and the expression of *MdHHO3* was induced not only in the roots but also in the stems, leaves, and petioles (Figure 3D). These results indicate that MdHHO3 is a TF that is induced by nitrate and probably plays a role in nitrate-specific regulation in N response.

### *MdHHO3* binds to its own specific promoter sequence

**FIGURE 4:**
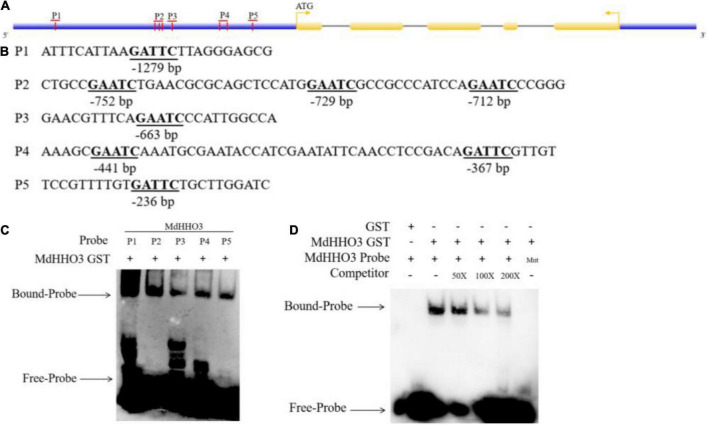
MdHHO3 binds to a specific sequence of its own promoter. **(A)** Diagram of the *MdHHO3* gene promoter region. A vertical bar represents the potential sites to which MdHHO3 may bind. **(B)** GAATC and GATTC motifs in the promoter region selected in the DNA-binding site. **(C)** The electrophoretic mobility shift assay (EMSA) showed that the MdHHO3-GST fusion protein binds to the labeled DNA probe. **(D)** EMSA results showed that MdHHO3 binds to the labeled probe of the MdHHO3 promoter. The MdHHO3-GST fusion protein was incubated with a labeled or mutated probe DNA fragment. The labeled probe used in this study is p1 (5′-ATTTCATTAAGATTCTTAGGGAGCG-3′). The unlabeled probes were used as a competitor. 50×, 100×, and 200× represent the rates of the competitor. “Mut” representing the mutated probe in which the 5′-GAATC-3′ motif was replaced by 5′-CAATG-3′. The – symbol indicates absence, and the + symbol indicates presence.

The nitrate supply experiment revealed that the expression of *MdHHO3* showed a decreasing trend with increases in the duration of nitrate supply. This finding suggests that *MdHHO3* may be a transcription inhibitor that inhibits its expression by binding to several sites of its own promoter. Previous studies have revealed the specific binding sequences of a few GARP family proteins (Waters et al., 2009). The DNA sequence selected in this study is GAATC. A promoter sequence analysis of *MdHHO3* identified a total of eight possible binding sites (GAATC) and these sites were named P1, P2, P3, P4, and P5 based on the distances between them (Figures 4A,B). To confirm that *MdHHO3* can bind to its own promoter, we performed EMSA experiments. As shown in Figure 4C, MdHHO3-GST fusion protein could bind to these sites. Furthermore, we used P1 as the binding probe according to the binding strength. The complex still bound to compete for probes at different concentrations but did not bind to mutant probes (5′-CAATG-3′) (Figure 4D). These results indicate that MdHHO3 can bind to the specific sequence of its own promoter.

### *MdHHO3* regulates the accumulation of nitrate

**FIGURE 5:**
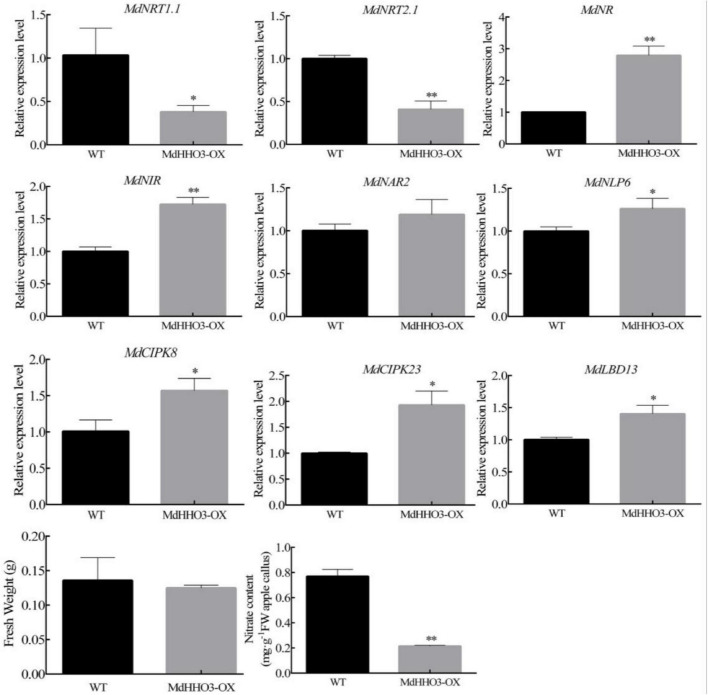
Expression of the nitrate regulatory genes (*MdNRT1.1*, *MdNRT2.1*, *MdNR*, *MdNIR*, *MdCIPK8*, *MdCIPK23*, *MdNAR2*, *MdNLP6*, and *MdLBD13*) in 2-week-old apple calli (WT, wild-type; MdHHO3-OX, with the MdHHO3 overexpression construct). The value for the WT control was set to 1. Three biological replicates of each sample were included, and the data are expressed as the means ± SDs (*n* = 3). Significant differences were detected by a *t*-test: *P < 0.05 and ^**^P < 0.01.

*MdHHO3* is an important regulator that regulates the response to nitrate. To verify the role of *MdHHO3* in the processes of nitrate transport and assimilation, overexpressed *MdHHO3* apple calli were obtained (Supplementary Figure 1). The transgenic and wild-type (WT) apple calli were cultured under normal conditions for 15 days, and the growth and fresh weight of the apple calli were then observed. The results revealed no significant difference in growth between the transgenic calli (*MdHHO3*) and the WT control (Figure 5). Because *MdHHO3* can be specifically induced by nitrate, *MdHHO3* may affect the accumulation of nitrate. Furthermore, we determined the content of nitrate in apple calli. As expected, the nitrate content in the overexpressed *MdHHO3* transgenic calli was significantly lower than that in the WT calli (Figure 5). These results indicate that *MdHHO3* may act as a negative regulator of nitrate accumulation.

The expression of nitrate transporter genes is usually used as a marker for nitrate uptake. To explore how *MdHHO3* regulates the uptake and accumulation of nitrate, the expression of these genes related to nitrate transportation and assimilation were examined. Surprisingly, the expression of the nitrate transport-related genes (*MdNRT1.1* and *MdNRT2.1*) was downregulated, whereas that of the nitrate assimilation-related genes (*MdNR* and *MdNIR*) were upregulated. In addition, the expression of five genes (*MdCIPK8*, *MdCIPK23*, *MdNAR2*, *MdNLP6*, and *MdLBD13*) that respond to nitrate was found to be upregulated (Figure 5). Interestingly, the expression of both the double-affinity transporter (*NRT1.1*) and high-affinity transporter (*NRT2.1*) was downregulated in the MdHHO3 transgenic calli. These results indicate that *MdHHO3* may inhibit the absorption of nitrate by inhibiting the expression of high-affinity transporters.

### Expression of *MdHHO3* inhibits nitrate transport in *Arabidopsis* and tobacco

**FIGURE 6:**
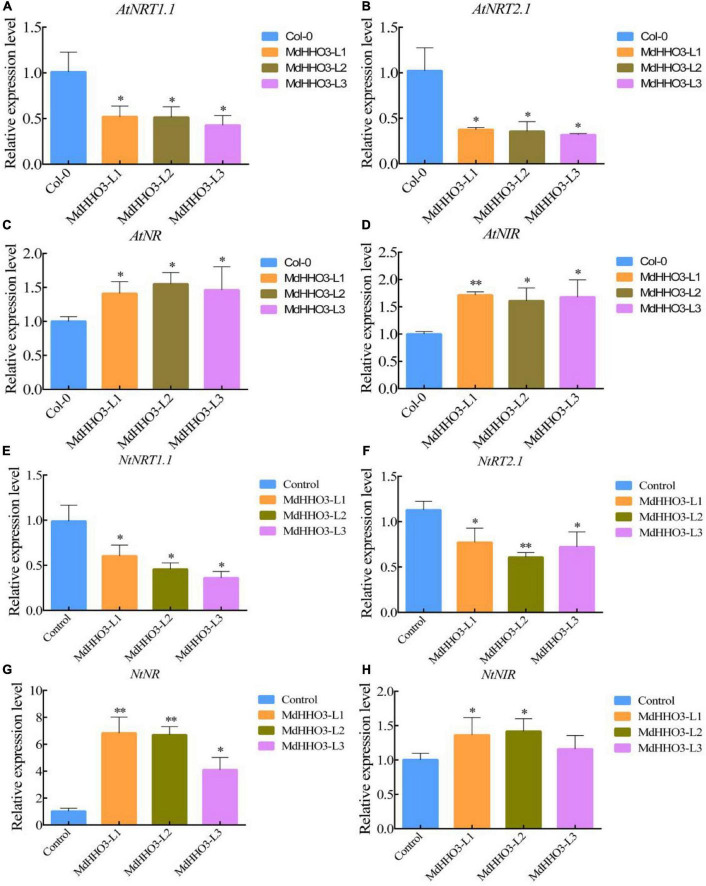
The overexpression of *MdHHO3* downregulates the expression of the nitrate transport-related genes and upregulates the expression of the nitrate assimilation-related genes in *Arabidopsis* and tobacco. **(A–D)** Expression of genes related to nitrate transport (*AtNRT1.1* and *AtNRT2.1*) and nitrate assimilation (*AtNR* and *AtNIR*) in the wild-type (Col-0) and *MdHHO3*-overexpressing *Arabidopsis*. **(E–H)** Expression of genes related to nitrate transport (*NtNRT1.1* and *NtNRT2.1*) and nitrate assimilation (*NtNR* and *NtNIR*) in wild-type (Control) and *MdHHO3*-overexpressing tobacco. Three biological replicates of each sample were included, and the data are expressed as the means ± SDs (*n* = 3). Significant differences were detected by a *t*-test: *P < 0.05 and ^**^P < 0.01.

To further verify the role of *MdHHO3* in nitrate transport and accumulation, the *MdHHO3* overexpression construct was generated and transformed individually into *Arabidopsis* and tobacco (Supplementary Figures 2, 3). The expression of the nitrate response-related genes *AtNRT1.1* and *AtNRT2.1* and the nitrate assimilation-related genes *AtNR* and *AtNIR* were then examined in *Arabidopsis thaliana*. In the overexpression lines, the expression of the tested nitrate response-related genes was downregulated, and the expression of the nitrate assimilation-related genes was upregulated (Figures 6A–D). Tobacco overexpressing *MdHHO3* showed a phenotype similar to that of MdHHO3-overexpressing *Arabidopsis*. Correspondingly, the expression of genes related to the nitrate response was downregulated, whereas that of genes related to nitrate assimilation was upregulated (Figures 6E–H). These results indicate that *MdHHO3* may act as a negative regulator of nitrate transport by inhibiting the accumulation of nitrate but improving enzyme activity in the nitrate assimilation pathway.

### *MdHHO3* directly regulates the *MdNRT2*.*1* gene transcription

**FIGURE 7:**
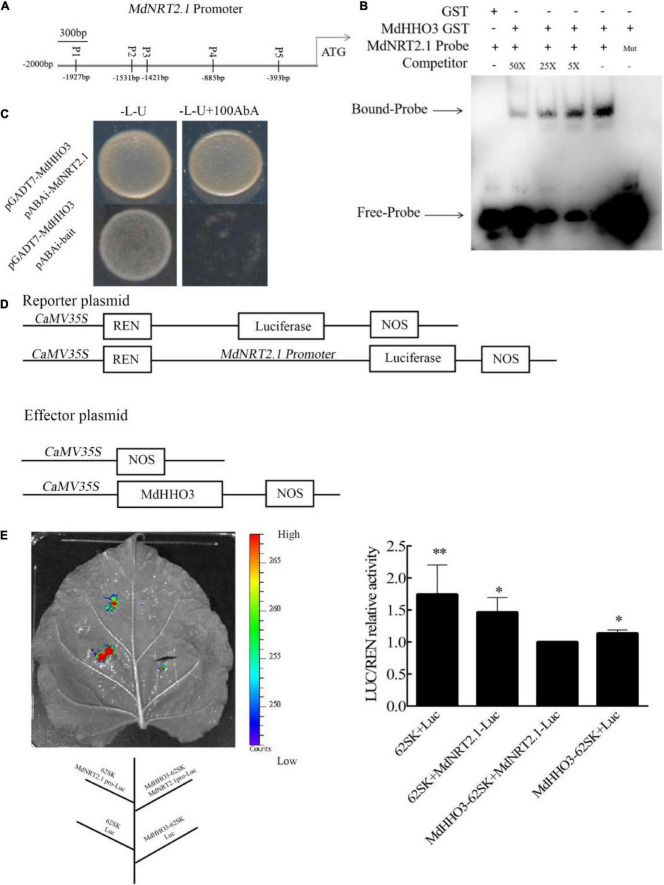
MdHHO3 inhibits the expression of *MdNRT2.1*. **(A)** Diagram of the *MdNRT2.1* gene promoter region. p1–p5 represents the potential sites to which MdHHO3 may bind. **(B)** The electrophoretic mobility shift assay (EMSA) showed that the MdHHO3-GST fusion protein binds to the *MdNRT2.1* promoter. The MdHHO3 fusion protein was incubated with a labeled or mutated probe DNA fragment. The labeled probe used in this study is p2 (5′-AACTCTTAAC**GAAT**CAATGGATGGA-3′). The unlabeled probes were used as a competitor. 50×, 100×, and 200× represent the rates of the competitor. “Mut” representing the mutated probe in which the 5′-GAATC-3′ motif was replaced by 5′-CAATG-3′. The – symbol indicates absence, and the + symbol indicates presence. **(C)** Yeast one-hybrid (Y1H) assay showing the interaction between MdHHO3 and the *MdNRT2.1* promoter. The empty PGADT7 was used as a control. The fused yeast strains were grown on SD/-Leu/-Ura and SD/-Leu/-Ura + 100/200/400 mM AbA media. **(D)** Structure of the reporter and effector vectors used in the dual luciferase assays. **(E)** A transient expression assay of tobacco leaves showed that MdHHO3 inhibited the expression of *MdNRT2.1*. Quantitative analysis of LUC/REN activity. An empty vector was used as the control. The data are expressed as the means ± SDs (*n* = 3). Significant differences were detected by a *t*-test: *P < 0.05 and **P < 0.01.

As a high-affinity transporter, *NRT2.1* plays an important role in nitrate transport. The expression of *NRT2.1* in MdHHO3-overexpressing apple calli, *Arabidopsis*, and tobacco was inhibited (Figures 5, 6B,F), which suggests that a transcriptional repressor might regulate its expression. An analysis of the promoter sequence of *NRT2.1* showed that it contains a sequence consistent with that of MdHHO3 (5′-GAATC-3′), and these sequences were named P1, P2, P3, P4, and P5 based on the distances between them (Figure 7A). To examine whether MdHHO3 regulates *NRT2.1*, an *in vivo* Y1H assay confirmed the interaction of the MdHHO3 protein and the *MdNRT2.1* promoter. The full-length CDS of *MdHHO3* and the *NRT2.1* gene promoter fragment containing the 5′-GAATC-3′ motif were cloned to the PGADT7 and pAbAi vectors, respectively. The yeast strains co-transformed with pGADT7-MdHHO3 and pABAi-MdNRT2.1 grew normally in a selective medium, whereas the growth of the control yeast strain with the empty vector was inhibited, which indicated the existence of a direct interaction between the MdHHO3 and *MdNRT2.1* (Figure 7C). To further examine the role of MdHHO3 in regulating the *NRT2.1* expression, MdHHO3 sites in the *NRT2.1* gene promoter were investigated *in vitro* using an EMSA with an integrated MdHHO3 protein. As shown in Supplementary Figure 4, five conserved motifs were putative MdHHO3-binding sites. According to the binding strength, we used P2 as the binding probe. In addition, the MdHHO3-GST fusion protein bound to the MdNRT2.1 probe (5′-GAATC-3′) but not to the mutated probe (5′-CAATG-3′), and the binding disappeared as the concentration of the competitive probe increased (Figure 7B).

Furthermore, to further confirm the direct regulation of *MdNRT2.1* expression by MdHHO3, we constructed a dual-effect reporter system with *MdHHO3* as the effector gene and *MdNRT2.1* as the luciferase reporter gene (Figure 7D). The recombinant fusion plasmid of the *MdHHO3* gene and the *MdNRT2.1* promoter fragment containing the 5′-GAATC-3′ motif were injected into tobacco leaves. Clearly, the LUC activity of tobacco co-injected with the *MdHHO3* and *MdNRT2.1* promoters was lower than that of the control group (Figure 7E). These results indicate that MdHHO3 can bind to the promoter of the *NRT2.1* gene and inhibit its expression.

### *MdHHO3* promotes leaf senescence induced by nitrate deficiency

**FIGURE 8:**
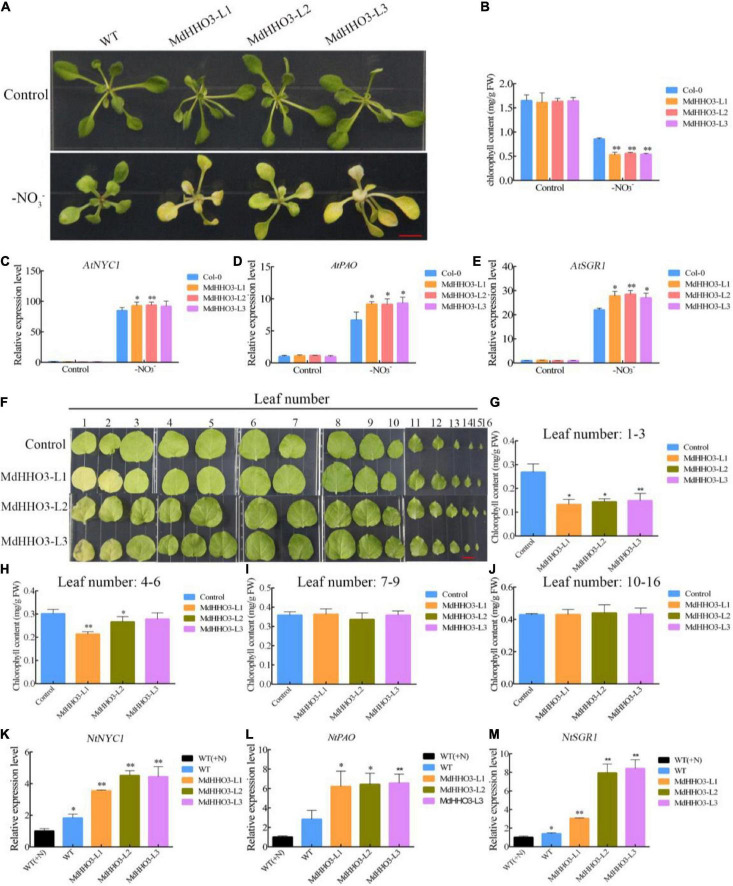
Overexpression of *MdHHO3* causes early senescence in *Arabidopsis* and tobacco. **(A)** Phenotypes of wild-type (Control) and transgenic *Arabidopsis* plants (*MdHHO3*) after 4 days of nitrate deficiency treatment. **(B)** Total chlorophyll content in *Arabidopsis* leaves. **(C–E)** Expression of the senescence-related genes (*AtSGR1*, *AtNYC1*, and *AtPAO*) in *Arabidopsis* after exposure to nitrate deficiency for 4 days. **(F)** Phenotypes of wild-type (Control) and transgenic tobacco (*MdHHO3*) leaves after 30 days of nitrate deficiency treatment. **(G–J)** Total chlorophyll content in tobacco leaves 1–3, 4–6, 7–9, and 10–16. **(K–M)** Expression of the senescence-related genes (*NtSGR1*, *NtNYC1*, and *NtPAO*) in tobacco after exposure to nitrate deficiency for 30 days. The data are expressed as the means ± SDs (*n* = 3). Significant differences were detected by a *t*-test: *P < 0.05 and **P < 0.01.

Because of nitrate deficiency-induced leaf senescence, MdHHO3 negatively regulates the nitrate starvation response gene *MdNRT2.1*. Therefore, we speculate that MdHHO3 may play a key role in leaf senescence induced by nitrate deficiency. As expected, nitrate deficiency significantly induced leaf senescence in 2-week-old *Arabidopsis* after 4 days of treatment under long-day conditions (14 of light:10 h of dark) (Figure 8A). Correspondingly, the chlorophyll content of MdHHO3-overexpressing *Arabidopsis* was significantly lower than that of the WT control (Figure 8B). During the process of nitrate deficiency-induced leaf senescence, the chlorophyll catabolism-related genes *AtSGR1* (Shimoda et al., 2016), *AtNYC1* (Kusaba et al., 2007), and *AtPAO* (Pruzinská et al., 2005) were significantly upregulated, and the overexpression of *MdHHO3* substantially increased the expression of these three genes (Figures 8C–E). However, under nitrate supply conditions, the overexpression of *MdHHO3* did not significantly induce the expression of these genes, and no significant change in the chlorophyll contents was detected (Supplementary Figures 2C–G). These observations showed that MdHHO3 promotes the induction of leaf senescence in response to nitrate deficiency.

Considering that *Arabidopsis* NIGT1 directly represses the expression of cytokinin synthase enzyme genes thereby leading to a semi-dwarf phenotype in the *NIGT1.1* overexpressing *Arabidopsis* (Maeda et al., 2018). We examined the expression levels of the high-affinity nitrate transporter genes (*NRT2.1*, *NRT2.4*, and *NRT2.5*), as well as the cytokinin synthesis enzyme genes (*CYP735A2*, *IP3*, and *ARR7*) in the wild-type and *MdHHO3* transgenic lines (Supplementary Figure 5). The expression of the high-affinity nitrate transporter genes (*AtNRT2.1*, *AtNRT2.4*, and *AtNRT2.5*) were downregulated in the *MdHHO3*-overexpressing *Arabidopsis* plants. Under nitrate-free conditions, the expression of the high-affinity transporter genes was upregulated in both the wild-type control and *MdHHO3*-overexpressing *Arabidopsis* plants. However, the expression level of these three genes in wild-type control was significantly higher than that in the *MdHHO3*-overexpressing *Arabidopsis*. This suggests that *MdHHO3* inhibits the expression of the high-affinity transporter genes more significantly under N-deficient conditions. In addition, we examined the expression levels of genes (*AtCYP735A2*, *AtIPT3*, and *AtARR7*) related to cytokinin synthesis. Under N supply conditions, we did not find the effect of overexpression of *MdHHO3* on the expression of the cytokinin synthesis-related genes. However, during N deficiency-induced leaf senescence, the expression levels of these three cytokinin synthesis-related genes decreased in both *MdHHO3*-overexpressing and wild-type control plants, but more significantly in *MdHHO3*-overexpressing plants. These results suggest that nitrate deficiency inhibits the expression of the cytokinin synthesis-related genes, and *MdHHO3* may play an important role in cytokinin synthesis, but further studies are needed.

In a similar experiment, transgenic tobacco seedlings were generated with an *MdHHO3* overexpression construct to further investigate the function of *MdHHO3*. As shown in Figure 8F, plants with different leaf numbers produced different senescence phenotypes after exposure to nitrate deficiency. We compared the chlorophyll content of leaves 1–3, 4–6, 7–9, and 10–16 between the overexpressed plants and the WT control. The changes in the chlorophyll content detected in leaves 1–3 and 4–6 were very similar, which showed that the overexpression of *MdHHO3* resulted in lower chlorophyll content, but no significant difference in the chlorophyll content was found in leaves 7–9 and 10–16 (Figures 8G–J). During the process, we monitored the expression levels of the senescence-related genes *NtSGR1*, *NtNYC1*, and *NtPAO*, which may be the target genes of *MdHHO3* under nitrate-free conditions (Figures 8K–M). The expression of these three target genes was induced by nitrate deficiency in the WT control. The induction level in the MdHHO3-overexpressing plants was 2–4-fold higher than that in the WT plants. The expression levels of these senescence-related genes were consistent with the leaf senescence phenotype. These results indicate that nitrate deficiency induces the expression of the senescence-related genes in MdHHO3-overexpressing plants and that *MdHHO3* is a positive regulator of nitrate deficiency-induced leaf senescence.

## Discussion

Nitrate serves as a signaling molecule, and its regulation of plant gene expression is very complicated (Tsay et al., 2011). The supply of nitrate can induce the expression of genes related to nitrate transport and assimilation (Scheible et al., 2004). In addition, nitrate is an important N source that plants absorb from the soil, and N deficiency induces leaf senescence (Agüera et al., 2010; Krapp et al., 2014). Therefore, revealing the complex molecular mechanism of nitrate absorption by fruit trees and exploring the network through which nitrate deficiency regulates leaf senescence are of great significance to apple production.

GARPs are transcription factors involved in nutritional responses, and many GARPs have been identified in several plants (Safi et al., 2017). For example, 56 GARP family genes have been identified in *Arabidopsis* (Riechmann et al., 2000), 52 GARP family genes have been found in rice (Zeng et al., 2018), and 59 GARP family genes have been detected in maize (Liu et al., 2016). In this study, the GARP family in apple contains 90 genes and is divided into five groups based on their evolutionary relationship. *MdHHO3* belongs to the V subfamily of the GARP gene family and is the closest evolutionary relationship with *Arabidopsis At1g25550* (*AtHHO3/AtNIGT1.1*) and *At1g68670* (*AtHHO2/AtNIGT1.2*). Previous studies have shown that *Arabidopsis AtHHO3/AtNIGT1.1* and *AtHHO2/AtNIGT1.2* are induced by nitrate treatment (Maeda et al., 2018). In *Malus domestica*, *MdHHO3* was upregulated after nitrate treatment, but downregulated after prolonged nitrate treatment. These results suggested that metabolites downstream of the N metabolism pathway may trigger this negative feedback reaction (Loqué et al., 2003; Nazoa et al., 2003). However, this study revealed that the promoter sequence of the *MdHHO3* gene contains its specific binding motif (5′-GAATC-3′). We speculate that over prolonged treatment, *MdHHO3* may bind not only to the promoter of downstream genes but also to its own promoter motif, which results in autorepression. In addition, MdHHO3 was found to act as a potential negative regulator in the nitrate response by regulating its own transcription and the nitrate transporter genes (Figures 1, 3, 4). Previous studies have shown that conserved N-terminal CCD (coiled-coil domain) is necessary for NIGT1-NIGT1 interaction (Ueda et al., 2020). In addition, the NIGT1 protein form a dimer *via* the N-terminal CCD to recognize different types of DNA sequences (Ueda et al., 2020). However, in our study, no conserved CCD was found in MdHHO3 protein. This indicated that the mode of regulation of nutrient responses and target genes by MdHHO3 may differ from that of other NIGT1 family proteins. Therefore, we speculate that there may be additional amino acid residues on the surface of MdHHO3 protein that bind to GAATC or GATTC motifs and effectively repress *MdHHO3* expression (Yanagisawa, 2013). These results indicate that the response of nitrate in plants involves not only the regulation of the upstream and downstream genes but also the self-regulation of genes. The regulatory network may be more complex than previously expected.

Subcellular localization analysis is an important method for understanding the specific location of fusion proteins in cells. In rice, the NIGT1-GFP fusion protein is predominantly located in the nucleus (Sawaki et al., 2013). Similarly, the NIGT1-GFP fusion protein is also localized in the nuclei of transgenic *Arabidopsis* plants overexpressing NIGT1-GFP, and its localization pattern is not altered by the availability of nitrate (Kiba et al., 2018). The subcellular localization analysis performed in this study indicated that the MdHHO3-GFP fusion protein is localized in the nucleus. This localization further indicates that HHO3, as a nuclear protein, is involved in the transcriptional regulation of nitrate.

Nitrate is the main N source of most land plants (Sawaki et al., 2013). The regulation of gene expression induced by nitrate serves as the foundation for plant growth, but the regulatory mechanism of nitrate is largely unknown. Based on our findings, we propose that *MdHHO3* may be a key regulator of nitrate-responsive transcription. As a regulator of nitrate-responsive transcription, *HHO3* may play a critical role in determining the capacity of nitrate transport, assimilation, and accumulation. In this study, we found that the overexpression of *MdHHO3* resulted in a decrease in the nitrate content in apple calli. Correspondingly, the expression of the nitrate transporter gene was significantly downregulated in the presence of the *MdHHO3* overexpression construct compared with the level found in the WT control. In contrast, the expression of the nitrate assimilation gene was significantly upregulated in the presence of the *MdHHO3* overexpression construct compared with that found with the WT control. Similarly, *Arabidopsis* and tobacco plants with *MdHHO3* overexpression also showed downregulated and upregulated expression of the nitrate transport- and nitrate assimilation-related genes, respectively. Because the nitrate content could be a marker of N utilization efficiency (Chardon et al., 2010), the overexpression of *MdHHO3* may negatively affect the N utilization efficiency by inhibiting the transport of nitrate and thus reducing the nitrate content. The content of nitrate directly affects the activity of N assimilating enzymes (Balotf et al., 2016). Previous studies have shown that under nitrate deficiency conditions, plants activate a high-affinity transport system, and thus increase nitrate reductase (NR) activity and mRNA expression (Tischner, 2000; Debouba et al., 2007). However, the expression of the *NR* and *NIR* genes was strongly induced by the overexpression of *MdHHO3*. It is possible that the overexpression of *MdHHO3* inhibits the uptake and transport of nitrate, which is equivalent to the results observed in cells with an N-deficient environment.

*Arabidopsis NRT2.1* is a typical nitrate-inducing gene that is essential for plant growth at low nitrate concentrations (Li et al., 2007). Several genes involved in the regulation of *NRT2.1* expression have been identified. NIN-like protein 7 (NLP7) is involved in the response of plants to nitrate and can bind to the *NRT2.1* promoter to enhance its expression (Marchive et al., 2013; Maeda et al., 2018). BT1/BT2 acts as a nitrate inhibitor to negatively regulate the expression of *NRT2.1* and reduce the utilization efficiency of N under low nitrate concentrations (Araus et al., 2016). AtTCP20 could bind to the promoter of *NRT2.1* and play a key role in the nitrate response (Guan et al., 2014), and TGA1 can directly bind to the promoter of *NRT2.1* and promote its expression (Alvarez et al., 2014). In this study, a series of assays showed that *MdHHO3* can also bind to the promoter of *NRT2.1* and inhibit its expression (Figure 7). *NRT2.1* is a typical nitrate-induced gene, which is indispensable for plant growth under low-nitrate concentration (Li et al., 2007). The expression of NRT2.1 was rapidly induced after supplying nitrate to nitrate deficient-cultivated plants, but was suppressed after a few hours (Zhuo et al., 1999), indicating that there was a transcriptional repress regulatory mechanism. Previous studies have shown that HHOs as important regulators of N starvation responses and play an important role in N starvation responses (Kiba et al., 2018). These results suggested that MdHHO3 may be a transcriptional repressor.

Leaf senescence is a complex regulatory process that serves as a comprehensive response to endogenous development and external environmental signals (Woo et al., 2004; Meng et al., 2016). Therefore, some genes involved in the response to environmental changes may be involved in leaf senescence. N is an important macronutrient absorbed by plants, and its deficiency induces leaf senescence (Schulte auf ’m Erley et al., 2007; Agüera et al., 2010). Some genes involved in N-deficiency-induced leaf senescence have been identified. Previous studies have shown that NRT1.7, a phloem nitrate transporter, delays N deficiency-induced leaf senescence (Fan et al., 2009). NLA, a N-limited adaptation regulator, regulates nitrate deficiency-induced leaf senescence by regulating the homeostasis of the ORE1 proteins (Park et al., 2018). The induction of leaf senescence by N deficiency is well-known, but its potential regulatory mechanism remains largely unknown. In our study, we found that the expression of *MdHHO3* and the chlorophyll catabolism-related genes (*MdNYC1*, *MdPAO*, and *MdSGR1*) were significantly upregulated during N deficiency-induced leaf senescence (Supplementary Figure 6). Furthermore, under nitrate deficiency conditions, *MdHHO3-*overexpressing *Arabidopsis* and tobacco showed early senescence symptoms and a lower chlorophyll level than the WT controls, and a series of genes related to leaf senescence also showed higher expression levels in the overexpressing plants than in the WT controls. The results indicated that *MdHHO3* may participate in nitrate deficiency-induced chlorophyll catabolism, but this hypothesis needs further research. In addition, another study found that N supply fails to induce early leaf senescence in *Arabidopsis* overexpressing *MdHHO3* (Supplementary Figure 3). These results suggest that MdHHO3 may be a positive regulator of N deficiency-induced leaf senescence.

**FIGURE 9:**
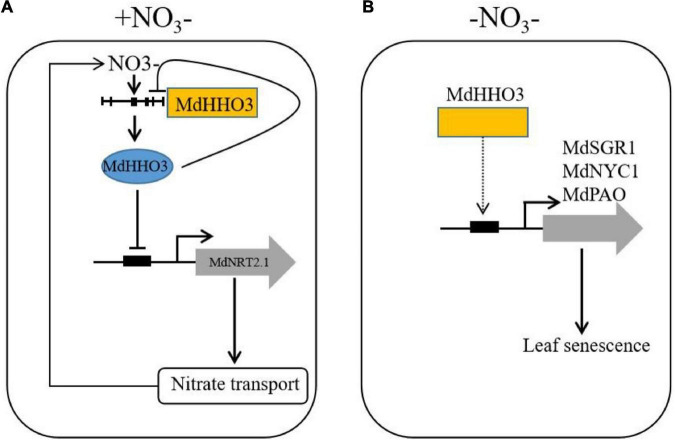
Working model of the function of *MdHHO3* in promoting leaf senescence in response to nitrate. **(A)** Transcriptional regulatory model of the modulation of nitrate uptake. Under the condition of nitrate supply, nitrate activates the *MdHHO3* clade gene promoters and is then repressed by the expression of *MdHHO3*. MdHHO3 binds to the *MdNRT2.1* promoter and inhibits its expression. **(B)** Model of *MdHHO3* regulating leaf senescence. In the absence of nitrate, *MdHHO3* regulates the expression of the senescence-related genes and thus promotes leaf senescence.

A working model was proposed based on the results of this study (Figure 9). Under nitrate supply, MdHHO3 binds to the promoter of *MdNRT2.1* and inhibits its expression. With prolonged nitrate supply, *MdHHO3* binds to its own promoter and inhibits its expression to reduce the nitrate content. Under nitrate deficiency conditions, *MdHHO3* promoted leaf senescence induced by N deficiency and the expression of senescence related genes. These results might help us better understand the regulatory mechanisms of nitrate transport and nitrate deficiency-induced leaf senescence.

## Data availability statement

The original contributions presented in the study are included in the article/Supplementary material, further inquiries can be directed to the corresponding authors.

## Author contributions

LL, QT, and BW designed the study. WD, XG, XC, DL, and XF performed the experiments and analyzed the data. BW wrote and revised the manuscript. All authors contributed to the article and approved the submitted version.

## Footnotes

1
http://planttfdb.gao-lab.org/

2
http://www.ncbi.nlm.nih.gov/Structure/cdd/wrpsb.cgi

3
http://smart.embl-heidelberg.de/

4
https://www.evolgenius.info/

5
https://www.zeiss.de/corporate/home.html
