# Supplementary material for: The apple GARP family gene MdHHO3 regulates the nitrate response and leaf senescence
Source: Front Plant Sci. 2022 Aug 9;13:932767. doi: 10.3389/fpls.2022.932767 (PMC9398197; doi:10.3389/fpls.2022.932767)
Supplement: Supplementary file 1 [file Data_Sheet_1.doc]

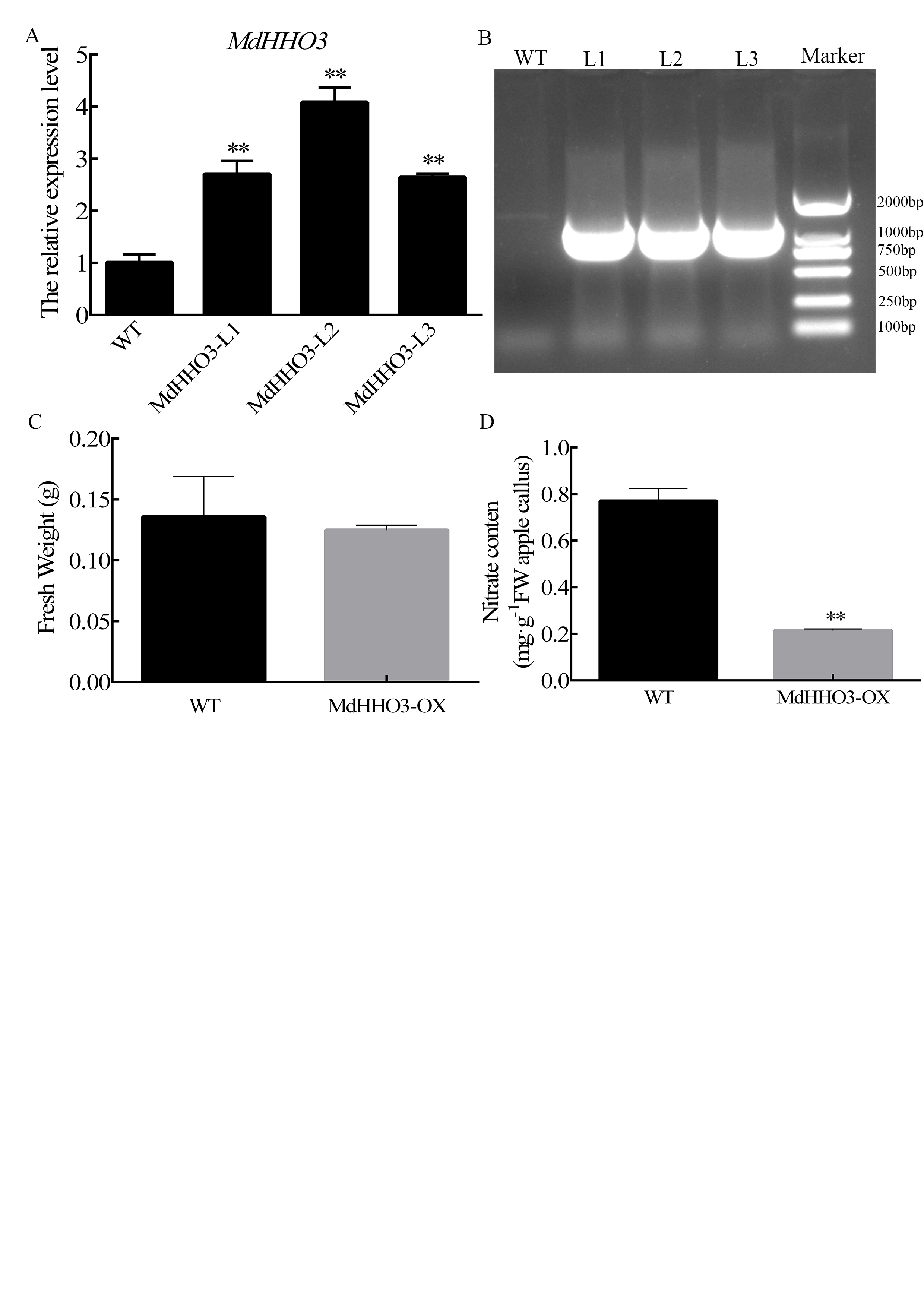


**Figure S1 Overexpression of *MdHHO3* in apple calli.** (A) Expression level of *MdHHO3* in WT, MdHHO3-L1, MdHHO3-L2, and MdHHO3-L3. (B) The PCR results verified the overexpression of MdHHO3 in apple calli. The data are expressed as the means ± SDs (n = 3). Significant differences were detected by a t-test: * P < 0.05 and ** P < 0.01.


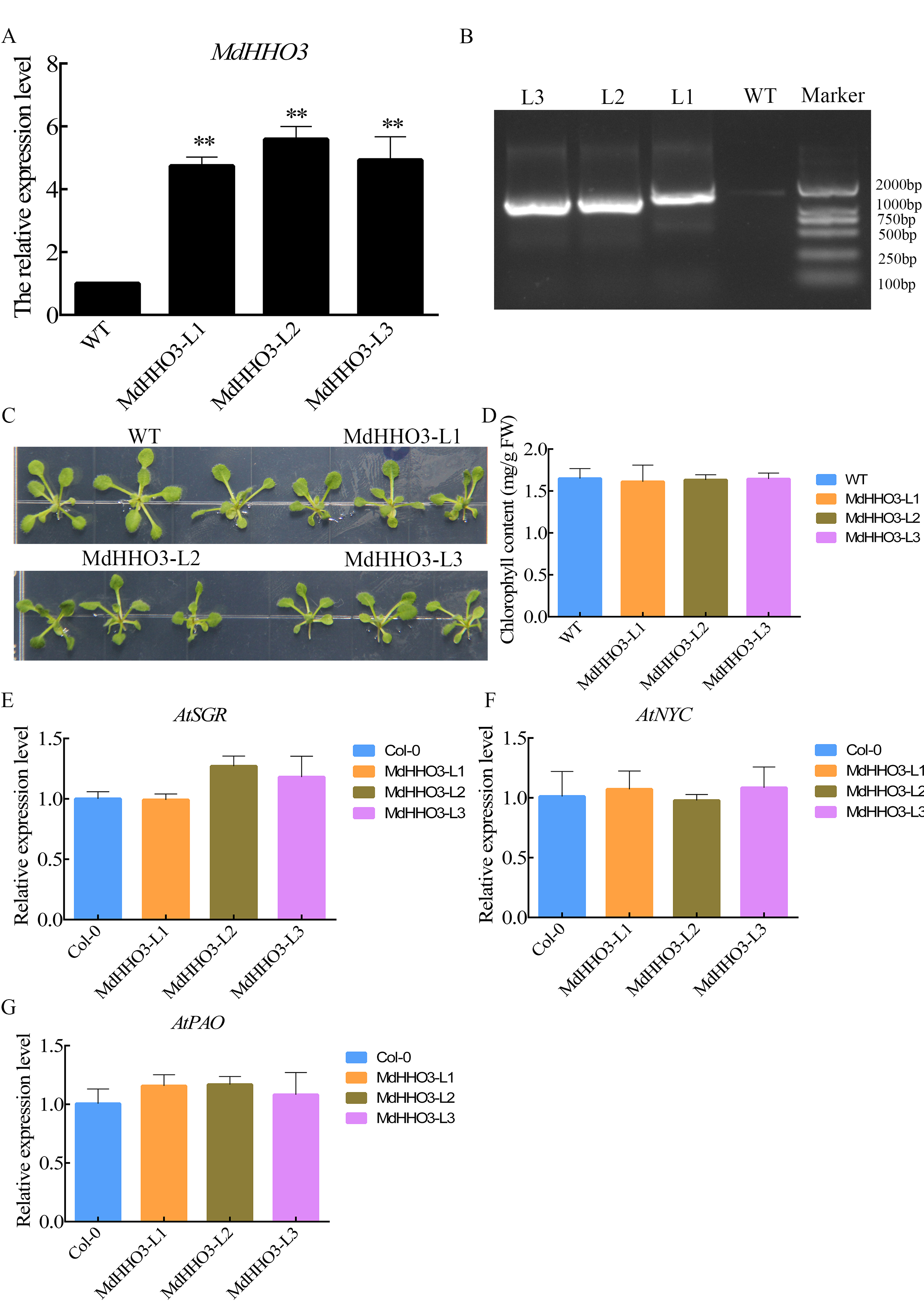


**Figure S2 Overexpression of *MdHHO3* in *Arabidopsis thaliana.*** (A) Expression level of MdHHO3 in three transgenic Arabidopsis lines. (B) The PCR results verified the overexpression of *MdHHO3* in *Arabidopsis thaliana*. (C-E) Expression of senescence-related genes (*AtSGR*, *AtNYC* and *AtPAO*) in untreated *Arabidopsis thaliana*.


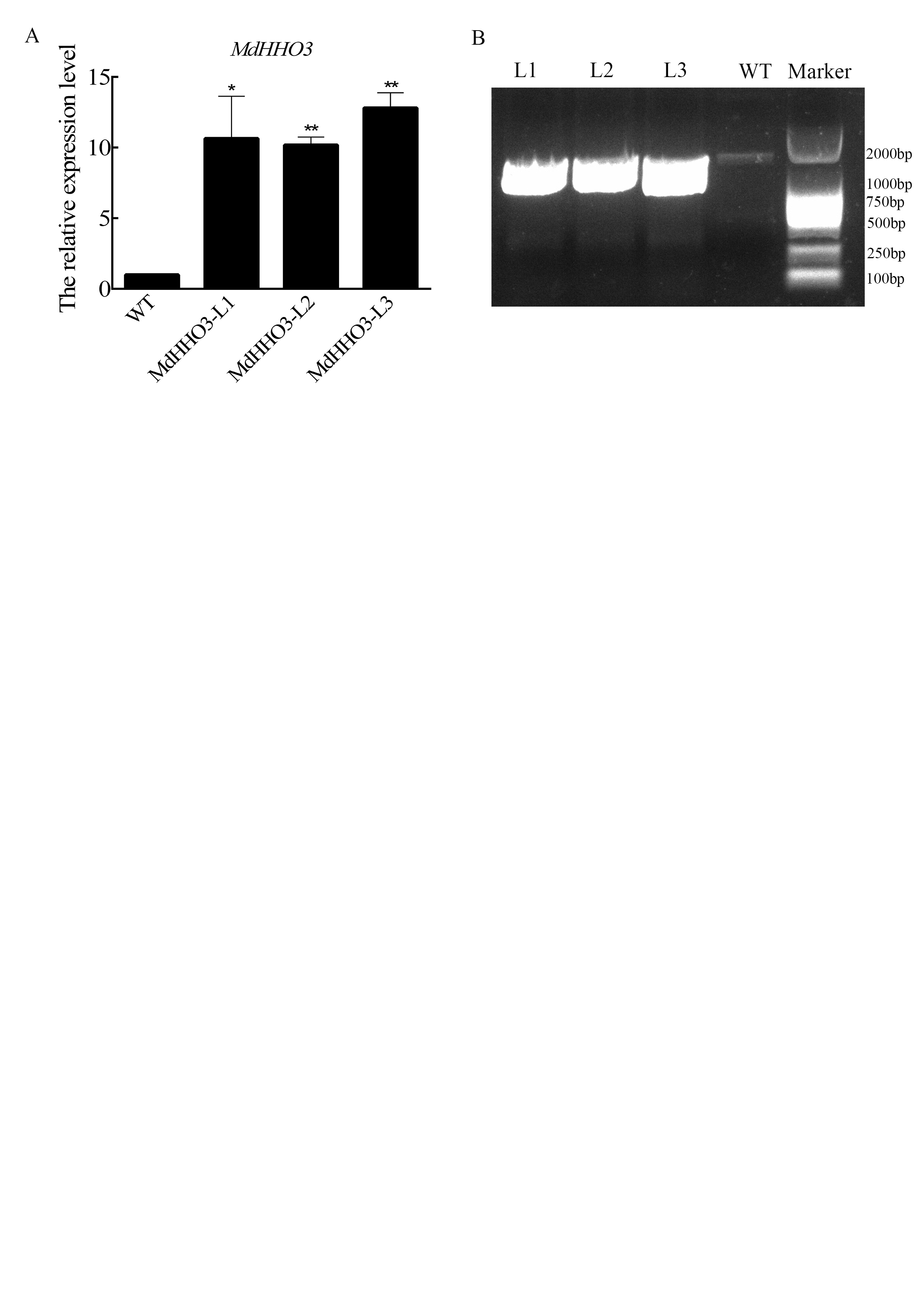


**Figure S3 Overexpression of *MdHHO3* in tobacco*.*** (A) Expression level of *MdHHO3* in three transgenic tobacco lines. (B) The PCR results verified the overexpression of *MdHHO3* in tobacco.


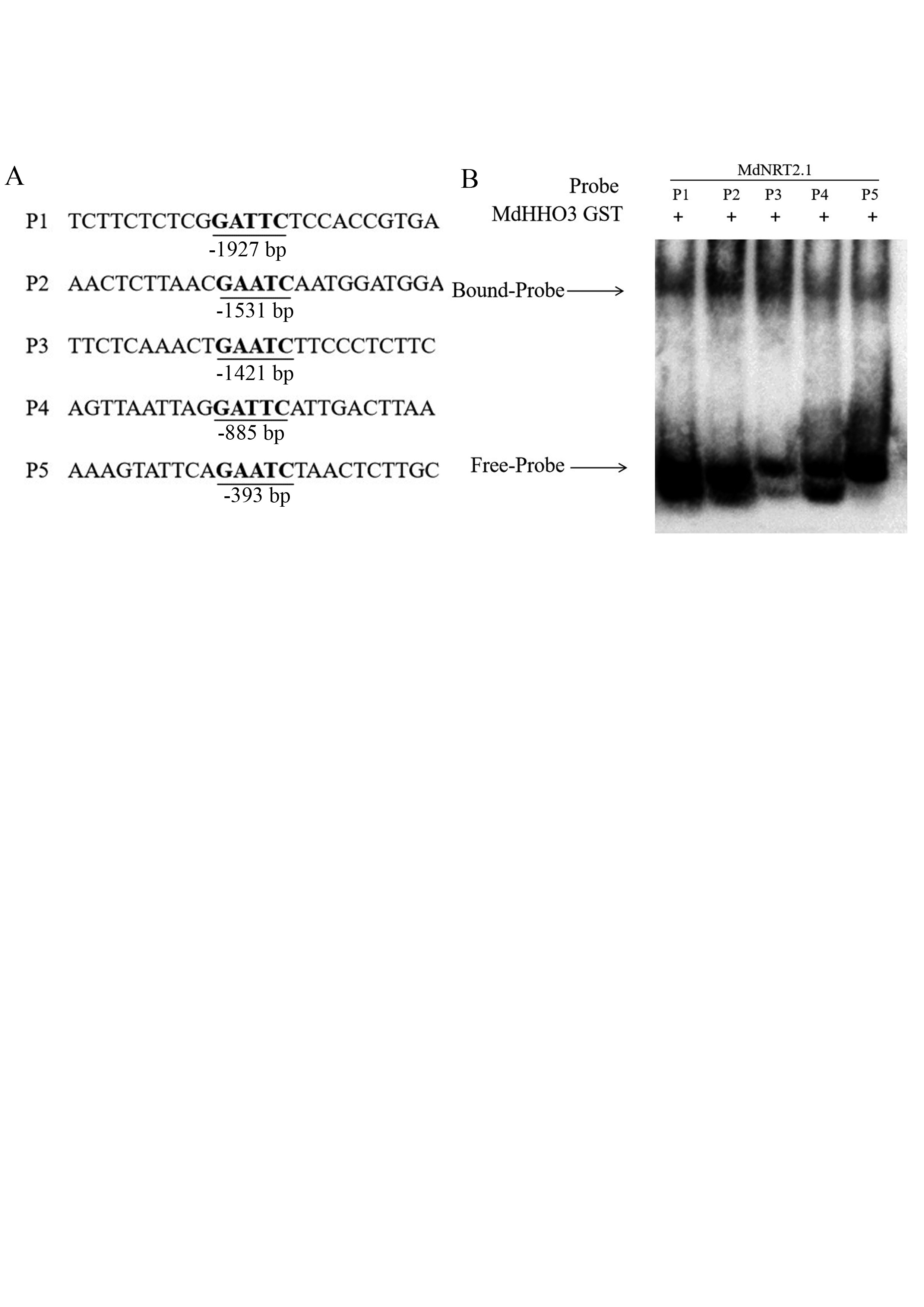


**Figure S4** **MdHHO3 binds to a specific sequence of the MdNRT2.1 promoter.** (A) GAATC and GATTC motifs in the promoter region of MdNRT2.1 selected in the DNA-binding site. (B) The electrophoretic mobility shift assay (EMSA) results showed that the MdHHO3-GST fusion protein binds to the labeled DNA probe in the MdNRT2.1 promoter. The MdHHO3-GST fusion protein was incubated with a labeled probe DNA fragment. The labeled probe used in this study is P1-P5. The + symbol indicates presence.

**
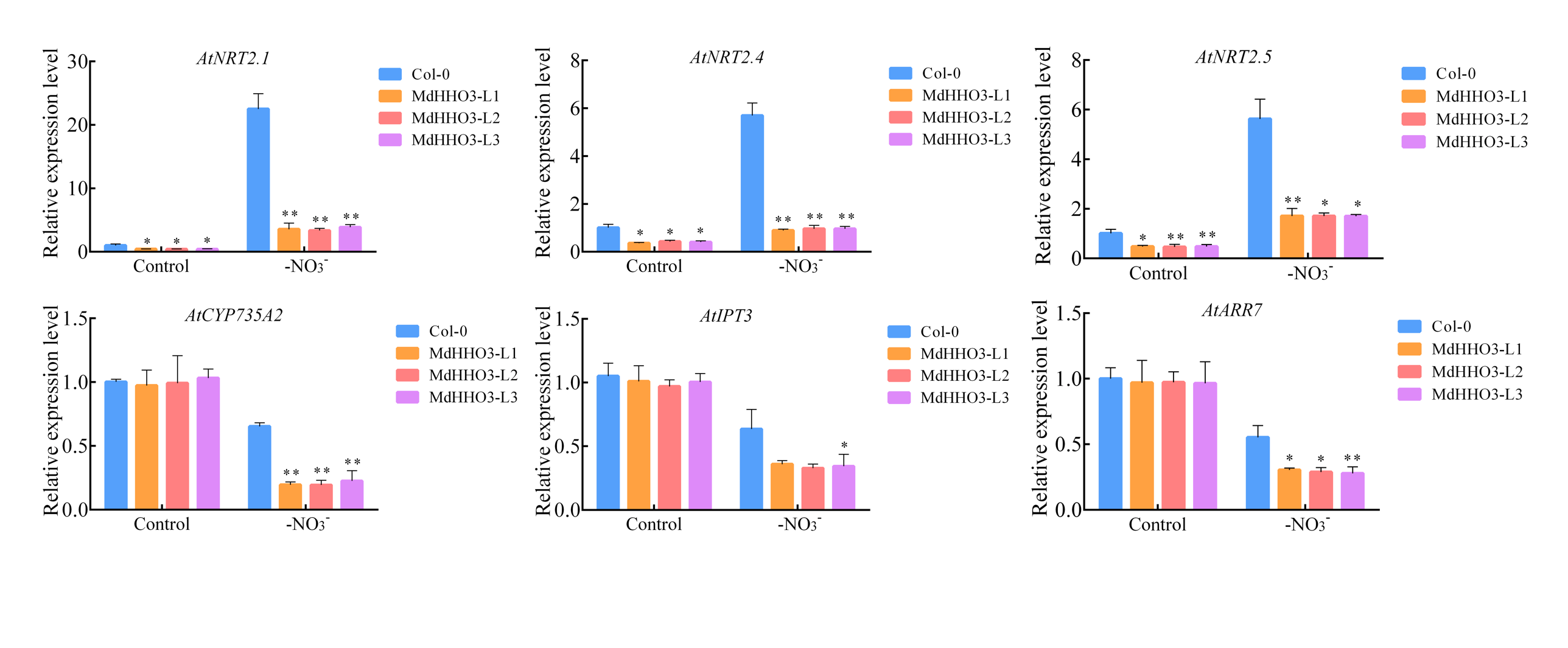
**

**Figure S5** **Expression of high-affinity nitrate transporter genes (*AtSGR1*, *AtNYC1* and *AtPAO*) and cytokinin synthesis enzyme-related genes (*AtCYP735A2*, *AtIPT3*, *AtARR7*) in *Arabidopsis* after exposure to nitrate deficiency for 4 days.** Three biological replicates of each sample were included, and the data are expressed as the means ± SDs (n = 3). Significant differences were detected by a t-test: * P < 0.05 and ** P < 0.01.


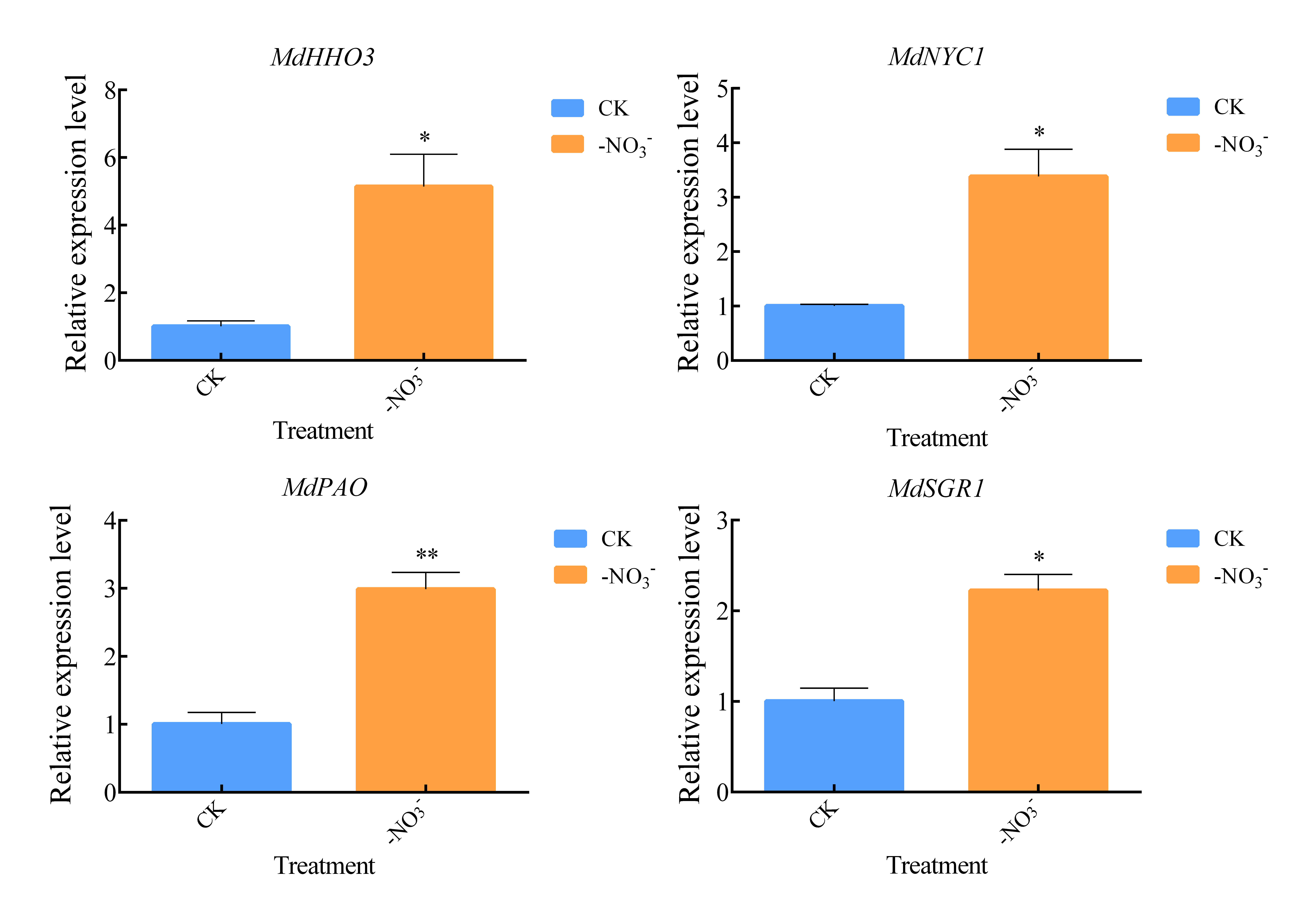


**Figure S6 Expression patterns of *MdHHO3* and chlorophyll catabolism related genes (*MdNYC1*, *MdPAO*, *MdSGR1*) during nitrate deficiency.** Three biological replicates of each sample were included, and the data are expressed as the means ± SDs (n = 3). Significant differences were detected by a t-test: * P < 0.05 and ** P < 0.01.

**Table S1 Sequences used for phylogenetic tree analysis**

>AT1G49190.1 AtGARP1

MLVGKISGYEDNTRSLERETSEITSLLSQFPGNTNVLVVDTNFTTLLNMKQIMKQYAYQV

SIETDAEKALAFLTSCKHEINIVIWDFHMPGIDGLQALKSITSKLDLPVVIMSDDNQTES

VMKATFYGACDYVVKPVKEEVMANIWQHIVRKRLIFKPDVAPPVQSDPARSDRLDQVKADFKIVEDEPIINETPLITWTEEIQPVQSDLVQANKFDQVNGYSPIMNQDNMFNKAPPKPRM

TWTEVIQPVQSNLVQTKEFGQLNDYSQIMNQDSMYNKAATKPQLTWTEEIQPVQSGLVQANEFSKVNGYSQSMNQDSMFNKSATNPRLTWNELLQPVQSDLVQSNEFSQFSDYSQIMNEDNMFNKAAKKPRMTWSEVFQPVQSHLVPTDGLDRDHFDSITINGGNGIQNMEKKQGKKPRKPRMTWTEELHQKFLEAIEIIGANPKVLVECLQEMRIEGITRSNVASHLQKHRINLEENQI

PQQTQGNGWATAYGTLAPSLQGSDNVNTTIPSYLMNGPATLNQIQQNQYQNGFLTMNNNQIITNPPPPLPYLDHHHQQQHQSSPQFNYLMNNEELLQASGLSATDLELTYPSLPYDPQEY

LINGYNYN

>AT1G67710.1 AtGARP2

MEKSGFSPVGLRVLVVDDDPTWLKILEKMLKKCSYEVTTCGLAREALRLLRERKDGYDIVISDVNMPDMDGFKLLEHVGLELDLPVIMMSVDGETSRVMKGVQHGACDYLLKPIRMKELKIIWQHVLRKKLQEVRDIEGCGYEGGADWITRYDEAHFLGGGEDVSFGKKRKDFDFEKKLLQDESDPSSSSSKKARVVWSFELHHKFVNAVNQIGCDHKAGPKKILDLMNVPWLTRENVASHLQKYRLYLSRLEKGKELKCYSGGVKNADSSPKDVEVNSGYQSPGRSSYVFSGGNSLIQKATEIDPKPLASASLSDLNTDVIMPPKTKKTRIGFDPPISSSAFDSLLPWNDVPEVLESKPVLYENSFLQQQPLPSQSSYVANSAPSLMEEEMKPPYETPAGGSSVNADEFLMPQDKIPTVTLQDLDPSAMKLQEFNTEAILRSLNWELPESHHSVSLDTDLDLTWLQGERFLANTGLQFQDYSSSPSLLSELPAHLNWYGNERLPDPDEYSFMVDQGLFIS

>AT1G68210.1 AtGARP3

MAGCLVPECADDISILLIDHDTASIASLTSMLQQFSKRVMSVDVASKALSMIEKQKKEIG

LIIANIEMPHIDSHSFLNALLLKDIPLILINPEIKTKEPSDLLTKRACFSLDKPISNDDI

KNMWQHVFSKKSQELKKINITEDQENVMDKDTYQIEAFRANLKRQRISQASLLGRRPFID

TFSTYETFQKRKSIANVEWKTTPSYAIEIENKRKEWKKSVGRRKSLWNSERHMKFIAAIS

ILGEEDFRPKSILEIMNDPNLTHRQVGSHLQKYKAQIDQISYTLPRNESRSIDKTFEYPS

NYKYPFKISDLTNNLIVSNSLWNSLEKKNSASASITQFLFKKPIGEKEETMPKFHIGGKL

DLSNHSVHGNVLNKLSMNVNFVPSTISNNPAYNILSIDSSSIDSSSYTGLVSTGLSSENS

PILYGLPSNDGASNTCTSQMESERISIPQYDPNQCHPHRSILETDVNQIDLDFTSILDSF

DPLVDECLMKENNRFLPNPTMNLLDTDIDKMDWVSFIENLSHHDINMNHMDWDPSTANYVLPETNMNINFPEKHTNEIGWVSSQVGYVPFENMIPSEVDINHMGMGYSGGSIPPQEETNTNNVGFVSCEIHSEIPPKTNMAILETNYSNPLDWVFPEDITSLETNTIQKSLVSCETSYDA

LDNMVPLETNMEEMNDALYDISIEDLISFDIDANEKDIFSWLEDNGFSEENNMMESCEYH

NIESVNQSDDMKIDDNFDDYRECMDWINEEMNKDV

>AT2G01760.1 AtGARP4

MPINDQFPSGLRILVVDDDTSCLFILEKMLLRLMYQVTICSQADVALTILRERKDSFDLV

LSDVHMPGMNGYNLLQQVGLLEMDLPVIMMSVDGRTTTVMTGINHGACDYLIKPIRPEELKNIWQHVVRRKCVMKKELRSSQALEDNKNSGSLETVVVSVSECSEESLMKCRNKKKKKKRSVDRDDNEDDLLLDPGNSKKSRVVWSIELHQQFVNAVNKLGIDKAVPKRILELMNVPGLSRENVASHLQKFRLYLKRLSGEASQSNDSESTKRYENIQALVSSGQLHPQTLAALFGQPIDNHHSASFGVWIPNDNLGRSQNEHFSVDVSSASNRPVSVAVHGLSSSANFRQRGDVNNNRIRQGYGSNVNEESWILERSSRQR

>AT2G25180.1 AtGARP5

MTVEQNLEALDQFPVGMRVLAVDDDQTCLKILESLLRHCQYHVTTTNQAQKALELLRENKNKFDLVISDVDMPDMDGFKLLELVGLEMDLPVIMLSAHSDPKYVMKGVTHGACDYLLKPVRIEELKNIWQHVVRSRFDKNRGSNNNGDKRDGSGNEGVGNSDQNNGKGNRKRKDQYNEDEDEDRDDNDDSCAQKKQRVVWTVELHKKFVAAVNQLGYEKAMPKKILDLMNVEKLTRENVASHLQKFRLYLKRISGVANQQAIMANSELHFMQMNGLDGFHHRPIPVGSGQYHGGAPAMRSFPPNGILGRLNTPSGIGVRSLSSPPAGMFLQNQTDIGKFHHVSSLPLNHSDGGNILQGLPMPLEFDQLQTNNNKSRNMNSNKSIAGTSMAFPSFSTQQNSLISAPNNNVVVLEGHPQATPPGFPGHQINKRLEHWSNAVSSSTHPPPPAHNSNSINHQFDVSPLPHSRPDPLEWNNVSSSYSIPFCDSANTLSSPALDTTNPRAFCRNTDFDSNTNVQPGVFYGPSTDAMALLSSSNPKEGFVVGQQKLQSGGFMVADAGSLDDIVNSTMKQEQSQGDLSGGDLGYGGFSSLRTCI

>AT2G27070.1 AtGARP6

MAFAQSVYNQSSVLKINVMVVDDNRVFLDIWSRMLEKSKYREITVIAVDYPKKALSTLKN

QRDNIDLIITDYYMPGMNGLQLKKQITQEFGNLSVLVMSSDPNKEEESLSCGAMGFIPKP

IAPTDLPKIYQFALTYKRNGKSTLSTEQNQKDANVSVPQQIMLVPEQAYVLKTKKKNCSS

KSDTRTVNSTNVSHVSTNGSRKNRKRKPKGGPSDDGESLSQPPKKKKIWWTNPLQDLFLQAIQHIGYDKVVPKKILAIMNVPYLTRENVASHLQKYRLFVKRVVHQGRFSMLSDRGKDSMFRQTHIKEPYVNYYTPSTSWYETSLNNRSFYSESVHGHSRLLSEAREPVRYNQMSYNYMNRNISFENQPSQNEETRTVFEPPVMANKISQTSQVLGFGQLGPSAISGHNFNTNMMSSYGSLTPNQPGTSHFSYGMQSVLNNENATYNPQPPANATTQPNLDELPQLENLNLYNDLGNTSELPYNISNFQSDDNKKQGEEDGDWTFVNINQDQSNGESSNTIATPETNTPNFNINPNQNQGQAVPEFTDWSFLDQQPRRLGIRIGYFGFKFSGLEV

>AT3G16857.1 AtGARP7

MMNPSHGRGLGSAGGSSSGRNQGGGGETVVEMFPSGLRVLVVDDDPTCLMILERMLRTCLYEVTKCNRAEMALSLLRKNKHGFDIVISDVHMPDMDGFKLLEHVGLEMDLPVIMMSADDSKSVVLKGVTHGAVDYLIKPVRMEALKNIWQHVVRKRRSEWSVPEHSGSIEETGERQQQQHRGGGGGAAVSGGEDAVDDNSSSVNEGNNWRSSSRKRKDEEGEEQGDDKDEDASNLKKPRVVWSVELHQQFVAAVNQLGVEKAVPKKILELMNVPGLTRENVASHLQKYRIYLRRLGGVSQHQGNLNNSFMTGQDASFGPLSTLNGFDLQALAVTGQLPAQSLAQLQAAGLGRPAMVSKSGLPVSSIVDERSIFSFDNTKTRFGEGLGHHGQQPQQQPQMNLLHGVPTGLQQQLPMGNRMSIQQQIAAVRAGNSVQNNGMLMPLAGQQSLPRGPPPMLTSSQSSIRQPMLSNRISERSGFSGRNNIPESSRVLPTSYTNLTTQHSSSSMPYNNFQPELPVNSFPLASAPGISVPVRKATSYQEEVNSSEAGFTTPSYDMFTTRQNDWDLRNIGIAFDSHQDSESAAFSASEAYSSSSMSRHNTTVAATEHGRNHQQPPSGMVQHHQVYADGNGGSVRVKSERVATDTATMAFHEQYSNQEDLMSALLKQV

>AT3G62670.1 AtGARP8

MAFLMKNKHEIDLVIWDFHMPDINGLDALNIIGKQMDLPVVIMSHEYKKETVMESIKYGACDFLVKPVSKEVIAVLWRHVYRKRMSKSGLDKPGESGTVESDPDEYDDLEQDNLYESNEEGSKNTCDHKEEKSPTKKPRMQWTPELHHKFEVAVEKMGSLEKAFPKTILKYMQEELNVQGLTRNNVASHLQKYRQSSKKTCTPQEPQEDFVWGNAGPDVTLAASKTLLSSHATPSYLINNQAAPRGSYFMNNIPYPSTSCLPVNNNNCFMTNPSTYIDQFQHQLQQQQQHQQYQSTLNSISAMLTKQESRHVPSSAMENSEPLMIYNSNLPFGIDECFPPAGFNIFDQIGHN

>AT4G16110.1 AtGARP9

MVNPGHGRGPDSGTAAGGSNSDPFPANLRVLVVDDDPTCLMILERMLMTCLYRVTKCNRAESALSLLRKNKNGFDIVISDVHMPDMDGFKLLEHVGLEMDLPVIMMSADDSKSVVLKGVTHGAVDYLIKPVRIEALKNIWQHVVRKKRNEWNVSEHSGGSIEDTGGDRDRQQQHREDADNNSSSVNEGNGRSSRKRKEEEVDDQGDDKEDSSSLKKPRVVWSVELHQQFVAAVNQLGVDKAVPKKILEMMNVPGLTRENVASHLQKYRIYLRRLGGVSQHQGNMNHSFMTGQDQSFGPLSSLNGFDLQSLAVTGQLPPQSLAQLQAAGLGRPTLAKPGMSVSPLVDQRSIFNFENPKIRFGDGHGQTMNNGNLLHGVPTGSHMRLRPGQNVQSSGMMLPVADQLPRGGPSMLPSLGQQPILSSSVSRRSDLTGALAVRNSIPETNSRVLPTTHSVFNNFPADLPRSSFPLASAPGISVPVSVSYQEEVNSSDAKGGSSAATAGFGNPSYDIFNDFPQHQQHNKNISNKLNDWDLRNMGLVFSSNQDAATATATAAFSTSEAYSSSSTQRKRRETDATVVGEHGQNLQSPSRNLYHLNHVFMDGGSVRVKSERVAETVTCPPANTLFHEQYNQEDLMSAFLKQEGIPSVDNEFEFDGYSIDNIQV

>AT4G18020.1 AtGARP10

MVITANDLSKWENFPKGLKVLLLLNGCDSDGDGSSAAETRSELESMDYIVTTFTDETEAL

SAVVKNPESFHIAIVEVNMSAESESFKFLEAAKDVLPTIMISTDHCITTTMKCIALGAVE

FLQKPLSPEKLKNIWQHVVHKAFNDGGSNVSISLKPVKESVVSMLHLETDMTIEEKDPAP

STPQLKQDSRLLDGDCQENINFSMENVNSSTEKDNMEDHQDIGESKSVDTTNRKLDDDKVVVKEERGDSEKEEEGETGDLISEKTDSVDIHKKEDETKPINKSSGIKNVSGNKTSRKKVD

WTPELHKKFVQAVEQLGVDQAIPSRILELMKVGTLTRHNVASHLQKFRQHRKNILPKDDH

NHRWIQSRENHRPNQRNYNVFQQQHRPVMAYPVWGLPGVYPPGAIPPLWPPPLQSIGQPP

PWHWKPPYPTVSGNAWGCPVGPPVTGSYITPSNTTAGGFQYPNGAETGFKIMPASQPDEE

MLDQVVKEAISKPWLPLPLGLKPPSAESVLAELTRQGISAVPSSSCLINGSHRLR

>AT4G31920.1 AtGARP11

MTMEQEIEVLDQFPVGMRVLAVDDDQTCLRILQTLLQRCQYHVTTTNQAQTALELLRENKNKFDLVISDVDMPDMDGFKLLELVGLEMDLPVIMLSAHSDPKYVMKGVKHGACDYLLKPVRIEELKNIWQHVVRKSKLKKNKSNVSNGSGNCDKANRKRKEQYEEEEEEERGNDNDDPTAQKKPRVLWTHELHNKFLAAVDHLGVERAVPKKILDLMNVDKLTRENVASHLQKFRVALKKVSDDAIQQANRAAIDSHFMQMNSQKGLGGFYHHHRGIPVGSGQFHGGTTMMRHYSSNRNLGRLNSLGAGMFQPVSSSFPRNHNDGGNILQGLPLEELQINNNINRAFPSFTSQQNSPMVAPSNLLLLEGNPQSSSLPSNPGFSPHFEISKRLEHWSNAALSTNIPQSDVHSKPDTLEWNAFCDSASPLVNPNLDTNPASLCRNTGFGSTNAAQTDFFYPLQMNQQPANNSGPVTEAQLFRSSNPNEGLLMGQQKLQSGLMASDAGSLDDIVNSLMTQEQSQSDFSEGDWDLDGLAHSEHAYEKLHFPFSLSA

>AT5G07210.1 AtGARP12

MASAQSFYNQSSVLKINVMVVDDDHVFLDIMSRMLQHSKYRDPSVMEIAVIAVDDPKKALSTLKIQRDNIDLIITDYYMPGMNGLQLKKQITQEFGNLPVLVMSSDTNKEEESLSCGAMG

FIPKPIHPTDLTKIYQFALSNKRNGKSTLSTEQNHKDADVSVPQQITLVPEQADVLKTKR

KNCSFKSDSRTVNSTNGSCVSTDGSRKNRKRKPNGGPSDDGESMSQPAKKKKIQWTDSLHDLFLQAIRHIGLDKAVPKKILAFMSVPYLTRENVASHLQKYRIFLRRVAEQGLYSMLSDR

GIDSMFRQTHIKEPYFNYYTPSTSWYDTRLNNRSFYSKPVHGFGQSKLLSTTREPVCFNQ

MPYNYMNRSSTYEPHRIGSGSNLTLPIQSNLSFPNQPSQNEERRSFFEPPVMANKIAQTS

QVLGFGQLGPSAISGHNFNNNMTSRYGSLIPSQPGPSHFSYGMQSFLNNENVTYNPQPPA

NATTQPNLDELPQLENLNLYNDFGNTSELPYNISNFQFDDNKHQQGEADPTKFELPAAKF

STELNHEDDGDWTFVNINQGQSNGETSNTIASPETNTPILNINHNQNQGQDVPEFNDWSF

LDPQELVDDDFMNSLFNNDMN

>AT5G49240.1 AtGARP13

MQPLNMAEILDHRGVLTDGDDGPFRNLTNFYDMFSSNFPEGLRVLVFDEDPSYLLILERH

LQKFQYQVTICNEVNKAMHTLRNHRNRFDLAMIQVNNAEGDIFRFLSEIGSEMDLPIIII

SEDDSVKSVKKWMINGAADYLIKPIRPEDLRIVFKHLVKKMRERRSVVTGEAEKAAGEKS

SSVGDSTIRNPNKSKRSSCLEAEVNEEDRHDHNDRACASSAKKRRVVWDEELHQNFLNAVDFLGLERAVPKKILDVMKVDYISRENVASHLQVTFLIYNIIVHFQQHFCFYS

>AT5G58080.1 AtGARP14

MRVLAVDDNPTCLRKLEELLLRCKYHVTKTMESRKALEMLRENSNMFDLVISDVEMPDTDGFKLLEIGLEMDLPVIMLSAHSDYDSVMKGIIHGACDYLVKPVGLKELQNIWHHVVKKNIKSYAKLLPPSESDSVPSASRKRKDKVNDSGDEDDSDREEDDGEGSEQDGDGSGTRKKPRVVWSQELHQKFVSAVQQLGLDKAVPKKILDLMSIEGLTRENVASHLQKYRLYLKKIDEGQQQNMTPDAFGTRDSSYFQMAQLDGLRDFTAARQIPSSGLLSRSHLTKLQPPMYSSINLQGMNSSSFIQQGHHQNSSNSANPFGTYHSTLSPRIQNVNLFQRTSSPLEPLQFPRSKSYIGDF

KGLGDRAIGGSFLDTCMPFGSSSTSLPSASTNPLMLQANYTQPLHIASDGIQPCIEGTPS

NSASPNISFQGLSRFPGHSWQGNLNTTRFPPSSLPLNLAFLPDQVTCAGNNLGDCTSLVS

AENPGGEMQCDPQLLGGFMQNVNPLGGQKWEQQNCTMLNNPFGNIEYPLPADNMVFRDNNSTRSKGLDESLMNPIDNSQEYVGKATTMLDPEMKSGKPENDNQHDVFDDIMNEMMKQEENNGMVPVATRFGFDSFPPP

>AT1G13300.1 AtGARP15

MIKKFSNMDYNQKRERCGQYIEALEEERRKIHVFQRELPLCLDLVTQAIEACKRELPEMT

TENMYGQPECSEQTTGECGPVLEQFLTIKDSSTSNEEEDEEFDDEHGNHDPDNDSEDKNT

KSDWLKSVQLWNQPDHPLLPKEERLQQETMTRDESMRKDPMVNGGEGRKREAEKDGGGGRKQRRCWSSQLHRRFLNALQHLGGPHVATPKQIREFMKVDGLTNDEVKSHLQKYRLHTRRPRQTVPNNGNSQTQHFVVVGGLWVPQSDYSTGKTTGGATTSSTTTTTGIYGTMAAPPPPQWPSHSNYRPSIIVDEGSGSHSEGVVVRCSSPAMSSSTRNHYVKNN

>AT1G14600.1 AtGARP16

MGRCGRSNDGVIGGVRPYVRSPVPRLRWTPELHRSFVHAVDLLGGQYKATPKLVLKIMDVKGLTISHVKSHLQMYRGSRITLLGKPEESSSPSSRRRRRQDNEEDHLHDNLSVHARNDCLLGFHSFNFREQTSATDNDDDDFLNIMNMERTKTFAGNGESIKFQSHHSLEAENTKNIWKNTWRENEHEEEEELSLSLSLNHPHNHQQRWKSNASSSLSETSEAVSSSSGPFIFRDCFASS

KIDLNLNLSFSLLHS

>AT1G25550.1 AtGARP17

MMMFKSGDMDYTQKMKRCHEYVEALEEEQKKIQVFQRELPLCLELVTQAIESCRKELSESSEHVGGQSECSERTTSECGGAVFEEFMPIKWSSASSDETDKDEEAEKTEMMTNENNDGDKKKSDWLRSVQLWNQSPDPQPNNKKPMVIEVKRSAGAFQPFQKEKPKAADSQPLIKAITPTSTTTTSSTAETVGGGKEFEEQKQSHSNRKQRRCWSPELHRRFLHALQQLGGSHVATPKQI

RDLMKVDGLTNDEVKSHLQKYRLHTRRPATPVVRTGGENPQQRQFMVMEGIWVPSHDTTNNRVYAPVATQPPQSSTSGERSNRGCKSPATSSTTTHTPHLLPLS

>AT1G32240.1 AtGARP18

MELFPAQPDLSLQISPPNSKPSSTWQRRRSTTDQEDHEELDLGFWRRALDSRTSSLVSNS

TSKTINHPFQDLSLSNISHHQQQQQHHHPQLLPNCNSSNILTSFQFPTQQQQQHLQGFLA

HDLNTHLRPIRGIPLYHNPPPHHHPHRPPPPCFPFDPSSLIPSSSTSSPALTGNNNSFNT

SSVSNPNYHNHHHQTLNRARFMPRFPAKRSMRAPRMRWTTTLHARFVHAVELLGGHERATPKSVLELMDVKDLTLAHVKSHLQMYRTVKTTDKAAASSGQSDVYENGSSGDNNSDDWMFDMNRKSRDSEELTNPLEKSNGLWTNSSGEARLHGKLIDNVAEIMLPSEKELDGKCSSYERISSEEMSSSSISGTSPFKPNLEFTLGRSH

>AT1G49560.1 AtGARP19

MGSLGDELSLGSIFGRGVSMNVVAVEKVDEHVKKLEEEKRKLESCQLELPLSLQILNDAI

LYLKDKRCSEMETQPLLKDFISVNKPIQGERGIELLKREELMREKKFQQWKANDDHTSKI

KSKLEIKRNEEKSPMLLIPKVETGLGLGLSSSSIRRKGIVASCGFTSNSMPQPPTPAVPQ

QPAFLKQQALRKQRRCWNPELHRRFVDALQQLGGPGVATPKQIREHMQEEGLTNDEVKSHLQKYRLHIRKPNSNAEKQSAVVLGFNLWNSSAQDEEETCEGGESLKRSNAQSDSPQGPLQLPSTTTTTGGDSSMEDVEDAKSESFQLERLRSP

>AT1G68670.1 AtGARP20

MMVEMDYAKKMQKCHEYVEALEEEQKKIQVFQRELPLCLELVTQAIEACRKELSGTTTTTSEQCSEQTTSVCGGPVFEEFIPIKKISSLCEEVQEEEEEDGEHESSPELVNNKKSDWLRS

VQLWNHSPDLNPKEERVAKKAKVVEVKPKSGAFQPFQKRVLETDLQPAVKVASSMPATTT

SSTTETCGGKSDLIKAGDEERRIEQQQSQSHTHRKQRRCWSPELHRRFLNALQQLGGSHV

ATPKQIRDHMKVDGLTNDEVKSHLQKYRLHTRRPAATSVAAQSTGNQQQPQFVVVGGIWVPSSQDFPPPSDVANKGGVYAPVAVAQSPKRSLERSCNSPAASSSTNTNTSTPVS

>AT1G69580.1 AtGARP21

MCLLMEINNNANNTNTTIDNHKAKMSLVLSTDAKPRLKWTCDLHHKFIEAVNQLGGPNKATPKGLMKVMEIPGLTLYHLKSHLQKYRLGKSMKFDDNKLEVSSASENQEVESKNDSRDLRGCSVTEENSNPAKEGLQITEALQMQMEVQKKLHEQIEVQRHLQVKIEAQGKYLQSVLMKAQQTLAGYSSSNLGMDFARTELSRLASMVNRGCPSTSFSELTQVEEEEEGFLWYKKPENRGISQLRCSVESSLTSSETSETKLDTDNNLNKSIELPLMEINSEVMKGKKRSINDVVCVEQPLMKRAFGVDDDEHLKLSLNTYKKDMEACTNIGLGFN

>AT1G79430.1 AtGARP22

MRVMGVKGLTLYHLKSHLQKFRLGKQPHKEYGDHSTKEGSRASAMDIQRNVASSSGMMSRNMNEMQMEVQRRLHEQLEVQRHLQLRIEAQGKYMQSILERACQTLAGENMAAATAAAAVGGGYKGNLGSSSLSAAVGPPPHPLSFPPFQDLNIYGNTTDQVLDHHNFHHQNIENHFTGNNAADTNIYLGKKRPNPNFGNDVRKGLLMWSDQDHDLSANQSIDDEHRIQIQMATHVSTDLDSLSEIYERKSGLSGDEGNNGGKLLERPSPRRSPLSPMMNPNGGLIQGRNSPFG

>AT2G01060.1 AtGARP23

MEADNGGPNSSHASKQRLRWTHELHERFVDAVAQLGGPDRATPKGVLRVMGVQGLTIYHVKSHLQKYRLAKYLPDSSSEGKKTDKKESGDMLSGLDGSSGMQITEALKLQMEVQKRLHEQLEVQRQLQLRIEAQGKYLKKIIEEQQRLSGVLGEPSAPVTGDSDPATPAPTSESPLQDKSGKDCGPDKSLSVDESLSSYREPLTPDSGCNIGSPDESTGEERLSKKPRLVRGAAGYTPDI

VVGHPILESGLNTSYHQSDHVLAFDQPSTSLLGAEEQLDKVSGDNL

>AT2G02060.1 AtGARP24

MGKSSGRNGNGSFNGNKFHGVRPYVRSPVPRLRWTPDLHRCFVHAVEILGGQHRATPKLVLKMMDVKGLTISHVKSHLQMYRGGSKLTLEKPEESSSSSIRRRQDSEEDYYLHDNLSLHTRNDCLLGFHSFPLSSHSSFRGGGGGRTKEQQTSESGGYDDDADFLHIKKMNDTTTFLSHHFPKGTEEWREQEHEEEEEDLSLSLSLNHHHWRSNGSSVVSETSEAAVSTCSAPFVSKDCF

GSSKIDLNLSISLLGS

>AT2G03500.1 AtGARP25

MASSSELSLDCKPQSYSMLLKSFGDNFQSDPTTHKLEDLLSRLEQERLKIDAFKRELPLC

MQLLNNAVEVYKQQLEAYRANSNNNNQSVGTRPVLEEFIPLRNQPEKTNNKGSNWMTTAQLWSQSETKPKNIDSTTDQSLPKDEINSSPKLGHFDAKQRNGSGAFLPFSKEQSLPELALS

TEVKRVSPTNEHTNGQDGNDESMINNDNNYNNNNNNNSNSNGVSSTTSQSNRKARRCWSPDLHRRFVQALQMLGGSQVATPKQIRELMKVDGLTNDEVKSHLQKYRLHTRRPSPSPQTSGGPGPHLVVLGGIWVPPEYTSAHGGTPTLYHHQVHHHHTNTAGPPPPHFCSSQEFYTTPPPPQPLHHHHFQTFNGSSGGTASTDSTHHQVTDSPTVEGKSPESGGGERKGLAALREECEDHSNINGSEITLKF

>AT2G06020.1 AtGARP26

MKKQEKGNEVVTFEENPSTYVHHHTKKASMVLSEQEIEVILQSNKETYNEKLEKNTKENAEKVGIEEGNGSSSKITPCIFYTSDEKARLRWSSDLHDCFVNAVEKLGGPNKATPKSVKEA

MEVEGIALHHVKSHLQKFRLGKCNIRGGTNQYVRQSQSMPHKSQQQLQLNNDCVNHDSYCNATFLSVTVPPTIDSLHQDTLNNTILFPTTLQAIPTSVSHNTYMSSYPSYNTVEVVKAQL

NALQDSSTSQTQETFSRETNLSSIVTRDEADPVDKYIDWNKVGETDIEHDSVEVLEALGL

K

>AT2G20400.1 AtGARP27

MIPNDDDDANSMKNYPLNDDDANSMKNYPLNDDDANSMENYPLRSIPTELSHTCSLIPPS

LPNPSEAAADMSFNSELNQIMARPCDMLPANGGAVGHNPFLEPGFNCPETTDWIPSPLPH

IYFPSGSPNLIMEDGVIDEIHKQSDLPLWYDDLITTDEDPLMSSILGDLLLDTNFNSASK

VQQPSMQSQIQQPQAVLQQPSSCVELRPLDRTVSSNSNNNSNSNNAAAAAKGRMRWTPELHEVFVDAVNQLGGSNEATPKGVLKHMKVEGLTIFHVKSHLQKYRTAKYIPVPSEGSPEARLTPLEQITSDDTKRGIDITETLRIQMEHQKKLHEQLESLRTMQLRIEEQGKALLMMIEKQ

NMGFGGPEQGEKTSAKTPENGSEESESPRPKRPRNEE

>AT2G20570.1 AtGARP28

MLALSPATRDGCDGASEFLDTSCGFTIINPEEEEEFPDFADHGDLLDIIDFDDIFGVAGD

VLPDLEIDPEILSGDFSNHMNASSTITTTSDKTDSQGETTKGSSGKGEEVVSKRDDVAAE

TVTYDGDSDRKRKYSSSASSKNNRISNNEGKRKVKVDWTPELHRRFVEAVEQLGVDKAVPSRILELMGVHCLTRHNVASHLQKYRSHRKHLLAREAEAANWTRKRHIYGVDTGANLNGRTKNGWLAPAPTLGFPPPPPVAVAPPPVHHHHFRPLHVWGHPTVDQSIMPHVWPKHLPPPSTAMPNPPFWVSDSPYWHPMHNGTTPYLPTVATRFRAPPVAGIPHALPPHHTMYKPNLGFGGARPPVDLHPSKESVDAAIGDVLTRPWLPLPLGLNPPAVDGVMTELHRHGVSEVPPTASCA

>AT2G38300.1 AtGARP29

MIPSMEGGGKTNREEEEEEEEEEEEGEESKVSSNSTVEESDKKTKVRPYVRSKVPRLRWT

PDLHLRFVRAVERLGGQERATPKLVRQMMNIKGLSIAHVKSHLQMYRSKKIDDQGQAIAGHKHLFETSTDRNIYKLSQLPMFRGYNHNHDSPFRYGSKISNASLWNSSSQGTERSLIDQI

RPGLIRNASVSNNIRGSDYWTNNKSFQNIYSSSISNHFPKLRHDHHERTNSVTFNSIQGH

SRTFQKFHNGVEENTNHSYCSKTNGKRDASRSIDLDLSLKLRQPEKTILEETETAATTTD

QTLSLSLCPGSSSWKKSRLMKDEEDRTVKIGQESTLDLTL

>AT2G40260.1 AtGARP30

MRSSSQNSENSKTCLSNNIKATTKNEEDKDEEDDEEGEEDEEERSGDQSPSSNSYEEESG

SHHHDQNKKNGGSVRPYNRSKTPRLRWTPELHICFLQAVERLGGPDRATPKLVLQLMNVKGLSIAHVKSHLQMYRSKKTDEPNEGDQGFSFEHGAGYTYNLSQLPMLQSFDQRPSSSLGYGGGSWTDHRRQIYRSPWRGLTTRENTRTRQTMFSSQPGERYHGVSNSILNDKNKTISFRI

NSHEGVHDNNGVAGAVPRIHRSFLEGMKTFNKSWGQSLSSNLKSSTATIPQDHIATTLNS

YQWENAGVAEGSENVLKRKRLLFSDDCNKSDQDLDLSLSLKVPRTHDNLGECLLEDEVKEHDDHQDIKSLSLSLSSSGSSKLDRTIRKEDQTDHKKRKISVLASPLDLTL

>AT2G40970.1 AtGARP31

MREDNPNWFLRWEEELPSPEELIPISQTLITPHLALAFQIGSPNHHLGSKRTTAIYHQKL

QSSTTPTTPTPTPPPMMMNSDFGGGDSTDLGSGSIGGEPARTLKRPRLVWTPQLHKRFVD

AVGHLGIKNAVPKTIMQLMSVEGLTRENVASHLQKYRLYLRRMQGGNGNGITGGHVIVSDSATDRLFASSPVPAHFLSPDYLMPPLEHSYMGKHVITQQNQVVRNLRYEDSEYGHGSMKMLKLFPAGN

>AT2G42660.1 AtGARP32

MAGRSSPGKCSTWLDLNREETVEKNNEEESSVEDEDQITNNVRQYIRSNMPRLRWTPDLHLSFVRAVQRLGGPDRATPKLVLEMMNLKGLSIAHVKSHLQMYRSKKLEPSSRPGFGAFMSGQRSYLMDMIDSRCIPHSDLRHAYNSKTVPSRVLNQDAVVTNLGGNFLMRPSSWFSGLCRNDRDSTENKTLPLLEIRKKNNEKRVRDEEVSSVKRLKSMSGGGIQLPEFGNCRQKPTDDINTMLSLSLLSTSSEP

>AT3G04030.1 AtGARP33

MYYQNQHQGKNILSSSRMHITSERHPFLRGNSPGDSGLILSTDAKPRLKWTPDLHERFIE

AVNQLGGADKATPKTIMKVMGIPGLTLYHLKSHLQKYRLSKNLNGQANNSFNKIGIMTMMEEKTPDADEIQSENLSIGPQPNKNSPIGEALQMQIEVQRRLHEQLELRIEAQGKYLQSVL

EKAQETLGRQNLGAAGIEAAKVQLSELVSKVSAEYPNSSFLEPKELQNLCSQQMQTNYPP

DCSLESCLTSSEGTQKNSKMLENNRLGLRTYIGDSTSEQKEIMEEPLFQRMELTWTEGLR

GNPYLSTMVSEAEQRISYSERSPGRLSIGVGLHGHKSQHQQGNNEDHKLETRNRKGMDSTTELDLNTHVENYCTTRTKQFDLNGFSWN

>AT3G04450.1 AtGARP34

MTLASDFGFPSAISSSFTILEERYHNNFPNTLCVSSGQESMNNNPVPCQVFPLVSGGSSG

GNLFSSSSGFCNGVYVSSSSQARPSVSTVPRDRITVAHVSGEGQRQECPVETHSLQLINQ

PQEQKIMTWSSDQIRGFFDFPVPDPQAASSRTMVSSKEVLSKCEWPDWADQLISDDSLEP

NWSELLGDPNVLNLYSKIETQSSDIARQEIVFRNQHQVDPSMEPFNAKSPPASSMTSKQR

MRWTPELHEAFVEAINQLGGSERATPKAVLKLINSPGLTVYHVKSHLQKYRTARYKPELS

KDTEEPLVKNLKTIEDIKSLDLKTSIEITEALRLQMKVQKQLHEQLEIQRSLQLQIEEQG

RYLQMMIEKQQKMQENKKDSTSSSSMPEADPSAPSPNLSQPFLHKATNSEPSITQKLQNG

SSTMDQSESTSGTSNRKRVRED

>AT3G10760.1 AtGARP35

MREDDSDWFARWEEELPSPEELMPISQSLISPDLALAFDIRTPNHGNGNSNQPHHHHHHQ

TTPPTPSQLQLPSSQANSSAEFAADSADLGSGGAAGDEPARTLKRPRLVWTPQLHKRFVD

AVAHLGIKNAVPKTIMQLMSVDGLTRENVASHLQKYRLYLKRMQGLSSGGGAGSDPATDR

LFASSPVPAHFLHPNRVPSSDHFMPSFVPIATLQQQQQMAAAAAAAAAANPHLQPPQFHR

QIAAAHFGSPTNGGFSSPTSNGQFGSPTSNGFGSPTTNGKFDPSFLAVRQTQQQPIQRMS

TPSLHSPVSNYVEDLESANANGGRTVLTLFPTRDD

>AT3G12730.1 AtGARP36

MMQSREEIRDDSSSGLVLTTDPKPRLRWTTELHERFVDAVTHLGGPEKATPKTIMRVMGV

KGLTLYHLKSHLQKFRLGKQPHKEHSQNHSICIRDTNRASMLDLRRNAVFTTSPLIIGRN

MNEMQMEVQRRIEEEVVIERQVNQRIAAQGKYMESMLEKACETQEASLTKDYSTLFFDRTNICNNTSSIPIPWFEDHFPSSSSMDSTLILPDINSNFSLQDSRSSITKGRTVCLG

>AT3G13040.1 AtGARP37

MYIKAIMNRHRLLSAATDECNKKLGQACSSSLSPVHNFLNVQPEHRKTPFIRSQSPDSPG

QLWPKNSSQSTFSRSSTFCTNLYLSSSSTSETQKHLGNSLPFLPDPSSYTHSASGVESAR

SPSIFTEDLGNQCDGGNSGSLLKDFLNLSGDACSDGDFHDFGCSNDSYCLSDQMELQFLS

DELELAITDRAETPRLDEIYETPLASNPVTRLSPSQSCVPGAMSVDVVSSHPSPGSAANQ

KSRMRWTPELHESFVKAVIKLEGPEKATPKAVKKLMNVEGLTIYHVKSHLQKYRLAKYMPEKKEEKRTDNSEEKKLALSKSEADEKKKGAIQLTEALRMQMEVQKQLHEQLEVQRVLQLRIEEHAKYLEKMLEEQRKTGRWISSSSQTVLSPSDDSIPDSQNMSKTKASSPQPPLPAENK

ASETEDDKCESPQKRRRLENIAESEDPKR

>AT3G19070.1 AtGARP38

MTNLDEFLEELSSIESHSQKIVKIDEHLKKLDEETKEDEDEDAAKIIQDRSTAREAEIAL

LMDLRQNFLPPPSMSSSSLQPPSSSTLAPSSSSSLQPPAPLIDFFRSSVSYSHQPPSSST

LATSSSPSLQPPSMSSSSLQPPASLREFFTSSVSYSHQPSSSSTLATSSFFPSSMPYSVR

PPDSSDRPSLREFFPSSPSSSIQPPESSSSKRARLSNIFPSPLSSSPSPFVNPFLRPQAQ

EPTIPNFINPIPQISPGLPALFPNPNPNPNPNPIPNPIRNPDLVWTNRLQLVFDDAVVRL

GGLFSATPKAINELISEEGLTGDQIRSHLQVLRDRQKAIADKLALE

>AT3G24120.1 AtGARP39

MYSAIRSLPLDGGHVGGDYHGPLDGTNLPGDACLVLTTDPKPRLRWTTELHERFVDAVTQLGGPDKATPKTIMRTMGVKGLTLYHLKSHLQKFRLGRQAGKESTENSKDASCVGESQDTGSSSTSSMRMAQQEQNEGYQVTEALRAQMEVQRRLHDQLEVQRRLQLRIEAQGKYLQSILEKACKAFDEQAATFAGLEAAREELSELAIKVSNSSQGTSVPYFDATKMMMMPSLSELAVAIDNKNNITTNCSVESSLTSITHGSSISAASMKKRQRGDNLGVGYESGWIMPSSTIG

>AT3G25790.1 AtGARP40

MIKNLSNMKNDNQKREKCCEYIEALEEERRKINVFQRELPLCVELVTQAIEAYKREISGT

STDNLYGQSECSEQTTGECGRILDLFIPIKHSSTSIEEEVDDKDDDDEEHQSHETDIDFD

DKNMKSEWLKSVQLWNQSDAVVSNNRQDRSQEKTETLVELIKINDEAAKKNNNIKSPVTTSDGGSGGGGGRRGQRKNRRCWSQELHRRFLNALKQLGGPHVATPKQIRDIMKVDGLTNDEVKSHLQKYRLHARRPSQTTPNNRNSQTQHFVVVGGIWVPQTNHSTANAVNAVASGETTGIYGPMVSSLPSEWPRHSNFGRKISEDRSRCSNNGFFRCSSPAMSCSTRTKTKDAKIIS

>AT3G46640.1 AtGARP41

MGEEVQMSDYDVSGDGDRVSEWEMGLPSDEDLASLSYSLIPPNLAMAFSITPERSRTIQD

VNRASETTLSSLRGGSSGPNTSSSNNNVEEEDRVGSSSPGSDSKKQKTSNGDGDDGGGVD

PDSAMAAEEGDSGTEDLSGKTLKRPRLVWTPQLHKRFVDVVAHLGIKNAVPKTIMQLMNVEGLTRENVASHLQKYRLYLKRMQGLTNEGPSASDKLFSSTPVPPQSFQDIGGGGGSSGNVGVPIPGAYGTQQMMQMPVYAHHMGMQGYHHQNHNHDPYHQNHRHHHGAGGNGAFESNPYMMQQNKFGSMASYPSVGGGSANEN

>AT4G04580.1 AtGARP42

MQDPMVRSYIRSDNPRMHWTDDLDIRFIQVIEKLGGEESATPKRILSLMGVRDLTISHVK

SHLQMYRNKKKEESSKEIKMMREMTQRQSQQYLQIYERATQFIQNQQRLQLDNTEKITPV

LGSSNKSLDQSSKVGLNENRGNDVVVVGGATGEEELSLELTLGRKY

>AT4G13640.1 AtGARP43

MYSAIRSSLPLDGSLGDYSDGTNLPIDACLVLTTDPKPRLRWTSELHERFVDAVTQLGGP

DKATPKTIMRTMGVKGLTLYHLKSHLQKFRLGRQSCKESIDNSKDVSCVAESQDTGSSST

SSLRLAAQEQNESYQVTEALRAQMEVQRRLHEQLEVQRRLQLRIEAQGKYLQSILEKACKAIEEQAVAFAGLEAAREELSELAIKASITNGCQGTTSTFDTTKMMIPSLSELAVAIEHKN

NCSAESSLTSSTVGSPVSAALMKKRQRGVFGNGDSVVVGHDAGWVMPSSSIG

>AT4G17695.1 AtGARP44

MELFPSQPDLYLKISRRREEEQEKESQELQEQEVERRLGFQSKASDLDNKSSNNLIHTLQ

FTSNNEATKINSNQEHKESLDQDLRSIFMMRPIRGIPLYQNQVLDHYYYSSTSPNPFFFS

EVNGQHASRRLITNPNCSFNLHNRHRRQAQPQPPRFTAKRGVRAPRMRWTTTLHAHFVHAVQLLGGHERATPKSVLELMDVQDLTLAHVKSHLQMYRTIKSTEKPTTSSGQSDCENGSQVNSEREARNLTGLWNNSSSEARFQLKAKASSGVDISSNENEWKNRRCPSNERLSSDSSSLT

GTRPETETPNLDFTLATPNLSP

>AT4G28610.1 AtGARP45

MEARPVHRSGSRDLTRTSSIPSTQKPSPVEDSFMRSDNNSQLMSRPLGQTYHLLSSSNGG

AVGHICSSSSSGFATNLHYSTMVSHEKQQHYTGSSSNNAVQTPSNNDSAWCHDSLPGGFL

DFHETNPAIQNNCQIEDGGIAAAFDDIQKRSDWHEWADHLITDDDPLMSTNWNDLLLETN

SNSDSKDQKTLQIPQPQIVQQQPSPSVELRPVSTTSSNSNNGTGKARMRWTPELHEAFVE

AVNSLGGSERATPKGVLKIMKVEGLTIYHVKSHLQKYRTARYRPEPSETGSPERKLTPLE

HITSLDLKGGIGITEALRLQMEVQKQLHEQLEIQRNLQLRIEEQGKYLQMMFEKQNSGLT

KGTASTSDSAAKSEQEDKKTADSKEVPEEETRKCEELESPQPKRPKIDN

>AT4G37180.1 AtGARP46

MVQTETDQRMGLNLNLSIYSLPKPLSQFLDEVSRIKDNHSKLSEIDGYVGKLEEERNKID

VFKRELPLCMLLLNEAIGALKDEARKGLSLMASNGKFDDVERAKPETDKKSWMSSAQLWISNPNSQFRSTNEEEEDRCVSQNPFQTCNYPNQGGVFMPFNRPPPPPPPAPLSLMTPTSEMMMDYSRIEQSHHHHQFNKPSSQSHHIQKKEQRRRWSQELHRKFVDALHRLGGPQVATPKQIRDLMKVDGLTNDEVKSHLQKYRMHIRKHPLHPTKTLSSSDQPGVLERESQSLISLSRSDSPQSPLVARGLFSSNVGHSSEEDEEEEDEEEEKSDGRSSCRNDETKKKRQVLDLEL

>AT5G05090.1 AtGARP47

MREEDSNWFAKWEEELPSPEELIPLSQSLITPDLAIAFDLHRNNNSNSGQPLPQTTPPQP

NSSAEIAGDSTGDEPARTLKRPRLVWTPQLHKRFVDAVAHLGIKNAVPKTIMQLMSVDGL

TRENVASHLQKYRLYLKRMKSGGGGGGSGDSDHLFASSPVPPHFLHPTSRQSSDLFIPSF

VPISTQQQHIAAPPSQFLHRQISAVNFTSPTKATDQAMFLARQQSELQQPVFKPSSLHLH

SQVANYTQDLKSGAKTVLTLFPTRDD

>AT5G06800.1 AtGARP48

MMDNINFEFSNASQGSRLQLQQQPPQPFNLQDLNMIQYNQPSSPWTTETFSGLTPYDCTA

NQSFPVQCSSSKPYPSSFHPYHHQSSDSPSLDQSVSMIPMQPLPDQYMKPLYQRSCSNDF

AATNASSASYSLSFEASHDPQELCRRTYSNSNVTHLNFTSSQHQPKQSHPRFSSPPSFSI

HGGSMAPNCVNKTRIRWTQDLHEKFVECVNRLGGADKATPKAILKRMDSDGLTIFHVKSHLQKYRIAKYMPESQEGKFEKRACAKELSQLDTRTGVQIKEALQLQLDVQRHLHEQLEIQRNLQLRIEEQGKQLKMMMEQQQKNKESLLKKLPDAEASLSLLDPHIHSPPSPFLVHDAEALMLTSYEDTQLQSTKS

>AT5G16560.1 AtGARP49

MSMEGVFLEKTKTNTTTTLPDLSLHISLPDIHQYHHNESSKESSRRSSQLENNNRSSNFE

LSLSHHNHPTARIFHCPDRRTLNLPHQQHYNNPIINGVHQRVDESEISNLHRPIRGIPVY

HNRSFPFHQQNSSLPSLGGGDMDQISILNSSSGYNNAYRSLQSSPRLKGVPLHHHHHHNQ

YGVVGSSDSSSPHHHNHHHHGMIRSRFLPKMPTKRSMRAPRMRWTSSLHARFVHAVELLGGHERATPKSVLELMDVKDLTLAHVKSHLQMYRTVKTTNKPAASSDGSGEEEMGINGNEVHHQSSTDQRAQSDDTSLHQETDISSTQPRWSNSSRETWPLSNNCSSDIDTMIRTSSTSMIS

HYQRSSIQNQEQRSNDQAKRCGNLSCENPSLEFTLGRPDWHEK

>AT5G18240.1 AtGARP50

MYYHNQHQGKSILSSSRMPISSERHPFLRGNGTGDSGLILSTDAKPRLKWTPDLHERFVE

AVNQLGGGDKATPKTIMKVMGIPGLTLYHLKSHLQKYRLSKNLNGQANSSLNKTSVMTMVEENPPEVDESHSESLSIGPQPSMNLPISDALQMQIEVQRRLHEQLEVQRHLQLRIEAQGK

YLQSILEKAQETLGRQNLGAAGIEATKAQLSELVSKVSADYPDSSFLEPKELQNLHHQQM

QKTYPPNSSLDSCLTSSEGTQKAPKMLDNRLGLRTYIGDSTSEQKEIMEEPFFHRMELTW

AEEESLRENHNRPYLSTMVNNAEPRISSSRRSPGRLSIGVGLHEHRGRSSNNSEYTEERF

NENNEDCKLETHTRTALDLNTHDENYGTTRPKQFDLNGFSWN

>AT5G29000.1 AtGARP51

MNNPVPCQSVSGGNSGGYLFPSSSGYCNVSAVLPHGRNLQNQPPVSTVPRDRLAMQDCPLIAQSSLINHHPQEFIDPLHEFFDFSDHVPVQNLQAESSGVRVDSSVELHKKSEWQDWADQ

LISVDDGSEPNWSELLGDSSSHNPNSEIPTPFLDVPRLDITANQQQQMVSSEDQLSGRNS

SSSVATSKQRMRWTPELHEAFVEAVNQLGGSERATPKAVLKLLNNPGLTIYHVKSHLQKY

RTARYKPETSEVTGEPQEKKMTSIEDIKSLDMKTSVEITQALRLQMEVQKRLHEQLEIQR

SLQLQIEKQGRYLQMMFEKQQKIQDNKSSSSEASPKQCNGSFAEVEVGLETLTGDQNESA

SASRKRVRED

>AT5G42630.1 AtGARP52

MMMLESRNSMRASNSVPDLSLQISLPNYHAGKPLHGGDRSSTSSDSGSSLSDLSHENNFF

NKPLLSLGFDHHHQRRSNMFQPQIYGRDFKRSSSSMVGLKRSIRAPRMRWTSTLHAHFVH

AVQLLGGHERATPKSVLELMNVKDLTLAHVKSHLQMYRTVKCTDKGSPGEGKVEKEAEQRIEDNNNNEEADEGTDTNSPNSSSVQKTQRASWSSTKEVSRSISTQAYSHLGTTHHTKDNEEKEDTNIHLNLDFTLGRPSWGMEYAEPSSDLTLLKC

>AT5G44190.1 AtGARP53

MLTVSPAPVLIGNNSKDTYMAADFADFTTEDLPDFTTVGDFSDDLLDGIDYYDDLFIGFD

GDDVLPDLEIDSEILGEYSGSGRDEEQEMEGNTSTASETSERDVGVCKQEGGGGGDGGFR

DKTVRRGKRKGKKSKDCLSDENDIKKKPKVDWTPELHRKFVQAVEQLGVDKAVPSRILEI

MNVKSLTRHNVASHLQKYRSHRKHLLAREAEAASWNLRRHATVAVPGVGGGGKKPWTAPALGYPPHVAPMHHGHFRPLHVWGHPTWPKHKPNTPASAHRTYPMPAIAAAPASWPGHPPYWHQQPLYPQGYGMASSNHSSIGVPTRQLGPTNPPIDIHPSNESIDAAIGDVISKPWLPLPLGLKPPSVDGVMTELQRQGVSNVPPLP

>AT5G45580.1 AtGARP54

MMTRDPKPRLRWTADLHDRFVDAVAKLGGADKATPKSVLKLMGLKGLTLYHLKSHLQKYRLGQQQGKKQNRTEQNKENAGSSYVHFDNCSQGGISNDSRFDNHQRQSGNVPFAEAMRHQVDAQQRFQEQLEVQKKLQMRMEAQGKYLLTLLEKAQKSLPCGNAGETDKGQFSDFNLALSGLVGSDRKNEKAGLVTDISHLNGGDSSQEFRLCGEQEKIETGDACVKPESGFVHFDLNSKSGYDLLNCGKYGIEVKPNVIGDRLQ

>AT5G59570.1 AtGARP55

MGKEVMVSDYGDDDGEDAGGGDEYRIPEWEIGLPNGDDLTPLSQYLVPSILALAFSMIPE

RSRTIHDVNRASQITLSSLRSSTNASSVMEEVVDRVESSVPGSDPKKQKKSDGGEAAAVE

DSTAEEGDSGPEDASGKTSKRPRLVWTPQLHKRFVDVVAHLGIKNAVPKTIMQLMNVEGLTRENVASHLQKYRLYLKRIQGLTTEEDPYSSSDQLFSSTPVPPQSFQDGGGSNGKLGVPV

PVPSMVPIPGYGNQMGMQGYYQQYSNHGNESNQYMMQQNKFGTMVTYPSVGGGDVNDK

>AT5G62110.1 AtGARP56

MNVEGITRSQIASHLQKYRKHMRRFKVNVTKADISKKAMKSNFESSSNINPQCSYNYLGL

TKENLFKSQIGDTLGQSSLLLNNNDSIHSMYDGSIYGLNEPESNLSPMRESVGFSRGVLP

LNEESECAFETSQMSQVPRRGQYGTPSDISVDSNGNGHLGESLGAMNWNLNNNDVRNYESSTSRFSSPFSSFLHNKVQPHHNLISQTDGLLSTSILDTSQVPSFGQYGIPNDVVGMEPEF

GTSYMHSNYNGINTDQVGNLVGLGRERVNVNGNVSLGQNLGSMNWNFDENNMSNHGSSTSRFSSPFSSFEDQSLSHLNEETNELVPGLENLSLYNDHQSINPFLGNTINSHFVENQDQGL

VNNENLEFANDDQLERNEILTTYSVEDINNLSINNVKLNSNGETLNSTAANLEMLFPTLD

MNIVNQHQGQVLSGGNLEFANDNQLERNAILAGYSIEDINNLSINNHQGQVLSGVNFEFA

NDNQFERNAILAGYSIEDINNLSINNVNDNSNEETLNSIAANNEMNFTILDVNTINQVNG

NSNEETLNSIVANSEINFTTLDVNTINQVDNNFNEETLNSIAANSEMNFTTLDVNIINEE

EQGAVDMMDIVITFDEEEEDVAALTDIPEQDVEILTDVPEQGVTDLTDVPLTFDQDWKDD

GLMNFMLDVDDMF

>MDP0000124301 MdGARP1

MAALQRVAQSSVSTTASSYGSCKVGGGVLSPSAGIEMAVPNQFPAGLRVLVVDDDTTCLR

ILELMLLRCLYQVTTCSEATVALNLLRERKDCFDVVLSDVHMPDMDGFKLLEHVGLEMDLPVIMMSADGRTSVVMRGIRHGACDFLIKPISEAELKNIWQHVVRKKWNGSKELEHSGSLEDNDPHKRGNNDFEYTSSVNEGTEVSLKGHKKRINAKEDDDGDTENDDLSTSKKPRVVWSVELHQQFVTAVNQLGLDKAVPKRILELMNVPGLTRENVASHLQKFRLYLKRLSGVAQQQSGIANPLCGPVDSNGKLGSLSRFDFQALAASGQIPPQTLAALQAELLGQPAGNLVPAMDQPALLHASLQAPKRPPVEHGVPFMQPFVKSQSNVSKHFPQSVISAEDASLGFGQWRSNSRSTVAPSNDHGGLSTQNSNLLMGIVPQEQRQHKRTQQQSVLTEPSRSFNVQPSCLVVPSQSSTG

FQAGNSPASVNQSSSFNRSTVVDYSLPSDQSNNSLNVGHIPTEILKL

>MDP0000140568 MdGARP2

MTVERVLDEPRDQFPIGMRVLAVDDDPICLKLLEALLRRCQYHVTTTSHAFTALKLLREN

KDKFDLVISDVHMPDMDGFKLLELVGLEMDLPVIMLSVNGDHKLVMKGITHGACDYLLKPVRIEQLKNIWQHVIRRKKVDSKDQNSGNQDKSAGCGEGGVGSVGTGNSDQNAKLNKKRKDQNDGEDEDHDEDEYENDDPSTQKKPRVVWSVXLHRKFVAAVNQLGIDKAVPKKILDLMNVEKLTRENVASHLQKYRLYLKRISCVANQQANMVAALGTSDSSYLRTASMNGVGNYHXLTGPAQFHNNGYRSFPPXGMIGRLNTPAGVGLHCLPSSGMFQLGHAQNPSNAINDQPVMFPGNRNGNMLPPHLELDQQHNRGSTYGAPIYPDSSGFTDTKVTNRSNDPFGLTDNTMLRHTRDSEVGRNHVMQSSVSMPSLKPVLAXPFLDHGRLNDNWPTAAQPSAFRSNSFSAVDSFKQPTLLDSRIGSQIPASTMSNNSDPTINNDLFLGWDEPKQDSPFQSDVMCSSMNNLMPVNDNVAPSHQNLDARSSAFQRNSEFNLIGQPNFLDPLLMNYDGVEISTMDAPLKLKQQGYLMDQRKPQGVYVPHNAGSLDELVSSMIKQVRSFSTGLFYCQIHKHPVRPACWPDXKNSWAGWWTRQNEIISHCLPAAYMYRLQLLGRLLAHLHAINFLSKGDRRNHREEGSIPV

>MDP0000155328 MdGARP3

MAALQRVAQSSVSTTASSYGSCKVGGGVLSPSAGIEMAVPNQFPAGLRVLVVDDDTTCLR

ILELMLLRCLYQVTTCSEATVALNLLRERKDCFDVVLSDVHMPDMDGFKLLEHVGLEMDLPVIMMSADGRTSVVMRGIRHGACDFLIKPISEAELKNIWQHVVRKKWNGSKELEHSGSLEDNDPHKRGNNDFEYXSSVNEGTEVSLKGHKKRINXKEDDDGDTENDDLSTSKKPRVVWSVELHQQFVTAVNQLGLDKAVPKRILELMNVPGLTRENVASHLQKFRLYLKRLSGVAQQQSGIANPLCGPVDSNGKLGSLSRFDFQALAASGQIPPQTLAALQAELLGQPAGNLVPAMDQPALLHASLQAPKRPPVEHGVPFMQPFVKSQSNVSKHFPQSVISAEDASLGFGQWRSNSRSTVAPSNDHGGLSTQNSNLLMGIVPQEQRQHKRTQQQSVLTEPSRSFNVQPSCLVVPSQSSTGFQAGNSPASVNQSSSFNRSTVVDYSLPSDQSNNSLNVGHIPTGNPKTSGXLGGYSGPGSXCATSCLVNADNSTSYQNSTATFSDSRELPGFLHNTANSXGFYVDKSGEMLDQGPLRNLGFVGKETCIPSRFAVDDFESQMSNLNPGRIHVESSGTLVKQEPSEDYVDNAKLGIPILHQYS

SSDFMSPFAD

>MDP0000202704 MdGARP4

MEDMSPKESNGLPSFAHGLQILVVDHDTLSLMCIASTLEKYSFKVTTTALASVALSMIKE

QKYHYDLVMANISMPDKDKFSLLQVLHKNEIPVIFMSSEVNIDVAKKALAEGACFFLQKP

VSSEDLKNVWQHAYRKVRNPRKDTHKTKCGKKIHEAGGVLIPPTGGIRIHEVGGVSRLPT

GRELCLDTQDTRDERQIAAENYTQGALGINRPVDDKEDQEKAKKVKLNTEQDCDEEGMENQDCDGSSKRKKWHPVWTTELHLKFTAAISALGDQKARPRRILKWMNVPDITVRQVASHLQKYRKNVHRIQEVGTTSLPSLGKSSIWNNRNEFPPARQTSLVCHLYEQRTSSSGAQGNPTQLMTPNSFTGFNDYRRQNLYTEHRVMSHNTNHQSESLYDFYLRTQGKFENLDQILETNSFTLGADMNKIERNQPLGLEEIMFKASERTLPSTNYMTPEFGSPYISANTFQVPNASVSINQA

QNYCPAPTAPFNAQNQDHFSAEATRTTEVGLNQAQFYAPAPTTPINSQNQNHFSAEATRT

TEVGMNQAQFYAPAPTTPINSQNQNHFSAEATRTTEVGMNQAQFYAPALNTPINSQNENY

FSADVMGTTEAGMNQPQYYTPAPTAPIYFQNQNHFSAEVTGTTDVLEVVPEQIPSVNETN

AETFPANFTGGSQELDAAAREAHPASSNNNQPMSEYDDLLKLLEEDPEKFNWFEPFDSAP

NAGDANRYRAWLTETLLEKSPDSP

>MDP0000224740 MdGARP5

MHLSNGMGXMSTASSSGTWKSGDVVSDQFPAGLRVLVVDDDPTCLMILERMLRTCLYEVTKCNRAESALSLLRENKNGYDXVISDVHMPDMDGFKLLEQVGLEMDLPVIMMSADDGKSVVMKGVTHGACDYLIKPVRIEALKNIWQHVVRKKKNERKDXEQSGSVEEGDRQQKPSEDADYSSSANEGSWKNSKRRKDEEEETDERDDSSTLKKPRVVWSVELHQQFVGAVNQLGIDKAVPKKILELMNVPGLTRENVASHLQKYRLYLRRLSGVSQHQNNLSNPFMSPQEASFGAMSSLNGLDLQTLAVTGQLPAQSLATLQAAGLGRSTGKSGIPMPLVDQRNLFSFENPKQQHMSSSKPMNLLXGIPTTMEAKQLANLHQSAQTLGAANMQVNAHGVQNSSLLMPXPQXQPRGQILNESSGNHVPRHSSSVGQPIISNGMGNGVLGRNGIVDSGRGVGYNPVHQNSSMLNYPLNNSSQLPGNNFTLGSTPVMSSLASKGAFPEDVTSDIKRSSGFMPSYDMFNELQQPKSNEWDLQNVGLTFDVSQHTHSIHGSLDPSVLVHQGFSSSQRGGQSRNPSIVGKTMFSVGEGSHFGNMQNTGHHLNGRIVDNTVRIKAETVPDATSQTSFFHEQYGQDDLMSALLKQQDGLGPAENEFDFDGYSLDNIPV

>MDP0000228719 MdGARP6

MLSPPLSHLTLAHSLSLSLSLLFYQLVTLFELSSPVHMRRPKRGSSSMCRDKNLKGPQSD

IMPTSNIPIRLRILVLDVDADFLTFISGLLKLLKHEVVTFMNPIQALSALLLAEHKFHLV

ITDLYLPDIFSGLQVIRRVRDEFKLPLIVASADNSESTILAALENGAVHYTVKPVCFSDV

KNLWGLVLGANETTRLDSNNNITNRRASVDGKSSSSTDGSNNSKRKRSSEEGKSTYISTK

KHKVEWTSELQDRFLEAINYIGLDKAVPKRILEVMNVEGLTRENVASHLQKYRMFLRKVA

AKISVSKLSVERAQQTRRALLYQNILVERQASTLQQRRSFHENFELLVNQNTAWLLNRLP

RGANDHEASSSRCLPRSGSYNTRQNPYVLNAQTHSGKQPLMMNTPNRFQPANYCGIFQSNSAQRNWSITNTSTDVAYNFGSNNANGVKIGTNIPSQILFSSSWDTSNGVKNYASGASSSQ

LSPMFAEGNNIGQENVNASAXEDPLQQQQQQYGGTDLVNASNNSSSHVTGVVDCSSSTEI

HLKDSTTGHDDFDISDIFWNELQLESVTDDKGKGKEVMNSEQDNGDDFDSITLDELNSEP

ATCDKENQGDMMDLEFDGDGLNFVDLSPDEVCSLCKLLSNV

>MDP0000237396 MdGARP7

MVCTANDLQQWNDFPKGLRVLLLDGDTTSASQIRSNLEAIDYIVTAFCNETEALSAFSSN

PESFHVAIIEVNANNGHWGFQFLETVKELPIIMTSNIHCLSTMMECIALGAVEFLLKPVS

EDKLRNIWQHVVHKAFNAGGSVISETLKPVKVSVVSMLQPKQENEEQNCKISMETENVLRVHENDQEQSAGSDKYPAPSMPQIKQGSRLLDHGDCLYQINYMSEKVSGEHDSESKSIETMFCNPIDERNLRMREPRHMPRKTVIKEENDSGDGSKSEVNMSHHPDNIDSGNDNGAAAENLSKASVPNXSCKSNXKKMKVDWTPELHKKFMKAVEQLGVDQAIPSRILDLMKTEGLTRHNVASHLQKYRMHKRHILPKDEDRRLPPPHHPPGARDQTHQTSYYPHSHRPIMAFPPYHYNNTTLSMPPAYPMWGSQPPAGSHPATVQMWGQPGYPHSHWQLSDQSWQYWNPYPGVMYCSSSSQMQAEAWGCPVMPPEQGGYSSYPQNASGFQSAGGHVVENGHRYSMPRNSFNIHLSEEVVDKVMKEAISKPWLPFPLGLKPPSTEGVLAELTRQGICNIPQINGSHTQG

>MDP0000248863 MdGARP8

MVCTANDLQEWKDFPKGLRVLLLDGDTTSASQIRSKLEAMDYIVTAFCNENEALSAFSSN

PESFHVAIIEVNANNGHWSFQFLETAKELPIIMTSNIHCLSTMMKCIALGAVEFLRKPLS

EDKLRNIWQHVVHKVXRVWKCCFKIYHKVLCNSCKQKFSDFVXPEQAFNAGGSVISETLKPVKESXVSMVQPKQENEEQNCKISKETENVLRVHENDQEQSAGCDKYPAPSTPQLKQVSRLLDHGDCQDQINCLSEKESGEHDNETKSVETTCNNPIDEGNLLTIEPHHMLRKTAVKKENVSGDGSKSEVNMSRHPDNIDSGNDNGGAAENLXKASVDWTSELHKKFVQAVEQLGVDQAIPSRILDLMKTEGLTRHNVASHLQKYRMHKRHILPKEEDRRLLQPHHTRDPIHQRSYYPYSHRPIMAFPPYHYTNPTLSMPPAYPMWGSQPPASSHPXTVQMWGQPGYPHSQWQPSDQSWQYWNPYPGVMHCSSSSQLQADAWGCPVMPPEQGGYSPYPQNTSGFQNAGGHAVENCHGYSMPQNSFDIHPAEEVVDKIVKEAMSKPWLPLPLGLKPPSTEVSNLXHAVLDXLQKLDLKEXGEDNGXPDEVQKLDSIEKVEEDGDXEWQKVDLKAXEDXASELQKVDLKAEEGGXGLEEEXKSSGEQ

>MDP0000290818 MdGARP9

MHLSNGMGSMSTASSSGAWKSGDVVTDQFPAGLRVLVVDDDPTCLMILERMLRTCLYEVTKCNRAEIALSLLRENKNGFDIVISDVHMPDMDGFKLLEQVGLEMDLPVIMMSADDGQSVVMKGVTHGACDYLIKPVRIEALKNIWQHVVRKKKNEWKDVEQSGSVEEGDRQQKPSEDADYSSSANEGTWKNSKRRKDEEEETDERDDSSTLKKPRVVWSVELHQQFVGAVNQLGIDKAVPKKILELMNVPGLTRENVASHLQKYRLYLRRLSGVSQHQNNLSNSFMSPQEASFGAMSSLSGLDLQTLAVTGQLPAQSLATLQAAGLGRSTTKSGVPMPLVDQRNLFSFENPKLRFAEGQHHLSSSKPMNLLHGIPTTMEAKQLANLHQSAQSLGAVNMQVNAHGVQNSSLLMPVAQSQPRGQILNETSGNHVPRLSSSMGHPIMSNGMSSGVSGRNGIVDSGRGGGYNPMQQNSSMXNYPLNNSSQLPCNSFTLGSTPVMSSLTSKGAFPEDVNSDVKISSGFMPSYDMFNELQQXKSNDWDLQNVGLTFDPSQHTNSMHNSLXPSVIVHQGFSSSQRGGQSGSVGKALFSMXEGSHLGNVQNTSQRLNGLVVDNTVRIKAESAADAXSQTSFFHEQYGQDDLMSALLKQVXFLPSFHFTVFEFLDTCFELSRAXFTHEISFVFQQQDGLGPAENEFDFDGYSLDNIPV

>MDP0000304202 MdGARP10

MYLRNNHGYFNENDGPRVSWESPKTEMLVQFLTYHQRWEPSYIRQRMLPMLSTIFREMAKNPVKSLLYGQYEFNSIDRLKIRYGHQFYLVNYLIPPEDSDKQQDDVMEVDESLSPLDESD

VPTREISGXCCFLLTDENMDLVHAAFPVEVDRFLQEKELKQLKRRKTTGGKPETPGSEVW

SHTAPYRSDRIEHENKTTRTXFXDANVDPENIVKEVFASTNAVVSVDNHMRLLXHTESKQ

SCEAMNDEKAVPVESMKNCSPVPEAVTVLAVDGDSACLTLLSRMLCKLGYKVLTAKRACDALSIAQKKESELHLVLTEVHLPDMDKYELLERMRAVSKLPVVIMSDDDDENAMLGGLFKGAVYYIVKPLTMDSLKNLWQFAFISNRDAVTTXNNRDXVIDLTADDSSGEFQQEYTSNEGLEIESIXKKEKQISXRKSLKRKNPEGMNKDKGGDNSDSTCTQKKPKMVWTNELHKKFVQAVTLLGVDSAHPKKILQHMNVPGLWKENXSSHLQKYRLSLKQEQEEIMMXKARETTESSFPTSPLNFPGGSSNQFPKXSPFTSTAYQPXIRSHCRNPSSNMTRPSPGSTFVCLPSTQSSNESTFASNHFSSNYKNGQPTLSNHFGKVASYMNFDFPILLHNYIQQEEHQQQLIQPRLLFSPP

SQMPSPLPPPEEQQGNNIFGSENQLQPQVLLAPPSLPPPAQEQGNHDDIFGLGKQFQTND

VXLSPQRKXPSLAPTPATQEQKETDMFGIERGDMDDLFDIAKGTTQQFHDVNFDDFW

>MDP0000305167 MdGARP11

MESSFSSPRNDTFPAGLRVLVVDDDPTWLKILEKMLKKCSYEVTTCGLARDALHLLRQKK

DGYDIVISDVNMPDMDGFRLLEHVGLEMDLPVIMMSVDGETSRVMKGVQHGACDYLLKPIRMKELRNIWQHVFRKKIHEIRDIESHESFEGIQFIRSGSDQYDEGYFLSADDLTSSRKRK

DVDNKYDDKDFADLSSTKKARVVWSVDLHQKFVKAVHQIGFDKVGPKKILDLMNVPWLTRENVASHLQ

>MDP0000307383 MdGARP12

MQEDNDNVFPLIMTTCGLARDALHLLRQKKDGYDIVISDVNMPDMDGFRLLEHVGLEMDLPVIMMSVDGETSRVMKGVQHGACDYLLKPIRMKELRNIWQHVFRKKIHEIRDIESHESFEGIQFIRSGSDQYDEGYFLSADDLTSSRKRKDVDNKYDDKDFADLSSTKXARVVWSVDLHQKFVKAVHQIGFDKVGPKKILDLMNVPWLTRENVASHLQ

>MDP0000311914 MdGARP13

MAALQRVAQSSVSTTASSYGSCKGGGGVLSPSAVEMAVPDRFPAGLRVLVVDDDTTCLRI

LELMLLRCLYQVTTCSEATVALNLLRERKDCFDVVLSDVHMPDMDGFKLLEHIGLEMDLPVIMMSADGRTSVVMRGIRHGACDFLIKPVREAELKNIWQHVVRKKWNGSKELEHSGSLEDNDPHKRGNNDIEYTSSVNEGSEVSLKGQKKRINAKEDDDGDTENDDMSTSKKPRVVWSVELHQQFVTAVNQLGLDKAVPKRILEMMNVPGLTRENVASHLQAWLYLKRLNGVAQQQSGIANAFCGPADSNGKLGSLSRFDFQTLATSGQIPPQTLAALQAELLGQPAGNLVPAMDQPALLHASLQGAKHPPVEHGVPFVQPFIKSQSNVSKQFPQPVISAEDVSPGFGQWRSNNCSTVAPSNYHGGSSVQNSNFLMGVVQQEQRQHDRTQQQSALIEPCRSFNVQPSCLVVPSQSSTGFQAGNSPASFNQSSSFNKSTVIDYSLLSDQSNNSLNIGHIPTGNLKITSTLGGYSAPGSISP

TSCILNTDNNSTSYQNSAATFSDSRELPGVLHNTSNSQGFYVDKSGEMLDQGPLRNLGFV

GKETCIPSRFAVDDFESQMSNLNRGRIQVENSGALVKQEPIVDYVDNEKLGIPILQQYSS

SVYFVVSRCLLISMTRTDCFTLSKCSVEIAVEVCVLFLRLDLIDVPAPDSGAERPLASET

LEKLRTNVGEYQCRSGGK

>MDP0000320381 MdGARP14

MESSFSSPRNDSFPAGLRVLVVDDDPTWLXILEKMLKKCSYEVTTCGLATEALCLLREKK

NGYDIVISDVNMPDMDGFKLLEHVGLEMDLPVIMMSVDGETSRVMKGVQHGACDYLLKPIRMKELRNIWQHVFRRKIHEIKDIESHESLEGIQLIRSGSDQYDEGYFLSXDDLTSSRKRK

DVDNKYDDKDFADCSSTKKARVVWSVDLHQKFVKAVHQIGFDKVGPKKILDLMNVPWLTRENVASHLQKYRLYLGRLQKENDLXSSFGGXKRSXSPSKDAQGSFGLQNSISIQQNDLAHGSFXVSGNNLIAQCVDPKSHEGDAKGIVSEPVAEIKKGSNXNIPDSQKTRSLQIVDFNNSCVEPKNGVIGNVPDSQKIRISPLDFNNSYAETKKGLNGNIPDSQKIXTPQVRPSHSFTSVE

SEVNFTEFESTIRTKYSWNEIQLKKEQKPLIQLNSGFNKPPLSGPHNHFQVDRLQSIPSI

SPRPSIVEGDVTGPAKSKPSYSEYINNQGSQVSPTISTADSFPDQIKSCVVNHQVSETIS

SSTSNMKNQGFNLSSXTDLESAQRSLIVGSASPFASLDDDFQICWFQGDCYGMNLGLQNI

EFPEYNDPALISEVPAHLYDALRFDYPCDVTHQNILS

>MDP0000607144 MdGARP15

MTVERVLDEPRDQFPIGMRVLAVDDDPICLKLLEALLRRCQYHVTTTSHAXTALXLLREN

KDKFDLVISDVHMPDMDGFKLLELVGLEMDLPVIMLSVNGDHKLVMKGITHGACDYLLKPVRIEQLKNIWQHVIRRKKVDSKDQNSGNQDKSAGCGEGGVGSVGTGNSDQNAKLNKKRKDQNDGEDEDHDEDEYENDDPSTQKKPRVVWSVXLHRKFVAAVNQLGIDKAVPKKILDLMNVEKLTRENVASHLQKYRLYLKRISCVANQQANMVAALGTSDSSYLRTASMNGVGNYHTLTGPAQFHNNGYRSFPPSGMIGRLNTPAGVGLHCLPSSGMFQLGHAQNPSNAINDQPVMFPGNRNGNMLPPHLELDQQHNRGSTYGAPIYPDSSGFTDTKVTNRSNDPFGLTDNTMLRHTRDSEVGRNHVMQSSVSMPSLKPVLAXPFLDHGRLNDNWPTAAQPSAFRSNSFSAVDSFKQPTLLDSRIGSQIPASTMSNNSDPTINNDLFLGWDEPKQDSPFQSDVMCSSMNNLMPVNDNVAPSHQNLDARSSAFQRNSEFNLIGQPNFLDPLLMNYDGVEISTMDAPLKLKQQGYLMDQRKPQGVYVPHNAGSLDELVSSMIKQVRSFSTGLFYCQIHKHPVRPACWPDXKNSWAGWWTRQNEIISHCLPAAYMYRLQLLGRLLAHLHAINFLSYGQILVH

>MDP0000121180 MdGARP16

MNEQKIDCQERIQQSHDYISKFVSTSYGYSXSTRHXLLSKTRIQWTQDLHEKFVECVNYL

GGAYKAPPKSILKLMNVDGLTIFHVKSHLQKFRNSEYPPGSAKGKXEKRTTSNVEPQLDV

ETXLHIKKAIQLELDVQQHLHEQLEIQRNLQLRIEEQGKQLKNIIELQLKTIIDLQLKNS

DTTFYEGAPLSSLDDEFIDKMHRGYXRNXDVKKRKLEDASGNDDDISTNSEDHLRSLLEP

LAKSQLVDLLAKLGSQYPSIAEEIKGVASADPVHRKLFVRGLAWNTSSETLRAAFSEHGE

IEEGAVIYEKASGKSRGYGFITYKHMESTQCALRAPSKLIDGRLAVCNLAAEGLSGTPAT

LDLTQRKLYIGGLSPNVTSEMLLHFFGRHGDIEEGSVAYDKDTNESRGFGFVTYRAVEAA

KKAIDDPQKTLGGRNIIVKLADSHKGRTVQTQLPPAMVPMALPLVPPGYPQPGKAHAGTP

PVGYSYPQTIAAYPDSSYPGPQPPYPAQSQIPYPYYIGKQ

>MDP0000124053 MdGARP17

MVHQNMQNQNMNLVLSTDAKPRLKWTPELHQRFVEAKYRLGKSQQSENSADIKQEDYKEIQSSDGHFGADISDEDHSQINESLQIAQSLQLQMEVQRKLHEQIEVQRHLQLRIEAQGKYLQSVLKKAQETLSGYSSSSVGVELAKAELTQLVSMVNNGCPSSSFSELTETGTSTLKDVERKQMRGSMDSSLTSSESSGRKEEKLPKNSNATCVELPLMGIHPDNKAWNNSASNHVFGSKRSSSPISDGVSVKQPVAKRTQTQRDKGGNHLRKSGLLATFDLNSKYQSDNIDSGPKVIDLNCKGI

>MDP0000124687 MdGARP18

MGSVPPELSLDFKPTFVPKTISDFLKEVSMIGNVSEKLSKLDDFVKRLEDEMRKIDAFKR

ELPLCMFLLNDAIRALKEDAMQCAAPKVQPVLEEFIPMKKDCDKNKGGSGNNNKKEKDSRDKKNWMSSVQLWNTDNYHQHPSSDSPYDRKRVSEIDSKRNEAENGVANEDPFQTCRNRTGGRAFMPFKGYPAFSVTPLRLEEKEELPVHGLSLLTPGIKNPKEESTSSGSRSTCGRSVSFSTANTQSNTRTPPQQPTSRKQRRCWSPELHRKFVNALQQLGGSQGPNDISMNLLIERPNI

CCITDIHGFGIPKDELIVSLYTAYIVVVDYYTLYSPTIAVSYLRFYDIAVATPKQIRELM

QVDGLTNDEVKSHLQKYRXHTRRVPGAAAAGSEKQSVGLGVLWMSQDQYMDSSKASSSQSGSPQGPLQLTGTGGDDEDNEDAKSESYSWKGHIHKPGKDDV

>MDP0000128135 MdGARP19

MASSPSELSLDCKPHSYSMLLKSFGDLQASHLHDQPQTQXLEEFLSRLEEERLKIDAFKR

ELPLSMQLLTTAVEASRQQLQAYRTNNIQGQTRPVLEEFIPLKHSTSEGSEKPTNPTSDK

ANWMTSAQLWSQATDHNPSATSKXQSTITSGPKEADIGFSVSPKLGLDTKQPRSYGGGAF

LPFSKDRSPTLRPLPELALASPENKAEIILEDHHENGNNNSSSAAGNCNEQAAAAAQTHR

KARRCWSPDLHRRFVNALQMLGGSQVATPKQIRELMKVDGLTNDEVKSHLQKYRLHTRRPSPSPQAGGGPAPQLVVLGGIWVPQEYATATHNGPTALYSPHPGSHAPTHYCATPMPQDFYATQQHQQQLHHHTLQHQLHVYRTSTASHTQSSPESDGRGAGDRSESIEDGKSESSSWKGTESGDHVHMNGGDQRNGGVLAARRSEDGGDSNGSQITLKF

>MDP0000132340 MdGARP20

MSEFGASKATSSSFPLLHTHLGDKYSNLQDSFQVSSQREKTIYSMLPQASSPGNLFSSSS

RPNDVHISSVPPCEQRPQNSSFIFKSRVDENTEKSLSLPHSSHSPLSEVQPTTLINYPEE

HRDISWCPDSLQEFLHFSENVPDQNGLVDSSAGVITSEENAGKTHWSDWDPLISFDDTLD

PNWELPGDVDAIDPKPKQSQIQQHQLQIQQHQPQIQQHQPVQSVEFHPGPEPLSSAPPTK

ARMRWTQELHEAFVEAVDHLGGSERATPKGILNLMKVEGLTIYHVKSHLQKYRTARYKPESSEGPYEKVSTLVDETNPLDMKGSMGITEALRLQVELQKRLHEQLENQRKLQLQIEEQGKCLEKMFEQQRKFEDNRVKSGSSTVDDHSTPLSNIACPSPGDDKPETSKHHPDKTAISAPL

EDGSQDASSKAQELECLEDLDGVESGVLRTKRARNGXKKQNSQELSRLTETNQADRNQSLKVSLCCFVNLDKLVLLRPKVKALVLPASKSEGSSFAASKKNHEFTFYVYNSQIQYC

>MDP0000133017 MdGARP21

MVKEKYDEFKIWPSLAHQYHIIGKQNIKRLGMKSESTSMEEVSKSSPSTSKKEYDHEDDE

GEEDEVDEDGLQLSNNGVSSSNSTIDENHEKKGASGSVRQYVRSKTSRLRWTPDLHLRFV

HAVERLGGQERATPKLVLQLMNVKGLSIAHVKSHLQMHRSKKMEDPNQVLTDXAGFFMEGGDNQLYNLTQLSMLQSXKKWPSSGLRYGATDHSXWQGRHHHHQIYGPYRSSRTTALLDHNTRNIELYGSVAERILGSNNNNIRTTSANQLHTNLPSPNPGQITWRRHQITRDESCQTVIHRPCQDHHSWSAAIRDHQGQNMLKRMAFDXDNSDLDLNLSLKVPPKHGHGFGKGLQCGGDNHQKLHMGCSLSLSLSSSSSSKLGVKKLEGTTGYGDSKHGKNMASTLDLTL

>MDP0000142146 MdGARP22

MAVFACYPFRIVIRCREIMLITCFGGLFCRSSPYNLVXPNYWFRIYRFPPVEVGILHHVF

DRLKKAELSSAIMSHHGVIXVXQSETTKGVKPSYCNSPSPIHDFLGSESQGRSVTASDCS

SPRVSPFIREKSFGSPTNMQGSPVQHSKSAFSRSSVFCTSLHQSSSSSSETSRQLGNLPF

LPHPPTYGQSISAVDSKSPLLFSEDKSNQYDDEQSEDLMKDFLNLHGDSSHGNFHGISYG

SDTLALTEQLELQFLSDQLDIAITDNGENPGLDEIYEIPQPSPKPAIGLTTCSEGCRLTT

PLVDALTSHPSPGPASAHRPRMRWTPELHERFIEAVNRLDGAEKATPKGVLKAMNVEGLT

IYHVKSHLQKYRLARYMPEKKEDKKASSSEEKKATSTINESDGRRKGSIHITEALRMQME

VQKQLHEQLEVQRVLQLRIEEHAKYLRKILENQQKAGSALLSPQALSSQTTNSAHVSAQX

PSSTCVSPPQPAVSDSSSPQSLKHKATDCSASEPPTCTKKPRLEERPDEGVVENPQL

>MDP0000145790 MdGARP23

MELFPAQPDLSLQISPPNSKPSSSSSSTSWRRSSHQRHQEGVDLGFWRRALDSRNSTSDE

LSNTNHRPQHYNNITASTSCNGVNGNLFHPFQFQQSHYFPQVPQTDVQQELQQSDELLGF

LRPIRGIPLYQNPPPPPPNNFFPFSSSQKPVNFDNNSATSTSTITSSTPLFQAAAHHHQG

HHGGLLRSRIFSRFPAKRSIRAPRMRWTTTLHARFVHAVELLGGHERATPKSVLELMDVK

DLTLAHVKSHLQMYRTVKTTDKAAASSGQSDVYENASSGDTTEDIMYELQNPRRSCEQLPSAQHGSRSTTVQDNKEYYGLWSNSSR

>MDP0000151190 MdGARP24

MFPVLMHEGGGMVGEEDMQMQGHSHRGGGDPCLVLTSDPKPRLRWTADLHERFVDAVTQLGGSSKATPKAIMRTMNVKGLTLFHLKSHLQKYRLGKQSGKDMADISKDASYLLESPATGNTSPNLPTSDLNEGYEVKEALRAQMEVQSKLHVQVEAEKHLQIRQDAERRYMAMLERACKMLADQFIGGSVTDTDGHKCHESGNKNLKGTSLDKLGFYSSQSTDAVGVHGIGEELPICIHTQRADCSTESCLTSHESPGGLTLEGSPGGGKKRMLSLDTGNGSLIWGEAKVRTQEISVAPIDPPHGIARYGM

>MDP0000158654 MdGARP25

MVKEKYDEFKIWPSLAHQYHIIGKQNIKRLGMKSESTSMEEVSKSSPSTSKKEYDHEDDE

GEEDEVDEDGLQLSNNGVSSSNSTIDENHEKKGASGSVRQYVRSKTSRLRWTPDLHLRFV

HAVERLGGQERATPKLVLQLMNVKGLSIAHVKSHLQYFSTFSGFDSNHQMHRSKKMEDPNQVLTDXAGFFMEGGDNQLYNLTQLSMLQSXKKWPSSGLRYGATDHSXWQGRHHHHQIYGPYRSSRTTALLDHNTRNIELYGSVAERILGSNNNNIRTTSANQLHTNLPSPNPGQITWRRHQITRDESCQTVIHRPCQDHHSWSAAIRDHQGQNMLKRMAFDXDNSDLDLNLSLKVPPKHGHGFGKGLQCGGDNHQKLHMGCSLSLSLSSSSSSKLGVKKLEGTTGYGDSKHGKNMASTLDLTL

>MDP0000159434 MdGARP26

MSSSFPRLHXPLDDKYTNLPDSFQVSSQREKSIYSMQLQASSPGNLFPSASRPNDIHISS

VPPCEQSSQNSPFIFKSPVDGKSLSLHRSSHSSHSPHLEVQPTALINYPEENKDISWCPD

SLPXFLHFSESVPDQNGLVDSSAGVITSEEHAGKTNWSDWDXLISFDDALDPNWELPVDV

DTMDPKLTRPQVQQHPPQIQQYQLQIQQHHPQIQQHXPQIQQHQXQIQQHQSVQAVEFRP

SPEPLSSVPPTKSRMRWTQELHEAFVEAVNHLGGATPKGIVNLMKVEGLTIYHVKSHLQK

YRTARYKPESSEGPCEKVSTPVEETNPLDVKGSMGITEALRLQVELQKRLHEQLENQRKL

QLKIEEQGKYLEKIFEQQRKIKDNRVTSGSSTXDDHPTPLSNIXCPXPGEDKPETSNHQH

DKTAISIPLEDGSQDASREAHEPECPEDPDGVEXGAQPTKRARNG

>MDP0000164300 MdGARP27

MQLPQTMSVAXLFTPKIXSFYEEFYGFKGVKFVKGIILSSFDIDSDGKRWIPXKISKXFF

WKCCTGRFVLATLCFVLANPYKFRAATPKQIRELMKVDGFTNDEVKSHLQKLEVILHVIS

DEFFYTYM

>MDP0000167868 MdGARP28

MDLVSGGNSLNNSNLASKQRLHPTHELHERFVDAVAQLGGPDRRRNLDLTSPSHKEQPKG

YLFNWTPLSPAYSPSSSPTSASTLSPISPSRLGPTRRPSNGSRSSRVCRNFVDHKFEELD

RDRDGHLALNKLHLAVANINLQLLMHMLLPTAATPHLSSKLSHLADSLKLERQFLRVPFE

HYKKAIHANHHVRKKEISSVINGVFEATDQEKLPKPRIKNLISLVNKIQRACTALSNYGE

GSALPTLWDSFPSIAVVGG

>MDP0000169910 MdGARP29

MPLEGIFREPSSATTTTSSXPIPDLSLHISPPNTSSNSSSIXNTTISETQNPTTSNSQAH

TELSLGRCFTGGAHEQEPPQNPYQQQSFHLHRSNSHLSHLNHGVSLLDVSSSEGLRPIKG

IPVYHQNRSFPFLPMDNMITRDHHKDSRMCFYPKPPPPSSYNHLSFPSAASCSPNTPSAP

YFGGGAGGLDPMSRFTRFSSVDVFKSNNQLHHHHHLHQLHHHNHYGIGGVGPMSEGIMRSRFLPKLPTKRSMRAPRMRWTSTLHARFVHAVEFLGGHERATPKSVLELMDVKDLTLAHVKSHLQGNRMDLARTTSSLRFAAQRISMPYAILSNFQSKVDRPIGLCNQRRTTLPVPLFGVTLQGRVFITLFLSLMLYIYMPELLNSA

>MDP0000179288 MdGARP30

MPLEGIFIEPSTTRTTSSQIPDLSLHISPPNTTSSNSSSIFNSTTTNEPQNPTTQAHTEL

SLGRCFTGGAHEQEPPQNPYQRQSFHLHSSNSHLSHLNHGVSLLDXSSSEGLRPIKGIPV

YHQNRSFPFLPMDNIITRDHHKDSKMCFYPRPPPPSFNHPSFPSAASHNTQSTPYFGGGG

ATGLEPMSRFNGFSSVDAFKSNNQLHHHHHLHHHNQYGIGGVXPISEGSHHGIMRSRFLP

KLPTKRSMRAPRMRWTSTLHGRFVHAVEHLGGHERATPKSVLELMNVKDLTLAHVKSHLQMYRTVKTTDKPAASSGQSDGSGEDDILSPISSATDHHGLHNTQQFPEQRGLCDQSVRPDVDCTSSTTLWSNSSR

>MDP0000179986 MdGARP31

MNEQKIDCQERIQQSHDYISKFVSTSYGYSXSTRHXLLSKTRIQWTQDLHEKFVECVNYL

GGAYKAPPKSILKLMNVDGLTIFHVKSHLQKFRNSEYPPGSAKGKXEKRTTSNVEPQLDV

ETXLHIKKAIQLELDVQQHLHEQLEIQRNLQLRIEEQGKQLKNIIELQLKTIIDLQLKNS

DTTFYEGAPLSSLDDEFIGLSINT

>MDP0000182154 MdGARP32

MKVGXPSAIMSHHGVISVPQSETTKGVTSSYCTSPSPIHDFLGSESEGRSFTASKCSSPR

VSPFKRAESLGXPTNMRGSPVQHSKSPFSRSSVFCTSLYQSSSSSSETSRQLGKFPFLPH

PPTYGQSNSAVDSKSPLLFSEDTSNQYDDEQSEDLMKDFLNLHGDASHGSFHDISCGTDA

LALTEQLELQFLSDQLDIAITDNGENPGLDEIYEIPQASPNTAIGLTXSKGSCVTAPLVD

ALSSHPSPGPASAHRPRMRWTPELHERFIEAVKRLDGAEKATPKGVLKAMNVEGLTIYHV

KSHLQKYRLARYMPEKKEDKKASNSEEKKATSTINESDGRRKGSIPITEALRLQMEVQKQ

LHEQLEVQRALQLRIEDHAKYLQKILENQQKAGSALLSPQVLSSLTTNSIQVSEQQPSSS

SCVSPPQLAESDSSSHLSLKHKATDCSDSELSAGTKRPRLEEKPDEGAVENPQL

>MDP0000184307 MdGARP33

MNEKKIDCQERNQQIHELIGECSFELGNQSCQIFGQQQQQAWNNMGIWVQQPTMDHEXSLLQNLGPPKTPSSIISRFESPVSAFYATELYMGGFPQYDSQAXQNPQFPSGQSNXESYSSM

NSSEQAPNFDIRNTLQSIGYCNSFTTXPVTQLSFFSGQGKQPPRISSGHVSDCYGDSPSS

SPVLSSKTRIRWNQDLHEKFVECVNRLGGADKATPKAILKMMGLDGLTIFHVKSHLQKYR

IAKYLPDPAEGKSEKRTTLNVEPQLNVKTLLQTYDVNISRSCLQIKEALQLQLDVQRXLH

EQLEMQTQRKLQLRIEEQGKQLRKMFNLQQQTTRTHLDTASVELLVQWKSQ

>MDP0000187636 MdGARP34

MMYHHQQQHQHQHQHQGKKIIHSSSASSSRISMPNISSERHLFLQQGDQNSPAGDSGLVL

STDAKPRLKWTPDLHERFIEAVNQLGGADKATPKTVMKLMGIPGLTLYHLKSHLQKYRLS

KNLHGGHVTSSLTKIGPVPVSVAGERLISEANGSQMSTMSTGIAPQSNKGLHISETLEMQ

IEVQRRLHEQLEVRACSWHHSVEHKLFPIIAALFLQFVPTENQVQRHLQLRIESQGKYLQ

SVLEKAQETLGRQNLGXVGLEAAQVQLSELVSTVSTQCLNSSFTELKELQGLCPQQTQMNQQPADCSMDSCLTFCEGSKKDQDQIHSSGVPLRPNYNGRVTAVLEQKDTERAQPPMLQNTSQLNWCEDMKENNMFISSISKDAEKRMFPAAETRSSDLSMSIGLQGERWNIDSKGRDSDDSFLERTNSSRADSAKVKGAKVSXGYRSVPYFAAKLDLNAHDDNDASSSCRQFDLNGFSWS

>MDP0000187812 MdGARP35

MMKNTSLPLPDLSLQISPPSSADDLPYDIGVTRKALYSTDRSSATDSGSSGSDLSQENGF

YHLERSSSYNLASADEEPKLSLGFDEMKDHHHHMASPLPPALQMPXNFNSNHYLNHHHHQPQIYGREFKRSARTVGGVKRSIRAPRMRWTTTLHAHFVHAVQLLGGHERATPKSVLELMNVKDLTLAHVKSHLQMYRTVKSTDNKGTGHEGQTDMDLNQNPGTKPALQINAQLSCEKANINRGSWPSSTEMNGSSNLRHGHGLAYTHSPPSNGTKVEKEDSGHHVYDQSMKERPMLDCGSLASSDMYLNLEFTLGRPSGEWTVLNSLNLQMT

>MDP0000192297 MdGARP36

MVGFGCSGGDEQDFQGSFPTPIVTRSDDRVVLEGGEAAVVEXVDRETRXLQLPQDCVLKAKLRPRLQWTPELHTRFVDAVNQLGGPHKSTPKKVQQAMGIQGITLFQVKSHLQKYRLGRYSGKEWTEGTRISKETSVSQDLEEGLSTSRTPLQLPQTAXMYDLLXSSIFHXNALVNLFGKAGNEKTSAEIENESAENHYPSSIFKCIILPSDELAGRQLQLWQDAEQRYMNFAMENAHKKLTEQFFGGAIAKGMLNGVSGLGTMELYPAYQMALGNSMQPSLEGHSTSHGHSEXSSVENFQTHTEDDEKTEQSLDDDPAEAYLIDPDPDME

>MDP0000196504 MdGARP37

MDELEFGYQEAEKSNEISEEQVENGSVGSSQKFSSFDLNEEASSDNEERGQGNKAADNDI

SSEGNEGRRVKVRQYVRSKMPRLRWTPQLHLSFVHAVERLGGQDRATPKLVLKLMNVRGLSISHVKSHLQMYRSKKLDGNGQVLSENHESKHGRDYISCMVHQTTNQPRRHVYFRMDNGGIVVATNPHDCHSQSQPDFKASSRSSSLLNKELAKGNNSNKSIDTILQTLPINPSKFLEER

PPFETINKQQWRAKRRPAAKINWSDNRHGSQSDHLMHHMHTTPRLIGESHNLRPPNWHLGESRNVRQFPSNTHNSITNSSCYKTEXKSPFRFELNEENFSKYKEWMPQLELGLGQRVENYEEKKILSRHRMXXESKPNQALKVAKKKASITAKEHIEDRHHKSKEAHRKQPTSNXGNKAKLRQEGGKNTRASKKSEALHISPPKRESQRFVFTVEGKGKEERGVEEGKPKAAVEEKTSMDKRKDKENKRSKGLPPTLAIARIGGSEEENXSGRCTHMHAVRRTFTKEMRTLTKEXKFRLTNFDIKIKIL

>MDP0000197814 MdGARP38

MASSNRLELTLSLKLANIENVSQKLSVLNEHLQKHQEELRKLEAFRTEMPHSVLLLSDAV

QVLKNEISELSDHQSERRSAFRKYKSSSPPFNPKPXGIDTTDHKQVMTSQCSQSSSRMGM

MVNKEPTSPTPFPCDLNMAADDFELESKPQAQPESQPQPQLPXPLPLPNLKNKRRSWSPE

LHXRFLKAMSLLGGPQEATPKQIQAVMQVDGLTHDQVKSHLQPYLGDGASNXRLRINTSHKMKYRLNNRGVQPASXVSGQRAPHFYRDYWQQQQQLEGASNGGVRVGIGSSNGGGRSRDISNKKAKTSR

>MDP0000200243 MdGARP39

MELLSLDSSPLFVPKTITDFLAQLAAIKDGSQRSSELDGYVKRLEDEFRKIEVFKRELPL

CMLLLKDAIELLKEEGMRWRRMEERPVVEVFIPLKGNSEEDGGLVSGKENIDKKNWMSSAQLWNTSINVFDYTKRDSVELKLTETLLGFAEISYYLGFFSTGNEEDDRSVTENPIEVSNN

RAVEGALLAFRGQNELSGFAKPCLKEVKEVSEVPNLCLMTPLMSEALXCSASANSRTSNG

CKGSGSGSGVAGQVKLLNKPQPQQQQQPLRKPRRCWSPELHRRFVDALQQLGGTEVATPKQIRELMQVEGLTNDEVKSHLQKYRLHIRKLPPSAAAGQGNALLMSLDHSGDHSKANNVQSGSPQGPLLAGGFAKCRSTTGGESGNSREAEEDEKSDG

>MDP0000202657 MdGARP40

MESTEEMQASSKLGFREYVKALEEERHKIQVFQRELPLCFELVTQAIERCKQPVSDTTAD

YMHGQSECSQQTSSEGHVFEEFIPLKRTSSSDSEDDEVHESQEAKDNEKDDKIAGGDKKK

SDWLRSVQLWNTTPDAPLKEELPRKALVMEVNRNGGAFQPFQKEKSIGKTXVPVAKQPSSAAATSSTTDTVSGGSGENNKKEEKDGLGQRKQRRNWSPELHRRFLNALQQLGGSHAATPKQIRELMKVDGLTNDEVKSHLQKYRLHTRRPTPTIRGNNNAAQAPQFVVVGGIWVPPQDYAAVAATTASGEAANNGIYAPVASTPAVTQVSPPVQRLRLKQPEPSHSDERVSHNEGRGHSNSTATSSSPHTPPSPAF

>MDP0000204785 MdGARP41

MASSNRLELTLSLKLANIENVSQKLSVLNEHLQKHQEELRKLEAFRTEMPHSVLLLSDAV

QVLKNEISELSDHQSERRSAFRKYKSSSPPFNPKPYGIDTTDHKQVMTSQCSQSSSRMGM

MVNKEPTSPTPFPCDLNMAADDFELESKPQAQPESQPQPQLPQPLPLPNLKNKRRSWSPE

LHARFLKAMSLLGGPQEATPKQIQAVMQVDGLTHDQVKSHLQPYLGDGASNRRLRINTSHKMKYRLNNRGVQPASXXSGQRAPHFYRDYWQQQQQLEGAXNGGVRVGXGSSNGGGRSRDISNKKAKTSR

>MDP0000214282 MdGARP42

MDELEFGYQEAEKSNEISEEQVENGSVGSSQKFSSFDLNEEASSXNEERGQGNKAXDNDI

SSEGNEGRRVKVRQYVRSKMPRLRWTPQLHLSFVHAVERLGGQDRATPKLVLKLMNVRGLSISHVKSHLQMYRSKKLDGNGQVLSENHESKHGRDYISCMVHQTTNQPRRHVYFRMDNGGIVVATNPHDCHSQSQPDFKASSRSSSLLNKELAKGNNSNKSIDTILQTLPINPSKFLEER

PPFETINKQQWRAKRRPAAKINWSDNRHGSQSDHLMHHMHTTPRLIGESHNLRPPNWHLGESRNVRQFXSNTHNSITNSSCYKTEXKSPFRFELNEENFSKYKEWMPQLELGLGQRVENYEEKKIXSRHRMXXESKPNQALKVAKKKASITAKEHIEDRHHKSKEAHRKQPTSNXGNKAKLRQEGGKNTRASKKSEALHISPPKRESXRFXFTVEGKGKEERGVEEGKPKAAVEEKTSMDKRKDKENKRSKGLPPTLAIARIGGSEEENXSGRCTHMHAVRRTFTKEMRTLTKERKFRLTNFDIKIKIL

>MDP0000217637 MdGARP43

MKDQAMKRVEGDDHHHQRSSSLKRSPSSSVDNGDQKPYTSSFDDGDGDGVTPMRSTTLGKQLVVDGYPHHFPAAPSHRQMSGSLLKPSSXSSVPVRPYVRSKMPRLRWTPDLHRCFVHAVERLGGEQRATPKMVLPIMNVKDLTISHVKSHLQVCDRGQLYRNMKHEQGIQAAAMAAKENGVFLVSEHSNNSIYLKXINIAQXVCHQKNXGVIKMYNYGPCQSNETCYIEDLATKNTSPQAKWKDETKPLVPYQVSSKRYEQKPNSFIIFNXLLKRCSDNIGQESNHKHLEDLRGTESEYDARMFLSLNSSSTKAXRELLRLSKASGNSDANDVSLELTLNT

>MDP0000221436 MdGARP44

MMKKISLPLPDLSLQISPPSSADDLPYDIGVTRKALYSTDRSSATDSGSSGSDLSQEHGF

YHLERSGSYNLASAVEEPKLSLGFDERKDHHHHMASPPPPALQMPRNFNSNHCFNHHHPQIYGREFKRSARTVGVVKRSIRAPRMRWTTTLHAHFVHAVQLLGGHERATPKSVLELMNVKDLTLAHVKSHLQWGVENKRVRTKKHLEGMYRTVKSTDNKGTGHEGQTEIGLSQNPEINPALQANGQLSCDKVNTNREIFIAEVSKHLEFDEHGVNWLGLLWEYMIPMCDQKSRKFTANLNAVEIQRGLRSSSTEITTGSSNLRHEDGLTYSHSPPSNGTKVEEDDSGRRHGYAQSMKERPTLDCSSLASSDMYLNLEFTLGRPSGEWTLLNSLNLQMT

>MDP0000222377 MdGARP45

MEDECPTKNLEFLVESCAGGGGHHGKIILSSVVSLTSTFWEATLPSPSIPWPFPDSSRPP

TQWCPPNDPYIKLNVDASWNRADEAMTVLRGSGKFNLGKNFKYGNFFLGLLLVLDSKIGQPDGRPFGVEEEHEDVQQNLVANLKRFCLSKEVEIGYTAATCGVVVEQLLDKNPYNSDIFSDLENYVNEQVCFFTNRNLGLMPISAFFASIRVENYNFENKFANWLPVASRGHEIMADYVQEYSSYVGFMRDGAQWFVENTDIKLVGIDYLSAAAYDDLIPSHLVFLEGRENDDPLQGEIAWNRTPRELGISAYALQQLGGSHAATPKQIRELMKVDGLTNDEVKSHLQV

>MDP0000225179 MdGARP46

MAESEATSKNQLQSEEHREMICIKGVAILVGAPWLSTVVKYGRWGPKSRGEEGWVGGVEEGGGMVDVRGVEEERKVGWVGAEERRKEEGRKGGEGELLRWSSTYWLAVNPRGNNCLMLILSPLREGHHHHQLKDEKQLGDDGDVESSFAFNGGNNAVLDFPEFSGGMGMGSLLDSIDFDDFFIGIHDGDVLPDLEMDSEILDFSISAPAGDTNTTTTTPSKEEEEVADGYYNTAKTTTTLSKVEEPEEEDMQGHDEVAAAAVGITSTNNNSGLNQNYSSSSTTTTPTPDHRVDQEIASKRYDTNEIRAAHRPSSGSTRRESADQKLATKSSSTVQSKNSHGRRKVKVDWTPELHRRFVQAVEQLGVDKAVPSRILELMGIDCLTRHNIASHLQKYRSHRKHLLAREADAASWTQRRQMYGAAAAAGGGAGAKSRRDVMMMNSPNWLNAPTMGFPPITSTTPPPMHHAQHHHQLTGPLHVWGHPTMDQSMMXMCPPKHHLPHHFPSPVLPPPPTAPAHTWPPPLPSPPDASYWHHPHSHHQRNHIWGKYGWEGVGQVWVGMMGGGCRGGAAAVPDGLTPGTPCFPRQLAAPTRFPAPPVPGIPPHAMYKVDHAGIAVPTPPQSGPHPRLDFHPSKESIDAAIGDVLSKPWLPLPLGLRPPTTDTVMVELQRQGVPKIPLSCA

>MDP0000229587 MdGARP47

MTSHDRPMCVQGDSGLVLTTDPKPRLRWTVELHERFVDAVTQLGGPDKATPKTIMRVMGVKGLTLYHLKSHLQKFRLGKQPHKEFNDHSIKDHASPLDLQRNSASSSAIIGRSMNEMQMEVQRRLHEQLEVQKHLQLRIEAQGKYMQSILEKACQTLAGENSMAAAAAGSYKGNIGNQGVNPTDMGALKDFNSSLNFPSFQDLNIYGGDHLDLQQNLDRPPLDHSFMPTNDNNICLGSKKRPSPYSGSGKSPLLWSDDLRLQDLGTAASCLGPQDDPFKSSDSIQIAPPSIERGSEIDSI

SDIYESKPMLQGDDKKFDSANTLERPSPRRAPLGSDRMNPMINSGIRQGRNSPFGSAQNQ

Y

>MDP0000231315 MdGARP48

MASSPSELSLDCKPHSYSMLLKSFGDLQASHLHDQSQTQKLEEFLSRLEEERLKIDAFKR

ELPLSMQLLTTAVEASRQQLQAYRTNNIQGQNRPVLEEFIPLKHSNSEGSEIPTNATSDK

ANWMTSAQLWSQATDHNPNATSKPQSTITTGPKETDIGFXVSPKLGLETKQHRSYGGGAF

LPFSKDRSPTXRPLPELALASPENNKSEIVLEDKTNHHENGNINSSSAAGNCNEQGKLLG

AGGINNSSEGQNGQTTTTAGAGATTTPTQTHRKARRCWSPDLHRRFVNALQMLGGSQVATPKQIRELMKVDGLTNDEVKSHLQAKMQARKTYACLPFVLFTSXLPFLIVLKDKRGQLLELEIQCHLHPKNPNLFSNKKNFYHSSCIGKEEKYRLHTRRPSPSPHAGGGPTPQLVVLGGIW

VPPEYATAAHNGSAALYSPHPASHAPPHYCATPMPQDFYATQQQQQQLHHHTLQHQLHVYRTSTASHTQSSPESDGRGAGDRSESIEDGKSESGSWKGTESGDHVNMNGGDQRNGGLTARRSEDGEDSNGSQITLKF

>MDP0000232923 MdGARP49

MYHAKKFSTASLVPHKPQSSQELANVGVVSGGSSVKSPTPSGGGSGKQRLRWTSDLHDRFVDAITQLGGPDRATPKGVLRVMGVPGLTIYHVKSHLQKYRLAKYLPESPADGSKDEKKGSGDSLSCSDSSPGVQINEALRMQMEVQKRLHEQLELIQQLHSLEVKVFVXHFSSPGPEFGS

GFGNMDWVQRQLQMRIEAQGKYLQKIIEEQQKFGGSLKASEALPSAEEKQKPAHLETMGDASAAPSSPRKKQRVDEGLADGCATSNLPLKADQKNEFVGQWDREVYGSDGGYGFGLQTEFKERDGGAAQKAPMELDPLCGSKQ

>MDP0000234120 MdGARP50

MITRYKRNGYVESFSKHAKFVIELGLGEHGHPEEKFFARTNHPSALPALAKYSIQHNNSS

PTPNQELEIMREKEKGQGEAVVASSLLVWYRGKELERRRWVQGRCTRDKQKAQAAGGERERGKYRIVKSTFIKGSGEDPGWWEKKMTTSSCSARNGAVRQYVRSKVPRLRWTPELHRCFLQAIERLGGHKTEATPKLVLQLMDVKGLTISHVKSHLQMYRSMRGDPIIRQDRVQTRKLHSFEEAEDDGCVEEVNGLSFYPSSKPTRESDSQVICNPRRSKRARTETMGSNISEGLQQQQCSKGGIYETVSNPYSFDDYVLALGIKENPHPSAFKIALQESDFLKVATLPEAGGAHVEDHHEAGQCELSLSLSLHQPSSHKSNASSSDFSGAISSSYSRPNSKDCSASSSGNRNLNLNLSI

ALCGN

>MDP0000241055 MdGARP51

MEMASSPRTVEEIFKDYSARRTAVVRALTYDVDEFYGLCDPEKENLCLYGHPNETWEVTL

PAEEVPPELPEPALGINFARDGMNRKDWLSLVAVHSDSWLLSVAFYFGARLNRSERKRLF

SLINDLPTVFEVVTERKPVKEKPSVDSGSKSRGSTKRSGDGLVKSTPKLPPDESFEEDDD

EHSETLCGSCGGNYNADEFWIGCDICEKWFHGKCVKITPAKAENIKQYKCPSCSLKRGRH

FIRRLETASKRSTLHLVHIVFCLXLLVLTTVGASCTCYSFCCNRVPPLHRAQEHLLIFDE

FEMRLSRSASSAANKERLRWTQELHDRFVEAVTKLGGPDRATPKGILKAMSVSGLTIYHI

KSHLQKYRISKFIPESTSKGKLQKKNISEMLPNFGTTSAAQLNEAFKIMQVQVNRRLSDQ

HEVQKSLKQKFEAQGRFLERYSAEHHXTNKNRPILITKPKKAPLSQTLLPSLCDDSESNA

KDFXSDSEPDRSETETSAEQFRTXKKKLRLHHHQNDHNVQLALNSEFCYIPHEDDHIRFP

WNFAACSSPLPLVVXSCFL

>MDP0000244794 MdGARP52

MHTNSITMSKKEIMKGSSHRIGVRKYHKSELPRLRWTSQLHELFVEAVQSLGGKYKATPK

RILQMMSVKGLEISHVKSHLQMYRSFKDNNINIFSEDHSSSVFSAFPSQTHQRSSENYVR

YDELKDMENVMHSESDEEAAGKQHAIHQNTHTSELSRLSMDDQTCQLYGLCELSLSFDPVTNRNQRERQFRPSADEHSSSESTSTGNFSNIQVAGNHINLDLTI

>MDP0000248355 MdGARP53

MMGSXGGSECSKTSPSDKQNEDGSESGENYGNGGSSKPKNNGGSSSNSTVEEGDQKKVSVRPYVRSKMPRLRWTXXLHLRFIHAMERLGGQDRATPKLVLQLMNIKGLSIAHVKSHLQMYRSKKINDAGQVLPDRQGHLGECGDKNIYNLSQLPMLQGYKLYNRRHTTSFRYGSYGDANSWNAFENPRFSRSSTGFQGNWSTSSTSNNAYNYLTSCSFGDHPQSSWITRTLKEDCQLYNIRESLKPGELITSFNTNHGPFQDFDTVTKNLVQDQLKTLKRKASDCHDLDLDLSLRLTSRKNDEDHTHEVDSSLSLCLYSPPTSSKLRSLKE

>MDP0000253656 MdGARP54

MQVRGERSSPIGHVPCITKRKDSESRNGTFQHGSKEAHVGKQACARTNQETNLEEREKSMSIPILEACKKMKRSFELGNQSCQIFGQQQQQAWNNMGIWVQQPTMDHEGSLLQNLGPPKTPSSIISRFESPVSAFYATELYMGGFPQYDSQACQNPQFPSGQSNNESYSSMNSSEQAPNF

DIRNTLQSIGYCNSFTTSPVTQLSFFSGQGKQPPRISSGHVSDCYGDSPSSSPVLSSKTR

IRWNQDLHEKFVECVNRLGGADKATPKAILKMMGLDGLTIFHVKSHLQKYRIAKYLPDPA

EGKSEKRTTLNVEPQLNVKTLLQTYDVNISRSCLQIKEALQLQLDVQRCLHEQLEIEEDI

EENEIVVPVESPTLIGEDVLKEEALQVVAIGCNWEPKRR

>MDP0000253782 MdGARP55

MLREKGLSSEAAAGGRSCLALNPIPDLSLHISPPNTNTNSAPSSICTDQVTPDHDSSSCF

DIWRRDTDEDCTNXGALSNKSHSDSCIRASYTSSSPAAAGADTELSLANPSALEAESAWK

KNYFGGGDGYNDEAKNSGNSVTSSNGISMLERLKPIKGIPVYSTSSTNCSFPPSFSPNPV

YQTAPYLSNSFGPAAPARNCSGRGMGMGLGGFNGITMDSLSLGVGGVQQQQQQRQRQHHFPSYNLNPYYPQQLQQHHCVGGGGNYNADLSSNGFTMRSKFTPPKLIGQNINKRNMRAPRMRWTTSLHARFVHAVELLGGHERATPKSVLELMDVKDLTLAHVKSHLQMYRTVKNTDKPLASSGYQRPDSTQAFLFQDKTSDPNLIVVAFRSTEAWRTXLGLGISRAPTELETSPIQAIQKTKMKPRRIGSRIVPIGGREKAEVGRKVA

>MDP0000262747 MdGARP56

MKNKFEASSSRNMDRYVEETWEEFEQVENFYTDAAHEVTNERFGAISEYRNSRSVKRFEHRSYPEETIYQRRTTTTVTRPTTTTVTRPCERLSWTPDLHRKFMRAVEILGGEDYATPKNI

QSLMRIPEITLPQIKSRLQLVRRRSRSCYPDYGTSFLPEDWDKFLVKYLYPSFTPTVGLF

VAGSVGYGVVKYLQNEELKGRENETGNWTVYVYANDLEKGDRRPWLPCTKDARRCRYSHLE

>MDP0000263292 MdGARP57

MMKNTSLPLPDLSLQISPPSSADDLPYDIGVTRKALYSTDRSSATDSGSSGSDLSQENGF

YHLERSSSYNLASADEEPKLSLGFDEMKDHHHHMASPLPPALQMPXNFNSNHYLNHHHHQPQIYGREFKRSARTVGGVKRSIRAPRMRWTTTLHAHFVHAVQLLGGHERATPKSVLELMNVKDLTLAHVKSHLQMYRTVKSTDNKGTGHEGQTDMDLNQNPGTKPALQINAQLSCEKANINRGSWPSSTEMNGSSNLRHGHGLAYTHSPPSNGTKVEKEDSGHHVYDQSMKERPMLDCGSLASSDMYLNLEFTLGRPSGEWTVLNSLNLQMT

>MDP0000265555 MdGARP58

MSSVPPELSLDFRPTFVPKTISDFLKEVSMIGNVSERMSKLDDFVNRLEDEMRKIDAFKR

ELPLCMFLLNDAIRALKEEAMQCTAPDIQPVLEEFIPMKNDCGKNKGGSSNNNKKEKDSR

DKKNWMSSVQLWNTDNYQQHPSSDFPYDQKRVSEIVSKRNEAENEDPFQTCRNRTGGKAFMPFKGYPAFSVTPVRLEEKEELPVRGLSLLTPGIKNPKEESTSSGSRSTCGRSTSFSTAN

AQSNMRTLPQQPTSRKQRRCWSPELHRTFVNTLQQLGGSQVSYLRFYDLAVATPKQIREL

MQVDGLTNDEVKSHLQKYRLHTRRVPGASTAAPKNQAVVLGGLWTSHDRYTDSSKASSSQSGSPQGPLQLTGTGGDDEDDEDAKSESHSWKGHIHKPGKDDV

>MDP0000266040 MdGARP59

MSAPEKTDHHRSITGDSGASVSDDSDDNQSWHSMSDSXNVXRKSCGSDCSVNEVDLESGLLEVKKKHSSREASSRPRLPPSFSGPRQSSSDDRQLVEIIPVVIYSVERLGGDQRATPKMV

LQTMNVKGLNISHVKXHLQMYTSMKHEQGIQAALAAKKNESEMRQWKREGEYRQVLDRAPSSEWWRRRGMAPIELMSKSHAATSDQSSHQGSNTYVAPSPTLSQSLSFKDSVETVRNVFGRWRKSVGEPTKKAEDLAGNTWQLLKTSPSFADAAMGRIALGTKVLAEGGYEKIFRSTFKTTPEEQLQNSFACYLSTSVQSWEFYMFLLQSLRIAVIIPFRTKLRAKSSSWSSVSLHNLQLHHHHSPENNAVMGYLQQPTWTKGITFRMC

>MDP0000269857 MdGARP60

MVGFGCGGDDEQDFQGSSPTPTVTHSSDSVVVEGEEAAAVAEEEEEVDRETCDLQLPQDRVLAAKLRPRLRWTPELHACFVDAVNQLGGLHKATPKKIQEAMGVQGITLFHLKSHLQKYRLGRHGVKEWREGTQTCGNKRMSAEIENESAENHYPLQKLPLQRLRFQVHYLVNAVMFYVLGCGSMHRHSYKLVKEALDEERDSMQVEASFRVRKLIWELXLINVGRRLHIRQDAERRFMNFAMENACKKLADQFIGAAXAAAILNGIAGVGTLELYPAYQMTEGTGMQPSLEGHXTSHGHSENSSVEDFHTRAEDDEKTEESLDNDPAEAYLINPDPDME

>MDP0000272003 MdGARP61

MMGSQGGSECSKTSPSDKQNEDGSESGENYSNGENSKPKNNGGSSSNSTVEESDQKKTSVRPYVRSKMPRLRWTPDLHLRFIHAVERLGGQDRATPKLVLQLMNIKGLNIAHVKSHLQMYRSKKIDDAGQVLADHQGHLVECGDKNIYNLSQLPMLQGYNRSHTTSFRYGSYGDTNSWTALENPRFSRSSTGFQGNWSTSSTGNNAYNYLTSCSFGDHPQSSWITRTLKEECQLHNIRESLKPKEFITSFNTNRGPFRDSDTISKNLVQDQSKMLKRKASDCDDLDLDLSLRLTSKKNDEDHTHEVDSNLSLCLYSPPTSSKLRRLKE

>MDP0000274727 MdGARP62

MMGSXGGSECSKTSPSDKQNEDGSESGENYGNGGSSKPKNNGGSSSNSTVEEGDQKKVSVRPYVRSKMPRLRWTXXLHLRFIHAMERLGGQDRATPKLVLQLMNIKGLSIAHVKSHLQMYRSKKINDAGQVLPDRQGHLGECGDKNIYNLSQLPMLQGYKLYNRRHTTSFRYGSYGDANSWNAFENPRFSRSSTGFQGNWSTSSTSNNAYNYLTSCSFGDHPQSSWITRTLKEDCQLYNIRESLKPGELITSFNTNHGPFQDFDTVTKNLVQDQLKTLKRKASDCHDLDLDLSLRLTSRKNDEDHTHEVDSSLSLCLYSPPTSSKLRSLKE

>MDP0000280005 MdGARP63

MNEKKTDCQEINQQSHELISECNFEVGNQSYQIFGQQQQQAWNNMGIWVQQPTMDHGVSLLQNLGPPDPKSPSSITSRFESLASTFYATERCMGGFPQYDSQVGNNPHFPSGQSYNESYS

SMNSSEQTDLDFDIRNTLQSIVKTQPRSYQYHKSSEKFNQIPGSDLSGRKQSPRYSSGNV

STAYGNSPSTSPVLSSKTRIRWNQDLHEKFIECVNRLGGAEKATPKAILKMMCLDGLTIF

HVKSHLQKYRIAKYLPYPAEGKSEKRTTLNLEPHLDVKTGLQIKEALQLQLDVQRRLHEQ

LEIQRKLRLRIEEQGKQFKEMFDLQQQTSSSLLKTQNADVTCHEAGPSNSLDDTDQVSTS

EVKSXIYPSVRVSAILERKGTGGTLFLXLSGGSLEFSISLKNFVDHLTQELKLWKIRNKL

IWLCWRLKKIASYMWVAQRPPGYAFVDFDDSRDADDAIRELDAVIDNYKQENITTVNARKARAFRCVLQGIAETGKAYVHDFLYVNGRPVLXVDASKHLSAVHDPAENEKLCVFLIEKALGACPEGIEEILGIFNLHWVQQRFFYELIRIAYFVYKCSA

>MDP0000284686 MdGARP64

EQLPSLILQLXNXRGLSISHVKSHLQMYRSKKLDGNGQVLSEKHESKRGRDNISCKVHQT

INQPCRHVYFGMDNGSIVVTTSPHDCQSQPQPDFKAVSRSSSXLNEELVQGNNSNKSMDT

ILRTVPINPSRLLEDKRWPPSETINQQQWRAKRIPAAKISWSDDCNGSQSDHLMHHMHTT

PRLIRESHNIRPPNWNLGESRNIRQFPSNSHNSITNSSCYKTEFEPPFGFELNEEKSSKY

KGWMPQLELCLSQSVENYEETKIISRHRMNRIIQMRTKSMNTSRGDLSIVDYLDKVNVIV

DNLAVSRAPFFESDLVAIIMSKVGPHPLCGHLSSSNGILGPGSSGFTLASPTFSSFGRIQ

CQICQRYGHSAIDCFNRLNMSYEGRVPSSHHQAYVVVPSCRASNTASAAPSVQQWLFDSS

ANSHITNDVGQLHNPREYHGTDQIRGVHVGPGFADGDDHFTRPE

>MDP0000285150 MdGARP65

MAKLVGFLVFFTAFVVAAADWNILKTRQTDNVELGDELKKYCESWRINVEVNNIRGFEVVPQECVWYVKKYMTSSQYKADSQKALEEAWMREGKAPALEHTLKLFHEIKDRGIKIFLISSRREDLRSSSVDNLIRVGYHGWTGFILRGLEDEFKEVQKYKSDARRRLXDEGYRIWGIIGDQWSSFEGLPMAKRTFKLPNSMRKLMTPFSGFAVNNKQKMEARPAMSIRTSAAKQLTHMGVSAAMSSSLPVLPTSLEETYPKLPDSQQVSRERELMTRPVGHAGXLTSNSGVVGHIFSSSSGFSTDLHYSALSPHEKQSKNSPFISQSSAALPLPHSPHSGFLQSTESCPYPKENSGSWCT

DSLPGFLHFPVNSHVENSQIESSSCSGIMXADEFVKRHDWQDWADQLITDDDALTSNWNE

LLDDNSVTDLEQKMKYQAPQPSSTFSAEQSQVHQQQPASSGEIIPAPSREIISVPATSSA

NSAPAKARMRWTPELHEAFVEAVNQLGGSERATPKGVLKLMKVEGLTIYHVKSHLQKYRTARYRPESSEGASEKKLTSIEEMTSLDLKTGIEITEALRLQMEVQKRLHEQLEIQRNLQLR

IEEQGKYLQIMFEKQCKSGIDTLKRSSSNLDDPSAQPSDEMQVCPDKSELDSSKLDQGET

ETDPVKANPTSSGGSQEPEGKQKSPEAEALPENLEPDGGXSSPQPPKRAKIKE

>MDP0000288143 MdGARP66

MAFDPLLFCEEGFEEYLGDNGSEEESENCDGFSKKQSSFPLIFLESDMFWENDELSSLIS

KEEQTHVHFSGEISDRSLMAAWNEVIKWILSVKAHYGFSSLTAILAVNYFDRQAVDELAH

RRRMRLVGRQSGGDPYASSLRFARDSGLVLSTDAKPRLKWTLDLHERFIEAVNQLGGADK

ATPKTVMKLMGIPGLTLYHLKSHLQKYRLSKNLHGGHVTSGPTKIGTGTVPVSEAGERLI

SEANGSQMSTMSIGIAPQSNKGLHLNETLEMQIEVQRRLHEQLEVQRHLQLRIEAQGKYL

QSVLEKAQETLGRQNLGTVGLEAAKVQLSELVSKVSLNSAFTELKELQGLCPQKTQTNQQPTDCSIDSCLTSCEGLRRDQDQIHSSGMPLRPNYSGRAAALLEHKEAAQDQXPMLQNTQLNLCDDLKENMXLSSISKDAEKRMFPAATRSSDLSMSIGLQGENWNIDSKGRDTDGSFLGRTNSRADSAKVEGAKVSQGYRSVPYFAAKLDLNAXDDNDTSSSCRQFDLNGFSWS

>MDP0000290477 MdGARP67

MVDFDALSRPLRSRLKHNSREASSRPRLPPSFSGPRQSLSDDRQLVEIIPVVIYSVERLG

GDQRATPKMVLQTMNVKGLNISHVKSHLQMYTSMKHEQGIQAALAAKKNESEMRQWKREGEYRQVLDRAPSSEWWRRRGMAPIELMSKSHAATSDQSSHQGSNTYVAPSPTLSQSLSFKDSVETVRNVFGRWRKSVGEPTKKAEDLAGNTWQLLKTSPSFADAAMGRIALGTKLLAEGGYEKIFRSTFKTTLEEQLQNSFACYLSTSVQSWEFYMFLLQSLRIAVIIPFRTKLRAKSSSWSSVSLHNLQLHHHHSPENNAVMAAKWSRDHDDKGIQTSNDAKLLPSHYKVPASPYVSTLGRWIDKTPSTIIPKKSGHFRKRKQKEKKKETPSSKNRQKQSSTSSGTGTTNGSSTNSNHALTHAVNM

>MDP0000303924 MdGARP68

MTLQLWGPKSALKGLSTLRVENYNFXNKFXNWLPVASRGHEIMAXYVQEYSSYVGFMRDGAQWFVENTDIKLVGNDDPLQGEIXWNRTPRELGISAYALQQLGGSHAATPKQIRELMKVDGLTNDEVKNHLQV

>MDP0000312944 MdGARP69

MERIEGDDHHHQSSSFLKRSPCSSVDNGDQKPHTSSFDDGDGVTPNRSTAQGKHLVVHGYHHHFPAAAGHRQMIGSLLKSSPSSSTPVRPYVRSRMPRLRWTPDLHRCFVHAVERLGGEQRATPKMVLQIMNVKGLNISHVKSHLQVFDYGQMYRSMKHEQGIQAAALAAKKNGMLQVSEYSNNSNFLKPIDIAQPVYHQKKDLMKKMYNHRPCQSNETCYIEGLDRNTSPLAKWKDETKPLVLPYQAPSSFIVDNDLLKRCTDNIGQESNHKELEDLGSTKSEYDARMSLSVNASSSKASRELLRFSKAAGNSDANDVSLELTLGT

>MDP0000314088 MdGARP70

MATPKLMPFFILVVFHTMLSSSSGSNSDLFYPSAKGIRAAYFPSFNSFSPSSIDTQYFTH

IYYAFLVPEPXTYKLNVTEFDQARIPEFIRALRTKKPAVTTLLSIGGGGSNSTTFALMAS

TQATREAFIRSTIQVARKYGFQGLDLDWEFPEDAVEMSNLGLLFEQWRKALDNEARVTGK

PRLLLTAAVYYAWKFTVYGGPRSYPAAAITKYLDWASPMCFDYHGSWDDFTGMNAALYDSKSNISTHYGIGSWIEAGVPPKKLVMGLPLYGKTWTLKDPKVNGIGAPASGVGPGDGILVYRQIVDFNSRTNATVVFDVESASYYSLSGSTWVGYDGVRSANVKVRFAKSLGLGGYFFWALGQDNGWDLSKQGTAGLGSVVGTRNPVQNQKDLWSLQLHREFVAAVNKFGAMTILIKNANPNLDGEELKSHLQKYCLYLEKSKEPQHPNMSAGVAFSSPSNLRSPNFTRQDTARHLRNSQDLRFASSSPSLRVAPRPSSFPKDNQQQSQLINDLNI

>MDP0000320628 MdGARP71

MVLQNMQNQNMXLVLSTDAKPRLKWTPELHQRFVEAVNQLGGADKATPKSLMRMMGIHGLTLYHLKSHLQAFSLSSFVTSNLCPDHDRKSKYRLGKSQQSENCADXRQEDYKELQSSDGHFGADISDEDHSQINESLQIARSLQLQMEVQRQLHEQIEVQRHLQLRIEAQGKYLQSVLKKAQETLSGYTSSSVGVELAKAELTQLVSMVNNGCPSSSFSELTETGTPTLKDVERKQMRGSMESSLTSSERSGREDEKLPENSNATCVELPLMDXHPDNKAWNNVANNHVFGRKRSXSPISEGVSVEQPVAKRTQTQXDKGGNNLRKSGLLATIDLNXKYQSDNIDSGPKAIDLNCKGI

>MDP0000321461 MdGARP72

MAIGEKKDIKSKXKNNLRDXNLKQPLVIKKXKIIAHEDSDQGSXKSGSIGMVLVSTFVAV

CGSFEFGSCVGYSAPTQSAIREDLNLSLAQMEARPAMSIRTSAAKQLTHMGVSAAMSSSL

PVLPTSLEETYPKLPDSQQVSRERELMTRPVGHAGRLTSNSGVVGHIFSSSSGFSTDLHY

SALSPHEKQSKNSPFISQSSAALPLPHSPHSGFLQSTESCPYPKENSGSWCTDSLPGFLH

FPVNSHVENSQIESSSCSGIMSADEFVKRHDWQDWADQLITDDDALTSNWNELLDDNSVT

DLEQKMKYQAPQPSSTFSAEQSQVHQQQPASSGEIIPAPSREIISVPATSSANSAPAKAR

MRWTPELHEAFVEAVNQLGGSERATPKGVLKLMKVEGLTIYHVKSHLQKYRTARYRPESS

EX

>MDP0000323119 MdGARP73

MDLVSGGNSLNNSNLASKQRLRWTHELHERFVDAVAQLGGPDRATPKGVLRVMGVQGLTIYHVKSHLQKYRLAKYLPDSSSDGKKADKKEPGDVLSNLDGSPGMQITEALKLQMEVQKRLHEQLEVQRQLQLRIEAQGKYLKKIIEEQQRLSGVLSEAPGSGHLARLSSDNCPESDNKTDPATPAPTSECPLQDKAAKECAPAKSISIDESFSSHREPLTPDSGCHVGSPAESPKAERLT

KKLRVNIGEAFTDPEAVLTHQILESSLNSSYQQGHTAFLPREQFHPSSGISFRNENQLEKDAGSDMX

>MDP0000323121 MdGARP74

MYHAKKFSTASLVPHKPQSSEELAIVGAVSGGSSAKSPTPSGGGSGKQRLRWTSDLHDRF

VDAISQLGGPDRATPKGVLRVMGVPGLTIYHVKSHLQKYRLAKYLPESPADGSKDEKKGS

GDSLSCSDSSPGVQINEALRMQMEVQKRLHEQLEVQRQLQMRIEAQGKYLQKIIEEQQKL

GGALKASDALPSAEDKQKPAQFETKGDAFATPSSPHKKQRVDDGLPDSCATSNLPLKADQ

KNEFVGQWDRDLYGSDGGYGFGLQTEFKERDGGAEQKSPMELDALCGSKQX

>MDP0000333691 MdGARP75

AVELLGGHERATPKSVLELMDVKDLTLAHVKSHLQMYRTVKTTDKAAASSGQSDVYENASSGDTTEDIMYELQNPRRSCEQLPSPQQGSRSTTVQNNKEYYGLWSNSSSREAWLHANPNDSASANIITSLDDQEREDLNMMDPKGLSYERISDVSSSSTNISAGGTSLSPRKPNLEFTLG

RSHX

>MDP0000450214 MdGARP76

MTSSSCSARNGAVGQYVRSKVPSLRWTPKLHRCFLQAIERLGGHKRNKLEELKNMVYLRGTLHISKLENAVNAGEERNDSKSGHKSNQMELQLVHALELHPLLXMEQLGEARVSLSGAFRIFVTYGELAQPSKEIKGRSFKVPFKEKIKPFLKGDTLFKTKVVLQEIESKSLCFVFKGNP

RDLPSTGLFSAKPTIVTTTNVKHAIPKQKPYEARIWEREQDLGRGVKHFLKTQKTLIWKW

QGFPPISKPLQVARRGLIITCPTSLARQDLPDKPASKAGARQKPSTGANLSGSCVLVGQE

ASPTEIPATNIVLARPVGFC

>MDP0000504079 MdGARP77

MELLSLDSSPLFVPKTITDFLAQLSAIKDGSQRSSELDGNVKRLEDELRKIEVFKRELPL

CMLLLRDVIERLKEEGMRWRRXEERPVMAEFIPWKGNSEEDGGAVSGKENIDKKNWMSSAQLWSTSINVFDYTKHDSELKPGNEEDDRSAAENPIELSNKKGVGGALLAFKGQNELSGFANPRLKGDKEVTVVPNLCLMTPLMSEALAGSANANSKTNHGCRGGGSGSGMAGQLKLQNKPQQQPQQQQQQPLRKQRRCWSAELHRRFVDALQQLGGTQVATPKQIRELMQVEGLTNDEVKSHLQKYRLHIRKLPPSSAAGQGNDLLVPXDHSGEHTKANNAQSGSPQGPLLAGGFAKGRSTTGGESGDSREGEEDEKSDGQMLKIIQFEIRCNKRSQLVQVKNCVAAWPTNRLTEDCLQIPSLPPVRPLLSSSGTMREIVTLQVGSFANFVGSHFWNFQDELLGLAEDPNSDPVFKNQSLNMDVLYRSGETHQGTVTYTPRLVSVDLQGSLGSMSTRGTLYDESSSASSNIYTWGGNVTTQVAEPHKKNLFLXSLYEEEXVNLLTLGNGVNAGGKSCRGEIHDSDKVESLENGVQYWTDFSKVHYHPQSLYELSGLWVDPQKFDNYGIGRESLSAGLQGEEVSERLRFFVEECDHIQGFQFIVDDSGGFSPLAVDVLESIADEYTNAPVLLYAVRGPGSSMDPRSQKQRVSRKLHDAVSFSKLSSLCKLIVPVGLPSLSTSKKSCTS

>MDP0000526864 MdGARP78

MNHNAAARFTIRQGEIAKPQINQPHPRHIPFELSGWQSLPGLLLGPSLSLGLAYAGPYPS

AYLALFHPQFTGEGHHHHQLKDEKQLGDDGDVESSFAFNGGNNTVLDFPEFSGGMGMGNLLDSIDFDDLFXGIHDGDVLPDLEMDSEILDFSVPAPADDINTKTTPPPPSKEEEEVADDY

YNTTKTRTTSSKVEDQEEEDLHGHGEVAAAAVSITSTNNNSGLNQNYSSSSTTTTPDHRG

DQEIVSKRDDINQIRAAHRPSSGSTRRKSADHKLATKSSPAAQSKKSHGKRKVKVDWTPE

LHRRFVQAVEQLGVDKAVPSRILELMGIDCLTRHNIASHLQKYRSHRKHLLAREAEAASW

TQRRQMYGAVAAAGGGAGAKSRRDXMMMNNPNWLNAPTMGFPSITSTTPPPMHHAQHYHQLIRPLHVWGHPTMDQSMMHTWPPKHHLPHHFPSPVLPPPPPAPAHAWPPGPPPPPDASYWHHPHSHHQRALDSPTPGAPCFPRQQLXAPTQRFPTPPVPGIPPHAMYKVEPAGIAAPTPPQSGPHLLLDFHPSKESIDAAIGDVLSKPWLPLPLGLRPPATDTVMVELQRQGVQKFPPSCX

>MDP0000609912 MdGARP79

MFQHQHHKKPSTMNSHDRPMCVQGDSGLVLTTDPKPRLRWTVELHERFVDAVTQLGGPDKATPKTIMRVMGVKGLTLYHLKSHLQKFRLGKQPHKEFNDHSIKDHASPLDLQRNSASSSAIIGRSMNEMQMEVQRRLHEQLEVQKHLQLRIEAQGKYMQSILEKACQTLAGENNMAAAAGSYKGNIANQSVNPXDMGSLKDFNSPLNFPSFQDLNIYGGDHLDLQQNLDRPPLDHCFMPNNDNNICLGPKKRPSPYSGSGKSPLIWSDDLRLQDLGTAASCLGPQDDPFKSSDSIQIAPPSIERGSEIDSISDIYESKPMLQGDDKKFDSANKLERPSPRRAPLGSDRMNPMINTGVRQGRNSPFGGIKSLGSYVGCDYQAMMKTGDSVTPRSQHTSXIDT

>MDP0000617021 MdGARP80

MAKSDSETQNPKDDRDEEPPSNVDGTVDGDPEPDEMDDEIDEEEDEDDDEEPPLPRKPLS

QEARLRAEKSKLENLVQRMSTERVDLRVHDVLIKGNTKTKEHLIEAELAGIKKATTMQEL

LEAAAIANAKLQQLEIFDAVRITLDSGPPELPGTTNVIVEVVETKSPISGXIGAYTKPAA

RSWTAEGTLKFKNLLGYGDLWDSSLAYGPNQTSELSAGLFLPRFKGFVTPMTARAFLLSQ

DWLEFSSYKERMMGLSLGLISSKHHDLAYNLGWRTLADPSQLASRSIRRQLGHELLSSIK

YTFKIDRRNSPLRPTRGYSFVSTSHIGGIAPDHRSTRFLRQEFDLRYAVPLGFYHAALNF

GISAGVIFPWGSGFLNKPSSLPERFFLGGDFSPVCTVGGPTTVWGFKTRGLGPTEPRRHV

GDNSNEENSESPGRDSVGGDLAVSAFADISFDLPLRWLKQHGVHGHIFAGAGNLAKLTEN

EFRNFSAPKFLQSFRSSVGAGIVVPTKFFRLEEAWCFDDAHKKKNNMRFPVLEHINTAIK

TVEKILKLEKKSEKQTFKYLISWDTLDAVSDINRLGMKSESTSIEEVSKSSPSTSKKEYD

HEDDEGEEDEVDEEGLQLRNNGGSSSNSTIEENHEKKGASGSVRQYVRSKTSRLRWTPDL

HLCFIRAVERLGGQERATPKLVLQLMNVKGLSIAHVKSHLQMYRSKKTEDHNQVVTDQAGFFMEGGDNQLYNLTQLSMLQSFNRWPSSRLRYGANDHSSWRGRHHQIYSPYSSSRTTALLDHNTRNIGLYGSVAERILGSNNTNIMSTSANQLYTNLPSPSHGQRNQIPWDELCQTMIHR

PCQDHHSWTAVIRDHQGQNMLKRKGLDTDNCDLDLNLSLKVPPKHDHGFGKGLQCGDDNHQKLLMGCSLSLTLSSSSSSKLGVKKLEGTGYGDGKHGKNMASTLDLTL

>MDP0000669078 MdGARP81

MGEEVRMSEYEGGGSGGDDGDDEERVSEWETGLPSADDLTPLSQPLIPIELASAFSISPE

PSRTAVDVNRASQKTVSTLRGGAHSQGFSSNYKSFDENRSDDVEPMIVEVDESGERYGSD

SRKSRKVDCSTEEADSTLRTENLSPXDTSARTLKRPRLVWTPQLHKRFVDVVAHLGIKNA

VPKTIMQMMNVEGLTRENVASHLQKYRLYLKRMQGLSGDVGPSXSDQLFATTPVPQSLHHESGGDCGGGSGPAGSGQSHGHGNGQFSFPMPYPPPGMMQMPVLGLSXGHGHMSMPGGAGXHGGGYHGFESHRYNMGSMVSYQHXASNDK

>MDP0000672486 MdGARP82

MGEEVRMSEYEGAGSGGDDEARVSEWEAGLPSADDLTPLSQPLIPIELASAFSISPETCH

TAFDVNRASHKTVSTLRGGAHSQGFSSSNYKSFDENRSDDVVEPMVVEVDESGERYGSDSRKSRKVDCSTEEADSALRTENFSPDDMSARTLKRPRLVWTPQLHKRFVEVVAHLGIKNAVPKTIMQLMNVEGLTRENVASHLQKYRLYLKRMQGLSGDVGSSSSDQLFATTPVPQSLRHESGGGGGGAGGSGQAGQGQSHGHANGQFSFPMPYPPAGMMQMPVLGMNHGHMGMPGGAGGHGGGYHGFESHHYNMGSMVSYQHAGSNDK

>MDP0000703817 MdGARP83

MESTXEMQTSAKLGFREYVKALEEERNKIQVFQRELPXCLELVTQAIERCRQQISDTTAD

YLHGQSECSEQTSSEGHVFEEFIPLKRISSSDSDEDEVQESQEAKDTEKDDKKKPDWLRS

VQLWNTTPDAPLKEELPRKALVMEVKRNGGAFQPFQREKSIGKTNGPVAKEPSSAPATSS

TTDTASGGSGGSNKKEEKDGQGQRKQRRNWSPELHRRFLHALQQLGGSHAATPKQIRELMKVDGLTNDEVKSHLQKYRLHTRRPTPTMHGNSNSGAPAPQFVVVGGIWVPPQEYAAMAATTASGEAANGIYAPLASTPAVTQVSPPIQRPRPKQPKPFHSDERVSHSEGRGHSNSTATSS

STHTPASPVV

>MDP0000773970 MdGARP84

MGSRRSESSAANKDRLRWTQELHDRFVEAVTKLGGPDRATPKGILKAMSVSGLTIYHIKS

HLQKYRISKFIPESTNKGKLQRKNITEMLPNFGTTSAAQLNEAFKIMQIQVQKNLQHKLE

SQGRFLERYSAERQNTNKNRPIVITKPRKAPLSKTSLPSLCDHSESNAKDFASDSEPDRS

ETQSSAEQFQTLKKLRLHHHQNDPNVQFALNSELYTAQSHSLLLPHEEDHISFPWNFAAC

SSPLAVSSCFL

>MDP0000809773 MdGARP85

MYSAIHSLPLDGHGDFGGSLDGTNLPGDACLVLTTDPKPRLRWTAELHERFVDAVTQLGG

PDTLKLYTCELWVFNVEATPKTIMRTMGVKGLTLYHLKSHLQKYRLGKLSCKDSAENSKDGIAASCIAESQDTGSSSAVSSRVIAQDLNDGYQVTEALRVQMEVQRRLHEQLEVQRRLQLRIEAQSKYLQSILEKACKALNDQAATAAGVEAAKEELSELAIRVSNDCEGIVPLDSTKIP

SLSEIAAALENRDVSNVMAHLGNCSVDSCLTSTGSPVLPMDMSSLAAAMKKRQRPFFGNGDSLPLESNMRQEVEWMMSNIG

>MDP0000826899 MdGARP86

MDAVSGGNSLNNPNLASKQRLRWTHELHERFIDAVVQLGGPDRATPKGVLRVMGVQGLTIYHVKSHLQKYRLAKYLPDSSSDGKKADKKEPGDVLSNLDGSSGMQITEALKLQMEVQKRLHEQLEVQRQLQLRIEAQGKYLKKIIEEQQRLSGVLSEAPGSGHLVRLSSDNCPESDNKTDPATPAPTSECPLQDKAAKECAPAKSISIDESFSSRREPLTPDSVCHVGSPAESPKAERLT

KKRRVNIGEAFSDPEVVLTHQILESSLDSSYQQGHTAFLPREQFHPSSGISFRNENQLEK

VAGSDM

>MDP0000846313 MdGARP87

MREDDESNWFSRWEEELPSPEELMPLSQTLITPDLALAFDISHPTPLLPHSQSPNXNPQP

PHHPPSPXLLLPNSLPPATPTHPNSSADFADSADLGSGAAGDEPARTLKRPRLVWTPQLH

KRFVDAVAHLGIKNAVPKTIMQLMSVDGLTRENVASHLQKYRLYLKRMQGLSGGSAGGAGXGAAGLAASADPATDHLFASSPVPAHFLHPHPGGRPNSEHFLPFVPVALQQHHHHQQQQQQQQIAAAVAAHPQYHRQVGHFGSPPNGQFEHPFLARQSSQPVHRMGAPLHNSGYVEDLESANASGGRKVLTLFPTGDD

>MDP0000869720 MdGARP88

MSASLTPIYGEAALKSRTLDLPFMGEEAVPKKILDLMNVEKLTRENVASHLQKYRLYLKR

ISCVANQQANMVAALGTSDSSYLRTASMNGVGNYHALTGPAQFHNNGYRSFPPNGMIGRLNTPAGVGLHCLPSSGMFQLGHAQNPSNSINDQPVMFPGNRNGNMLPAHLELDQQHSRGSTYDAPIYPNSSGFTDGKVTNRSNDPFGLTDNTLLRHTQDSEVGRNHVMLQSSVSMPSLKPELLPSFLDHRRLNDNWPTAAQSSAFRSNSFSARDSFKQPTLLDSRIGMQVPASTMSNNSGPTINNDLFMGWDEPKQDSPFRPDVMCSSMNNLMPVNDNVAPFDQNLDARSSAFQRNSDFNSIGQPDFLDVEISTMDAPLKLKQQGYLMDQRKPQGGYVPHNAGSLDELVSSMMKQLLGTLLAHLIAVNILVKGDKAKS

>MDP0000874064 MdGARP89

MELVSGGNSHGRRPPARRIDESSTEWTLEHHKLFVDAAVGLGLNDFKNDATPAATRTLIV

ELMSKNNTRDTKKIRFLRPVLIHHMREFQRMQKKTDDSLSADRMMRDFEGLNVNSDGNNNGGRMKEYFSPWTLELQKKLVEAVIQLGGVGKAIRPGGPCGILRLMDEERRRGLTEQMVKRRLWKLMDLQKKTRRATATLDGTHAASNMVDISRDLVRRHYATKGYLPIM

>MDP0000920870 MdGARP90

MERNIYGNCEGEGNCGYENGVMMTRDPKPRLRWTPDLHHRFVDAVTKLGGPHKATPKSVLRLMGLKGLTLYHLKSHLQKYRLGQHAQKHNSVEEQSRENNGDPYVHFSNHLSTGSSMKSSRGGNTEQGTLPLSEELKCQIEVQNRLQEQLEVQKKLQMRIEAQGKYLQAILEKAQNGLTLDVKVPDNIEATRAQLTDFNLALSNFMENMGEQDRGGNTIEMNDIYKKHNGSTFQIYEEGIREENKDTKLKVEGASIRFDLNSKGSYDYVGSNGGEFETRCFRLQDTTL

**Table S2 Sequences used for sequence alignment**

>MdHHO3

MESTEEMQASSKLGFREYVKALEEERHKIQVFQRELPLCFELVTQAIERCKQPVSDTTADYMHGQSECSQQTSSEGHVFEEFIPLKRTSSSDSEDDEVHESQEAKDNEKDDKIAGGDKKKSDWLRSVQLWNTTPDAPLKEELPRKALVMEVNRNGGAFQPFQKEKSIGKTSVPVAKQPSSAAATSSTTDTVSGGSGENNKKEEKDGLGQRKQRRNWSPELHRRFLNALQQLGGSHAATPKQIRELMKVDGLTNDEVKSHLQKYRLHTRRPTPTIRGNNNAAQAPQFVVVGGIWVPPQDYAAVAATTASGEAANNGIYAPVASTPAVTQVSPPVQRLRLKQPEPSHSDERVSHNEGRGHSNSTATSSSPHTPPSPAF

>AtHHO3 NP_563926.1

MIKKFSNMDYNQKRERCGQYIEALEEERRKIHVFQRELPLCLDLVTQAIEACKRELPEMT TENMYGQPECSEQTTGECGPVLEQFLTIKDSSTSNEEEDEEFDDEHGNHDPDNDSEDKNT KSDWLKSVQLWNQPDHPLLPKEERLQQETMTRDESMRKDPMVNGGEGRKREAEKDGGGGRKQRRCWSSQLHRRFLNALQHLGGPHVATPKQIREFMKVDGLTNDEVKSHLQKYRLHTRRPRQTVPNNGNSQTQHFVVVGGLWVPQSDYSTGKTTGGATTSSTTTTTGIYGTMAAPPPPQWPSHSNYRPSIIVDEGSGSHSEGVVVRCSSPAMSSSTRNHYVKNN

>NtLUX-Like XP_016469710.1

MMINNHENNYTEKMQRCQQYIDALEQERNKIQVFSRELPLCLELVTQAIETYKQQLSGTT TEYNVHTQSDVECSEEHTSSDVPILEEFIPLKGTFSHEDEDEDERDSHKSKTYNISDTSCSKDTKNSDKSCKKSDWLRSVQLWNNNQTPDPTPKEEVTPKKGAVVEVKKNGSGGAFHPFKKEKSAAATTEPPTSAAVAAAAASSTAETGSGSKKEEKDEKRKQRRCWSPELHRRFLQALQQLGGSHVATPKQIRELMKVDGLTNDEVKSHLQKYRLHTRR PSPSSINNNHQQPPQFVVVG

GIWVPPPEYSAMAAAAPSASGEASGVANSNGIYAPIATLP KGPLHDVSGGTLKQRQHNNK

PSRSSERGSGSHSDGGGVHSNSPATSSSTHTTTASPPY

>OsNIGT1 XP_015623779.1

MEVDHADRDGARRRCREYLLALEEERRKIQVFQRELPLCFDLVTQTIEGMRSQMDAAGSEETVSDQGPPPVLEEFIPLKPSLSLSSSEEESTHADAAKSGKKEEAETSERHSSPPPPPPEAKKVTPDWLQSVQLWSQEEPQQPSSPSPTPTKDLPCKPVALNARKAGGAFQPFEKEKRAELPASSTTAAASSTVVGDSGDKPTDDDTEKHMETDKDNDKDAKDKDKEGQSQPHRKPRRCWAPELHRRFLQALQQLGGSHVATPKQIRELMKVDGLTNDEVKSHLQKYRLHTRRPSSTGQSSAAAGVPAPPAPQFVVVGSIWVPPPEYAAAAAAQQHVQLAAAGNNASGSANPVYAPVAMLPAGLQPHSHRKQHQQQQQGQ RHSGSEGRRS GDAGDGSSSS PAVSSSSQTTSA

>PbEFM XP_009336613.1

MESTEEMQASSKLGFREYVKALEEEMHKIQVFKRELPLCYELVTHAIERCKQQVSDTTAD

YMHGQSECSQQTSSEGHVFEEFIPLKRTSSSDSEDDEVHESQEAKDNEKDDKIAGGDKKK

SDWLRSVQLWNTTPDAPLKEELPRKALVMEVNRNGGAFQPFQKEKSIGKTNGPVAKQPSSAAATSSTTDTVSGSSGENNKKEEKDGQGQRKQRRNWSPELHRRFLNALQQLGGSHAATPKQIRELMKVDGLTNDEVKSHLQKYRLHTRRPTPTMRSNNNAAHAPQFVVVGGIWVPPQDYAAVAATTASGEAANNGIYAPVASTPAVTQVSPPVQRLRLKQPEPSHSDERVSHSEGRGHSNSTATSSSTHTHISPAF

>PpEFM XP_007223224.2

MESTEQMQASRLGFRDYVKALEEERHKIQVFQRELPLCLELVTQAIERCKQQLSDTTTDY

MHGQSECSEQTSSEGHVFEEFIPLKRTSSSDSDDDEVQESQEPKTNDKDKTNGDKIKSDW

LRSAQLWNTTPDPPLKDELPRKALVMEVKRNGGAFQPFQREKSVGKTNRPVAKVPASAPATSSTTDTVSGGSGESHKKEEKDGQGQRKQRRNWSPELHRRFLHALQQLGGSHAATPKQIRELMKVDGLTNDEVKSHLQKYRLHTRRPTPTMHNNNNSDNNTQAPQFLVVGGIWVPPQDYAAVAATTASGEATRVAAANGIYAPVATSPSTVTPVSPPSLMQRPRPKRPESSHSDERVSHSEGRGHCNSTA TSSSTHTTAS PVL

>VvEFM XP_003631224.1

MDFSDKMQRCHDYIEALEEERRKIQVFQRELPLCLELVSQAIESCRQQMSGTTQEYFHGQ

SECSEQTSSDGPVLEEFIPIKKTSDDEDEQQSHQPNDNKDKNNDKSGKKSDWLRSVQLWN

QTPDPPVKEDTPKKIPSMEVKKNGGAFHPFKRDKAVGTNPTSAPSAATSSTAETATGCSS

GSRKEEKEGQSQRKARRCWSPELHRRFLHALQQLGGSHVATPKQIRELMKVDGLTNDEVKSHLQKYRLHTRRPNPAIQHNGNPQAPQFVVVGGIWVPPPEYTAVAATTSSGEATGVTTAN

GIYAPVASVPPSHPQGSTQRQQPMKPKKSQSEERGSHSEGGVQSNSPATSSSTHTTTTSPVF

>ZmNIGT1 NP_001131917.2

MEMDPPPPPPLPADRRRRFRDYLLALEEERRKIQVFQRELPLCLDLVTQSECPLSSVASS

SVHYHSIIHIVYVVCAAIEGMRSHMDSVVVGSEETVSDHGGPVLEEFMPLKPTTLSSSSS

QPQPQSQDDHDSAHYLEHRRAAAATANDVVDADKDGEAVGDLETAAARRLPHPETKKAMPDWLQSVQLWSNQQQPSASPPQHQDELLLPCRPVALNACRKPGGAFQPFEKEKKKKDKEEKQRAELELPLPLPAAASSAVVGDSCDRAGATDTDTDTAENNKASSTKGGKDKEAQLSSQSQAPSRKARRCWAPELHRRFLQALQQLGGSHVATPKQIRELMNVDGLTNDEVKSHLQKYRLHTRRPNSAAAVVQSGGTSVVAPPAAPQFVVVGGIWVPPPEYAAAVAAAAAAQPQVHLAGDASGTTTTTADKVYAPVATTLTAAPRPRPRPGRQSSSCSGARRCRDACSGSPAVSSSSSQTASA

>AtGLK1 NP_001189562.1

MLALSPATRDGCDGASEFLDTSCGFTIINPEEEEEFPDFADHGDLLDIIDFDDIFGVAGDVLPDLEIDPEILSGDFSNHMNASSTITTTSDKTDSQGETTKGSSGKGEEVVSKRDDVAAETVTYDGDSDRKRKYSSSASSKNNRISNNEGKRKVKTRLNEQVYNGFVFFLKVDWTPELHRRFVEAVEQLGVDKAVPSRILELMGVHCLTRHNVASHLQKYRSHRKHLLAREAEAANWTRKRHIYGVDTGANLNGRTKNGWLAPAPTLGFPPPPPVAVAPPPVHHHHFRPLHVWGHPTVDQSIMPHVWPKHLPPPSTAMPNPPFWVSDSPYWHPMHNGTTPYLPTVATRFRAPPVAGIPHALPPHHTMYKPNLGFGGARPPVDLHPSKESVDAAIGDVLTRPWLPLPLGLNPPAVDGVMTELHRHGVSEVPPTASCA

>AtGLK2 NP_199232.1

MLTVSPAPVLIGNNSKDTYMAADFADFTTEDLPDFTTVGDFSDDLLDGIDYYDDLFIGFDGDDVLPDLEIDSEILGEYSGSGRDEEQEMEGNTSTASETSERDVGVCKQEGGGGGDGGFRDKTVRRGKRKGKKSKDCLSDENDIKKKPKVDWTPELHRKFVQAVEQLGVDKAVPSRILEIMNVKSLTRHNVASHLQKYRSHRKHLLAREAEAASWNLRRHATVAVPGVGGGGKKPWTAPALGYPPHVAPMHHGHFRPLHVWGHPTWPKHKPNTPASAHRTYPMPAIAAAPASWPGHPPYWHQQPLYPQGYGMASSNHSSIGVPTRQLGPTNPPIDIHPSNESIDAAIGDV

ISKPWLPLPLGLKPPSVDGVMTELQRQGVSNVPPLP

>AtPHR1 NP_194590.2

MEARPVHRSGSRDLTRTSSIPSTQKPSPVEDSFMRSDNNSQLMSRPLGQTYHLLSSSNGGAVGHICSSSSSGFATNLHYSTMVSHEKQQHYTGSSSNNAVQTPSNNDSAWCHDSLPGGFLDFHETNPAIQNNCQIEDGGIAAAFDDIQKRSDWHEWADHLITDDDPLMSTNWNDLLLETNSNSDSKDQKTLQIPQPQIVQQQPSPSVELRPVSTTSSNSNNGTGKARMRWTPELHEAFVEAVNSLGGSERATPKGVLKIMKVEGLTIYHVKSHLQKYRTARYRPEPSETGSPERKLTPLEHITSLDLKGGIGITEALRLQMEVQKQLHEQLEIQRNLQLRIEEQGKYLQMMFEKQNSGLTKGTASTSDSAAKSEQEDKKTADSKEVPEEETRKCEELESPQPKRPKIDN

**Table S3 The primers used in this article**

| **The primers used for qRT-PCR in apple** | | |
| --- | --- | --- |
| RT-MdHHO3 | CAATTCGCGGCAACAACAAC | CATTATTGGCTGCCTCCCCG |
| RT-MdNRT1.1 | CTACCTCGGCCAATACCGTC | CACCGGTTGCTTGAATGGTG |
| RT-MdNRT2.1 | GGAGCTACCTTTGGCGTCAT | AGACAACCCTGAGGCAGTTG |
| RT-MdNYC1 | TCTTCGGTTTGGGGTGGTTT | AACGCCTGTGCTCATCATGG |
| RT-MdPAO | AACGCCTGTGCTCATCATGG | TCTTGGAGACCGAACAGCAC |
| RT-MdSGR1 | CCATATTTCCTGTTAATGCAAGGCT | AGGTGCTTCTTTTCGTCCACT |
| RT-MdNR | GACCGGGATCACCCCAATTT | ATCAAGTTCTTCCCGCAGCA |
| RT-MdNIR | GCTTCAGAGGGAACCGACAA | CGGAAGATGCTCTCACCAGG |
| RT-MdCIPK8 | TTTGGTCCTGCGGGGTTATC | GCTCCCACTGGAAACCAAGA |
| RT-MdCIPK23 | TGCTGCTAGATGTTAGCGGG | TGATTACCTCAGGGGCAACG |
| RT-MdNAR2 | TCGAAAGGTCGTACACCAGC | GTCTGACCATACGCCACCTC |
| RT-MdNLP6 | GCCGCGTTTTCCAGCATAAA | GGGCAAAGCTAAGGTTCCCT |
| RT-MdLBD13 | CCTCCAGTGGATCGAAACCC | AACCGGGTCGTTGGGAATC |
| **The primers used for qRT-PCR in arabidopsis** | | |
| AtNRT1.1 | GGACCGTCCACGCTCAATTA | GGATCTCGAAGCTCCCGATG |
| AtNRT2.1 | GCTTGCACGTTACCTGTGA | TCCTTGATGCATGTTCTTCTG |
| AtNRT2.4 | CTGGTGGAAACTTCGGCTCT | CCATCCATGTCAGCCCTTGT |
| AtNRT2.5 | GAATAGCCTCTGTCTCCGGC | GGCTAGACGTGGACCGAAAA |
| AtCYP735A2 | GATCCCTGTCCTTGCGATCC | CCAGCCGCAAAAGGCATAAA |
| AtIPT3 | CGGTTTCTGCTGGACATTGC | CACTAGACACCGCGACAACT |
| AtARR7 | CGGTTGGTGAGGTCATGAGG | GGCAAGAACATGCAACTCCG |
| AtNR | GTGATCCAGAGGACGAAACC | AACCTTCCTTTGCGATTTCA |
| AtNIR | TCGGGGGACGAATAGGAAGT | TCTCAGCCACCAATGGAACC |
| AtSGR | TCACATAAGCGGTGGCCATT | AAGCCTTCAACACCACAGGT |
| AtNYC | AGTAACGCTAGCGAGTCAGC | AGCTTCTCAACGTCTTCGGG |
| AtPAO | ATTCACTCCAACCCAGGCAG | GAGGTTGGTTAGACGGGGTG |
| **The primers used for qRT-PCR in tobacco** | | |
| RT-NtNRT1.1 | GACCGCGCTGTCGTTCC | TGAAAGGATTAAGCCAACTGCAATA |
| RT-NtNRT2.1 | CCGAAGCTGAGAGGCAGAAA | GAGTAGGTGCTGATCCCACG |
| RT-NtNR | GGTGGGGTACTTCGAGTTGG | GAGAAATGACCCTAACGGCA |
| RT-NtNIR | TTGCGGGCAAGCCATAATTG | ATCCTCACCGGCTTTGTCAC |
| RT-NtSGR | GGTAGGTGGGAAAATGGGAAA | ACTCATTGGGGGAAAGCAACA |
| NtNYC1 | TATGCTGCAGAGGCAGATCG | CCGAGAGCGAGAACACAGAG |
| NtPAO1 | TCTGATTGGCGCAGTAGTGG | CGTATGCCAAACCAGCACTT |
| **The primers used for subcellular localization** | | |
| MdHHO3-pZP211 | GGATCCATGGAGTCCACAGAGGAAATGC | GTCGACAAACGCAGGGGATGGAGGGGTGT |
| **The primers used for transgene** | | |
| MdHHO3-pRI101 | CATATGATGGAGTCCACAGAGGAAATGC | GAATTCTTAAAACGCAGGGGATGGAGGG |
| **The primers used for Y1H** | | |
| MdHHO3-pGADT7 | CATATGATGGAGTCCACAGAGGAAATGC | GAATTCTTAAAACGCAGGGGATGGAGGG |
| MdNRT2.1-pABAi | GAGCTCTGTACAGTTGTAGCCCATGCTA | GTCGACAACTCAGAAACTGCTTAGAGTAC |
| **The primers used for LUC assay** | | |
| MdHHO3-pGreenII 62-SK | GGATCCATGGAGTCCACAGAGGAAATGC | GAATTCTTAAAACGCAGGGGATGGAGGG |
| MdNRT2.1-GreenII 0800-LUC | GTCGACTGTACAGTTGTAGCCCATGCTA | GAGCTCAACTCAGAAACTGCTTAGAGTAC |
| **The probes used for EMSA assay** | | |
| MdHHO3-pGEX4T-1 | GGATCCATGGAGTCCACAGAGGAAATGC | GTCGACTTAAAACGCAGGGGATGGAGGG |
| MdHHO3-probe 1 | ATTTCATTAA**GATTC**TTAGGGAGCG | CGCTCCCTAA**GAATC**TTAATGAAAT |
| MdHHO3-probe 2 | CTGCC**GAATC**TGAACGCGCAGCTCCATG **GAATC**GCCGCCCATCCA**GAATC**CCGGG | CCCGG**GATTC**TGGATGGGCGGC**GATTC**CAT GGAGCTGCGCGTTCA**GATTC**GGCAG |
| MdHHO3-probe 3 | GAACGTTTCA**GAATC**CCATTGGCCA | TGGCCAATGGGATTCTGAAACGTTC |
| MdHHO3-probe 4 | AAAGC**GAATC**AAATGCGAATACCATCG AATATTCAACCTCCGACA**GATTC**GTTGT | ACAAC**GAATC**TGTCGGAGGT TGAATATTCG ATGGTATTCGCATTT**GATTC**GCTTT |
| MdHHO3-probe 5 | TCCGTTTTGT**GATTC**TGCTTGGATC | GATCCAAGCA**GAATC**ACAAA ACGGA |
| MdHHO3m-probe | ATTTCATTAA**CATTG**TTAGGGAGCG | CGCTCCCTAA**CAATG**TTAATGAAAT |
| MdNRT2.1-probe 1 | TCTTCTCTCG**GATTC**TCCACCGTGA | TCACGGTGGA**GAATC**CGAGAGAAGA |
| MdNRT2.1-probe 2 | AACTCTTAAC**GAATC**AATGGATGGA | TCCATCCATT **GATTC**GTTAA GAGTT |
| MdNRT2.1-probe 3 | TTCTCAAACT**GAATC**TTCCCTCTTC | GAAGAGGGAA**GATTC**AGTTTGAGAA |
| MdNRT2.1-probe 4 | AGTTAATTAG**GATTC**ATTGACTTAA | TTAAGTCAAT **GAATC**CTAAT TAACT |
| MdNRT2.1-probe 5 | AAAGTATTCA**GAATC**TAACTCTTGC | GCAAGAGTTA**GATTC**TGAATACTTT |
| MdNRT2.1m-probe | TCTTCTCTCG**CATTG**TCCACCGTGA | TCACGGTGGA**CAATG**CGAGAGAAGA |
